# Operational manifolds in spiking neural networks

**DOI:** 10.3389/fnins.2026.1755119

**Published:** 2026-02-18

**Authors:** Szymon Mazurek, Jakub Caputa, Piotr Maj, Maciej Wielgosz

**Affiliations:** 1 Department of Computer Science, Electronics and Telecommunications, AGH University of Krakow, Krakow, Poland; 2 Department of HPC Software, Academic Computer Centre Cyfronet AGH, Krakow, Poland; 3 Computational Neuroscience Group, Sano Centre for Computational Medicine, Krakow, Poland; 4 Department of Metrology and Electronics, Faculty of Electrical Engineering, Automatics, Computer Science and Biomedical Engineering, AGH University of Krakow, Krakow, Poland; 5 Instrumentation Department, Brookhaven National Laboratory, Upton, NY, United States

**Keywords:** inference-time state handling, leaky integrate-and-fire parameters, neuromorphic computing, neuron hyperparameters, operational manifold, robustness and stability, spiking neural networks

## Abstract

Spiking Neural Networks (SNNs) can be more energy-efficient than conventional deep networks, but their performance and stability depend strongly on neuron hyperparameters and inference-time state handling. Here we study how leaky integrate-and-fire (LIF) parameters and deployment policies jointly shape operating regimes, accuracy–energy trade-offs, and robustness. We introduce the notion of an *operational manifold*: a contiguous region in neuron hyperparameter space where spiking activity remains balanced (neither silent nor saturated) while task performance is maintained. Focusing on the membrane time constant (τ_*m*_) and firing threshold (*V*_th_), we map this manifold via systematic grid sweeps across multiple architectures and datasets. To quantify efficiency, we estimate synaptic operation (SOP) cost during inference and define composite scores that couple normalized accuracy with SOP-based energy proxies, enabling the identification of accuracy–energy frontiers within the manifold. We further examine inference-time state handling by comparing *reset* and *carry* policies for membrane potentials. On static, i.i.d. inputs, reset improves accuracy for short inference windows, while carry introduces cross-sample interference that diminishes as the integration horizon grows, highlighting the importance of state management in streaming deployments. Furthermore, we analyze robustness through progressive input perturbations and show that leaving the operational manifold is accompanied by increased inter-neuronal spike-train correlations and more synchronized firing. Summary statistics of correlation distributions (including skewness, kurtosis, and tail mass) provide informative, label-free indicators of noise exposure and internal instability. Together, these results provide practical guidance for selecting neuron hyperparameters and inference policies that achieve energy-efficient and stable SNN operation, and they suggest correlation-based diagnostics as lightweight health monitors for deployed neuromorphic systems.

## 1 Introduction

The transition from second-generation Artificial Neural Networks (ANNs) to third-generation Spiking Neural Networks (SNNs) marks a shift from continuous-valued, clocked activations to discrete, event-driven dynamics. By encoding information in sparse spatiotemporal spike trains and communicating asynchronously, SNNs promise substantially lower power consumption than conventional deep learning models, especially when mapped to neuromorphic hardware that exploits local memory and event-driven computation (Roy et al., 2019; Christensen, 2022; Huynh et al., 2022). In contrast to standard ANNs, where units are typically memoryless nonlinearities, spiking neurons are governed by time-dependent dynamical equations parameterized by biophysical hyperparameters—most prominently the membrane time constant (τ_*m*_) and firing threshold (*V*_th_).

Training methods for SNNs have advanced rapidly, with surrogate gradient techniques now enabling gradient-based optimization at scale (Neftci et al., 2019). However, a principled characterization of how neuron-level hyperparameters shape global network dynamics and task performance is still lacking. In practice, τ_*m*_ and *V*_th_ are often treated as fixed design choices or tuned through a black-box search, with little insight into their joint effect on spiking regimes. Mismatches between excitation dynamics and thresholding can push the network into *silent* regimes, where activity fails to propagate, or into *saturated* regimes with tonic, epilepsy-like firing. Both failure modes erode the anticipated energy advantages of SNNs by either wasting representational capacity or incurring unnecessary spike and synaptic operation (SOP) cost.

In this work, we formalize and empirically study an **operational manifold**: a contiguous subspace of neuron and simulation hyperparameters θ = (τ_*m*_, *V*_th_, …) in which the network maintains a homeostatic balance between quiescence and saturation while preserving task performance. We argue that operating within this manifold is not only necessary for accuracy, but also the key determinant of the energy-accuracy trade-off, as firing statistics simultaneously govern representational richness and SOP budgets.

We also address the temporal structure of the deployment. Most SNN benchmarks assume finite-length samples and hard resets of internal state between inputs, reflecting i.i.d. static datasets such as MNIST or CIFAR-10. In neuromorphic sensing applications, however, inputs arrive as continuous, temporally correlated streams. We therefore compare *reset* and *carry* (no reset) membrane-potential policies at inference time, quantifying how they affect accuracy, stability, and energy consumption across static and event-based datasets. This highlights a fundamental tension between the memoryless assumptions of standard evaluation protocols and the inherently stateful nature of realistic SNN deployments.

Finally, we use the operational manifold as a lens on robustness and data drift. In biological circuits, deviations from homeostasis are known to manifest as changes in correlation structure and hypersynchronous activity, reflecting a breakdown of excitation-inhibition balance (Turrigiano, 2012; Marder and Goaillard, 2006; Vogels et al., 2011; Denève and Machens, 2016; Chen et al., 2022; Beggs and Plenz, 2003). Motivated by this perspective, we analyze pairwise spike-train correlations in the deep layers of trained SNNs. We show that pushing the network outside its operational manifold—via poorly tuned hyperparameters or corrupted inputs—induces characteristic shifts in the distribution of correlation coefficients, which can be exploited as label-free indicators of concept drift and internal instability.

This paper makes three contributions:

- We introduce the notion of an *operational manifold* for SNNs and empirically map it in the (τ_*m*_, *V*_th_) space for multiple architectures and datasets, identifying a balanced band of activity that jointly optimizes task performance and firing sparsity.
- We define composite efficiency metrics that couple accuracy with SOP-based energy proxies. We use them to derive practical guidelines for selecting operating points and timestep budgets, including the choice of reset versus carry policies under static and streaming input conditions.
- We propose correlation-based diagnostics that monitor the internal spike-train structure under progressive input perturbations. Higher-order statistics of correlation distributions (e.g., skewness, kurtosis, and tail mass) serve as robust, label-free indicators of manifold violations, enabling drift detection, and health monitoring for deployed neuromorphic systems.

## 2 Related work

SNNs are vastly different from ANNs in many aspects, thus necessitating different reasoning and approaches (Niu et al., 2023). While ANNs make discrete predictions on a given input sample, SNNs produce temporal spike trains, which must be interpreted in terms of the predicted phenomena, requiring specialized readout techniques. However, most approaches utilize the so-called hard reset, where the network's internal state is reset to baseline after a pre-defined period. While convenient for experimentation, such an approach seems incompatible with real-world applications that receive inputs continuously, and setting the reset interval is often impossible. While important, this problem is not yet widely explored. A recent study by (Zhang 2025) draws inspiration from state-space ANN models in an image recognition task. (Han et al. 2020) noted that fixing the reset potential leads to information loss when processing input with SNNs. However, these works do not focus on *when* to apply the reset, but rather on what membrane potential the neuron should move to *after* the reset. The need for continuous output processing was also acknowledged and explored in the ANN domain, specifically with recurrent neural networks (Yin and Corradi, 2025).

Due to their inherent stateful nature and time-dependent dynamics, SNNs can be analyzed as nonlinear dynamical systems, thus implying the concept of activity stability. It is controlled by hyperparameters that define the neuron models. Zhang et al. show that the concept of SNN stability and dynamical control is crucial to achieving robust performance (Zhang et al., 2021). Research also shows that with more complex neuron models, a lack of proper activity regularization can lead to unstable network behavior (Strack et al., 2013). Insights from neurobiology supplement these views, showing the importance of homeostatic plasticity in stabilizing network activity (Chen et al., 2022; Turrigiano, 2011, 2012).

Several prior studies have implicitly explored such stability-constrained operating regimes in SNNs by analyzing how intrinsic neuron parameters shape feasible network behavior, without formalizing these regimes as a unified concept. (El-Allami et al. 2021) has shown that robustness to adversarial perturbations strongly depends on firing thresholds and temporal integration windows, revealing parameter-dependent stable operating regions. Similarly, (Ding et al. 2025) demonstrated that successful deep SNNs training requires specific parameter configurations that enable stable gradient flow and sufficient spiking activity.

Another important aspect of the deployment of SNNs in real-world scenarios relates to tracking concept drift, an event in which input data streams start to deviate from the distribution on which the networks were trained (Suárez-Cetrulo et al., 2023). This often leads to the degradation of prediction quality. Concept drift is widely explored in the domain of deep learning. Primary approaches focus on the analysis of input data and monitoring the internal state of the network, the latter being closely related to the techniques presented in this work. Recent research has shown effective methods for concept drift detection by directly analyzing embedding space activations (Greco et al., 2025; Ayers et al., 2025), using proxy models to monitor activations (Hu et al., 2025), or measuring model uncertainty (Baier et al., 2021). SNNs have traditionally seen little exploration regarding concept drift, though new research is finally addressing this gap. Bodyanskii and Savenkov proposed a dedicated SNN trained to detect concept drift explicitly (Bodyanskiy and Savenkov, 2024). Concept drift can also be mitigated by native SNN learning algorithms, such as spike time dependent plasticity (STDP), which continuously updates the network weights, thus adapting to new patterns in the data (Masquelier and Thorpe, 2007). However, STDP often shows limited usability when training complex SNNs, thus requiring further investigation and adaptation (Lu and Sengupta, 2024). Despite the lack of a large body of literature on this topic, biological insights suggest that the analysis of internal network spiking patterns may exhibit distinctive characteristics when encountering unfamiliar data (Dean et al., 2008; Nassar et al., 2021), prompting us toward the exploration shown in this work.

Recent advances also target latency, memory, and energy: Temporal-reversible SNNs selectively activate temporal dynamics to achieve O(L) training memory and constant-time inference pathways (Hu et al., 2024). Direct-input encoding and leak/threshold optimization reduce timesteps and latency (Rathi and Roy, 2023). Bayesian fusion leverages early priors for accelerated inference (Habara et al., 2024). Event-driven regularization and cutoff prune unnecessary activity during inference (Wu et al., 2025). Our work complements these by focusing on *neuron hyperparameters* as control knobs to position models on favorable accuracy–energy frontiers and by introducing correlation-based diagnostics.

## 3 Materials and methods

### 3.1 Operational manifold: definition and intuition

SNNs exhibit a high degree of sensitivity to neuron-level hyperparameters, such as the membrane time constant (τ_*m*_) and firing threshold (*V*_*th*_). These parameters jointly govern how information flows through time, shaping both the sparsity of spikes and the stability of internal dynamics. Across architectures and datasets, we consistently observe a bounded region of the (τ_*m*_, *V*_*th*_) space within which networks sustain meaningful activity and achieve stable accuracy–energy trade-offs. We refer to this region as the *operational manifold*—a contiguous subspace where neurons operate in a balanced regime between silence and saturation.

Conceptually, the manifold represents a physiological homeostasis analogous to cortical firing balance: neurons fire sparsely enough to remain energy-efficient, yet densely enough to propagate information reliably. Outside this manifold, activity either collapses (vanishing spikes) or explodes (tonic spiking), leading to failure modes reminiscent of over- or under-excitation in biological circuits. The presumed presence of such a space is prompted by the existence of Griffiths phases, a critical-like, broader regions in which the brain activity resides (Moretti and Muñoz, 2013).

Formally, for each trained configuration θ = (τ_*m*_, *V*_*th*_, … ) we define the mean network firing rate:


$$\begin{array}{l} \bar{r}\left(\theta \right)=\frac{1}{N}\underset{\ell ,i}{\sum }\frac{1}{T}\overset{T}{\underset{t=1}{\sum }}S_{\ell ,i}^{t}, \end{array}$$ (1)

and empirical accuracy *A*(θ). In our experiments, we limit the search space, exploring the effect of changes only in hyperparameters τ_*m*_ and *V*_*th*_. Therefore, remaining hyperparameters are treated as constant, simplifying configuration into θ = (τ_*m*_, *V*_*th*_). The operational manifold M is the subset of parameter space satisfying:


$$\begin{array}{l} r_{\min}\leq \bar{r}\left(\theta \right)\leq r_{\max},\;A\left(\theta \right)\geq A_{\text{thr}}, \end{array}$$ (2)

where (*r*_min_, *r*_max_) delimits silent and saturated regimes, and *A*_thr_ ensures functional performance.

In Equations 1, 2, θ is the hyperparameter tuple (τ_*m*_, *V*_*th*_); $S_{\ell ,i}^{t}\in \left\{0,1\right\}$ indicates whether neuron *i* in layer ℓ spikes at time *t*; indices are ℓ (layer), *i* (neuron), and *t* = 1…*T* (step); $N=\underset{\ell }{\sum }n_{\ell }$ is the total neuron count considered. The quantity $\bar{r}\left(\theta \right)$ is the mean spike rate per neuron per timestep, *A*(θ) is the measured task accuracy, and M collects configurations that satisfy both $r_{\min}\leq {\bar{r}}_{\max}$ and *A*_thr_, where *r*_min_ and *r*_max_ bound, respectively, *silent* and *saturated* regimes, and *A*_thr_ is a task-driven minimum acceptable utility.

Intuitively, the rate window excludes degenerate dynamics (too few or too many spikes), while the accuracy floor removes trivial low-energy but non-functional settings; together, they select a band in (τ_*m*_, *V*_*th*_) with good accuracy–energy trade-offs.

The proposed description of the operational manifold thus serves as a unifying lens through which we interpret the network's functional state: it links low-level biophysical hyperparameters to emergent population dynamics and task-level performance. In practical terms, it provides a principled criterion for distinguishing functional configurations from degenerate ones, enabling systematic navigation of the hyperparameter space without relying on *ad-hoc* tuning. Conceptually, this highlights that effective SNN operation is not tied to a single optimal point but rather to an extended band of balanced excitability, reminiscent of the homeostatic operating ranges observed in cortical circuits. In biological networks, firing rates are stabilized by a combination of synaptic and intrinsic homeostatic plasticity mechanisms and by excitation-inhibition balance, which together keep population activity within functional regimes despite ongoing noise and perturbations (Turrigiano, 2012; Marder and Goaillard, 2006; Vogels et al., 2011; Denève and Machens, 2016; Chen et al., 2022).

**Figure 1:**
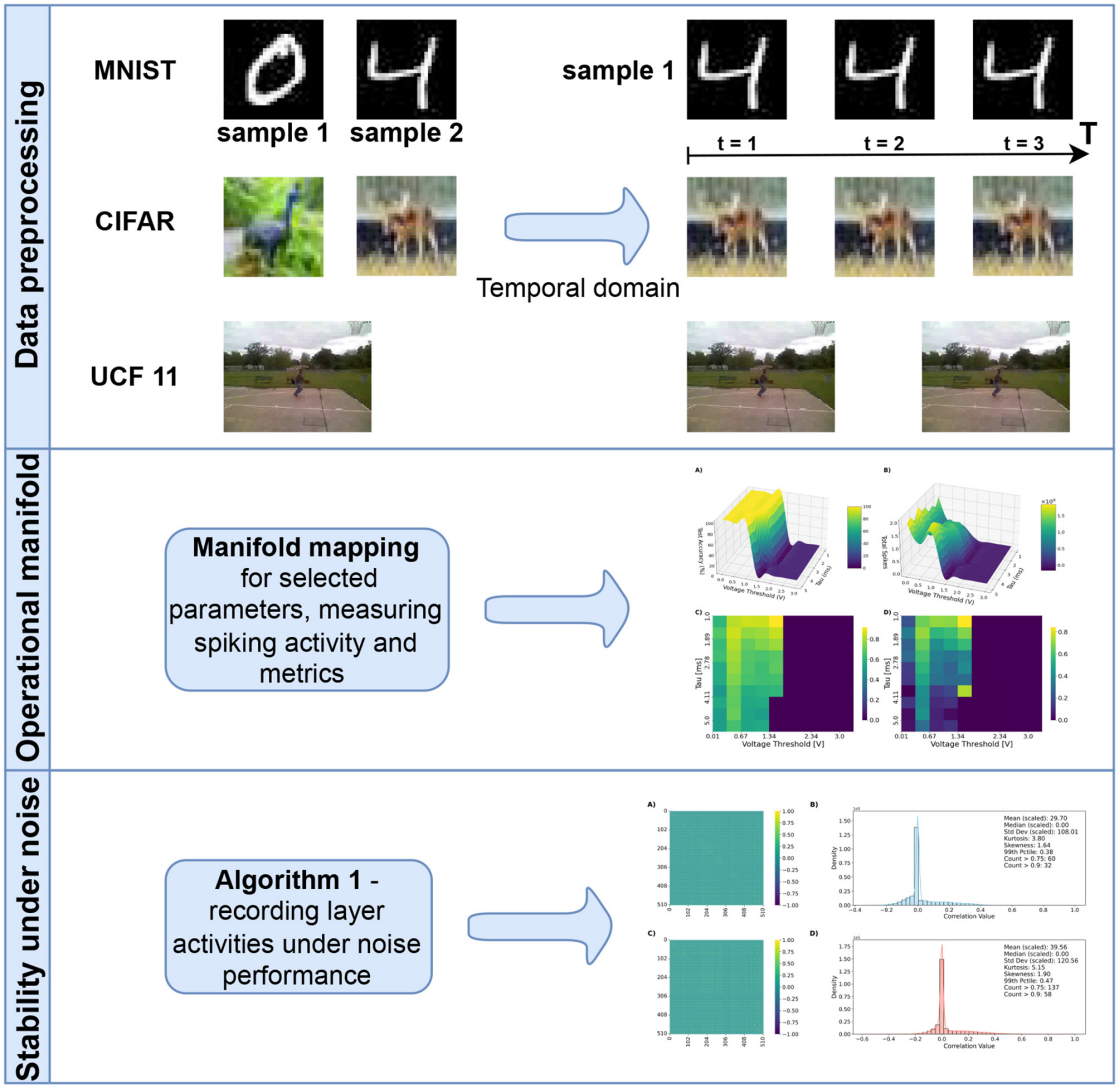
Flowchart of the manuscript.

This viewpoint (Figure 1) motivates the subsequent analyses, where we explore how the manifold structure emerges across datasets, architectures, and inference policies, and how it can guide the design of efficient, robust neuromorphic systems.

### 3.2 Determining the boundaries of the Operational Manifold

Algorithm 1 formalizes how we construct the *Operational Manifold* as a subset of neuron hyperparameter configurations that yield both (i) balanced spiking activity and (ii) acceptable task performance. We denote by θ ∈ Θ a candidate configuration (e.g., θ = (τ_*m*_, *V*_*th*_, …)), where Θ is obtained either by a regular grid sweep or by sampling in the multi-dimensional hyperparameter space. For each θ, we run an evaluation (or train-then-evaluate) step to measure two summary quantities: the mean firing rate $\bar{r}\left(\theta \right)$ (averaged over neurons and time during inference) and the task accuracy *A*(θ).

Algorithm 1 **Identify operational manifold boundaries.**

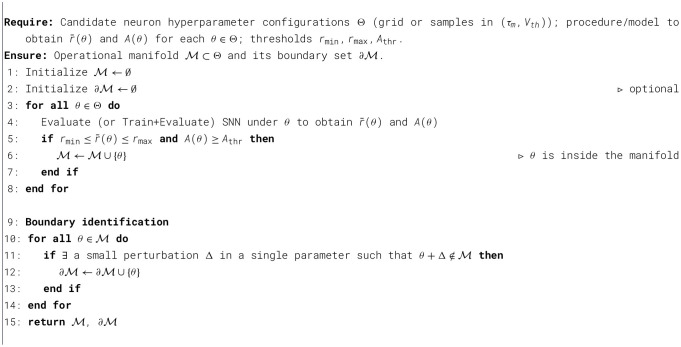

To characterize the *boundary* of the manifold, Algorithm 1 optionally identifies a subset ∂M of configurations that are sensitive to small one-dimensional perturbations. Concretely, a point θ∈M is marked as a boundary point if there exists a sufficiently small change Δ to a *single* parameter (e.g., perturbing only τ_*m*_ or only *V*_*th*_) such that the perturbed configuration θ+Δ violates at least one membership inequality and thus falls outside M. In a discretized grid, this corresponds to checking whether any immediate neighbor of θ (along a coordinate axis) lies outside the manifold. This procedure yields ∂M, which can be used to visualize the transition between silent/saturated regimes and the stable operational regime.

In two dimensions, M can be visualized as a contiguous region on a heatmap over (τ_*m*_, *V*_*th*_). In higher dimensions, the same construction defines a hyper-surface (or hyper-volume) in the augmented parameter space. In practice one can report 2D slices, projections, or summary statistics of M and ∂M.

### 3.3 Neuron and network model

We consider leaky integrate-and-fire (LIF) dynamics per neuron *i* in layer ℓ:


$$\begin{array}{l} V_{\ell ,i}^{t+1}=\left(1-\alpha \right)V_{\ell ,i}^{t}+I_{\ell ,i}^{t}-V_{\text{th}}\cdot S_{\ell ,i}^{t},\;\alpha =\frac{\text{Δ}t}{\tau_{m}}, \end{array}$$ (3)

$$\begin{array}{l} S_{\ell ,i}^{t}=⊮\left[V_{\ell ,i}^{t}\geq V_{\text{th}}\right],\;V_{\ell ,i}^{t+1}\leftarrow V_{\ell ,i}^{t+1}\left(1-S_{\ell ,i}^{t}\right)+V_{\text{reset}}S_{\ell ,i}^{t}. \end{array}$$ (4)

Here, $V_{\ell ,i}^{t}$ is the membrane potential, $I_{\ell ,i}^{t}$ the synaptic input current (optionally low-pass filtered), $S_{\ell ,i}^{t}\in \left\{0,1\right\}$ a spike indicator (ℍ[·]), α = Δ*t*/τ_*m*_ with Δ*t* the integration step and τ_*m*_ the membrane time constant, *V*_th_ the firing threshold, *V*_reset_ the reset potential, indices ℓ, *i* denote layer and neuron, and *t* = 1, …, *T* the discrete timesteps.

We simulate LIF neurons with a forward–Euler step (thus α ∈ (0, 1) sets per-step leak), include an absolute refractory period *t*_ref_, and support *hard* reset (clamp *V*_reset_) and *soft* reset (subtract *V*_th_), using hard reset by default; membranes initialize near *V*_reset_.

### 3.4 Energy proxies and efficiency scores

To compare hyperparameter settings in a way that reflects the intrinsic accuracy–energy trade-off of SNNs, we introduce two composite efficiency scores based on normalized accuracy and SOP cost. Since accuracy and energy are jointly shaped by firing dynamics, these metrics summarize the quality of an operating point rather than treating accuracy or spike count in isolation. Both efficiency scores depend on user-chosen trade-off parameters that modulate how strongly energy (or accuracy) influences the final value. These parameters, therefore, allow practitioners to shape the operating objective depending on deployment needs while keeping the underlying accuracy–energy trade-off grounded in measurable spike dynamics. Their summary and intuitive explanation can be found in Table 1.

**Table 1:** Efficiency metrics and interpretation of their trade-off parameters.

**Metric**	**Parameter role**	**Practical effect**
BES_λ_	λ sets the relative weight of energy vs. accuracy. Higher values penalize energy more strongly.	Favors either accuracy (low λ) or energy efficiency (high λ). Highlights broad regions with stable trade-offs.
EAS_β_	β adjusts the emphasis between accuracy and energy, analogous to the *F*_β_ score. β>1 stresses accuracy; β < 1 stresses energy.	Identifies configurations closest to the accuracy–energy Pareto frontier. Produces sharper optima than BES.

We estimate computational cost via synaptic operations (SOP):


$$\begin{array}{l} \text{SOP}=\underset{l}{\sum }\overset{T}{\underset{t=1}{\sum }}N_{\text{in}}^{\left(l\right)}\left(t\right)\cdot f_{\text{fanout}}^{\left(l\right)} \end{array}$$ (5)

Where $N_{\text{in}}^{\left(l\right)}\left(t\right)$ is the total number of spikes entering layer *l* at timestep *t*, and $f_{\text{fanout}}^{\left(l\right)}$ is the synaptic fan-out of the input connections into that layer.

Over a given hyperparameter sweep, we normalize energy and accuracy to [0, 1] using the observed extrema:


$$E_{\text{norm}}=\frac{\text{SOP}-{\text{SOP}}_{\min}}{{\text{SOP}}_{\max}-{\text{SOP}}_{\min}},\;A_{\text{norm}}=\frac{\text{Acc}-{\text{Acc}}_{\min}}{{\text{Acc}}_{\max}-{\text{Acc}}_{\min}}.$$

We then report two scalar scores that summarize the accuracy–energy trade-off:


$$\begin{array}{l} {\text{BES}}_{\text{λ}}=A_{\text{norm}}-\text{λ}E_{\text{norm}},\text{ λ}\in \left[0,1\right], \end{array}$$ (6)

$$\begin{array}{l} {\text{EAS}}_{\beta }=\frac{\left(1+\beta^{2}\right)A_{\text{norm}}\left(1-E_{\text{norm}}\right)}{\beta^{2}A_{\text{norm}}+\left(1-E_{\text{norm}}\right)},\;\beta >0. \end{array}$$ (7)

Both efficiency scores depend on user-chosen trade-off parameters that modulate how strongly energy (or accuracy) influences the final value. These parameters therefore allow practitioners to shape the operating objective depending on deployment needs, while keeping the underlying accuracy–energy trade-off grounded in measurable spike dynamics.

### 3.5 Datasets

In all experiments, we evaluate on two static-image benchmarks—MNIST (28 × 28 grayscale, 10 classes) (LeCun et al., 2010) and CIFAR-10 (32 × 32 RGB, 10 classes) (Krizhevsky, 2009). We also assess video classification dataset UCF11 (resized 128 × 128 RGB video frames, 11 classes) (Reddy and Shah, 2012), as it includes more complex temporal dependencies between frames. In experiments on input perturbation, we additionally use an event-stream benchmark derived from neuromorphic sensors: N-MNIST/MNIST-DVS (Orchard et al., 2015; Serrano-Gotarredona and Linares-Barranco, 2015), further referenced as EventMNIST.

Static datasets are Poisson encoded over *T* = 10 timesteps, whereas EventMNIST is fed as native DVS spike streams discretized into *T* bins with polarity separation in the channel dimension. We follow standard splits (MNIST/EventMNIST 60k/10k; CIFAR-10 50k/10k). In UCF11 dataset, we use approximately 20% of recording groups in a given class as test data.

### 3.6 Architectures

We evaluate two primary backbone families: MLP-SNN and ConvSNN. To ensure experimental consistency, all convolutional and fully connected layers (excluding the output) utilize Batch Normalization (BN) applied before the spiking activation function. Across all layers in both architectures, neurons share identical membrane time constants τ_*m*_ and threshold voltages *V*_*th*_. The MLP-SNN is a feedforward network featuring three hidden layers (512, 256, and 64 neurons). The ConvSNN follows a VGG-like design comprising two convolution-BN-LIF-pooling blocks. All convolutional layers employ 3 × 3 kernels with a stride of 1 and padding of 1, followed by 2 × 2 Max Pooling with a stride of 2. The first block outputs 64 channels, and the second outputs 128 channels. To reduce parameter count, we apply Global Average Pooling after the final convolutional block, resulting in a 128-dimensional vector feeding into the classifier head (128–64–10 neurons). We additionally consider two deep convolutional networks: SpikingResnet18 (Hu et al., 2023) and SpikingVGG11 (Sengupta et al., 2019) to determine if observed properties persist in complex networks. Decisions are made by accumulating output spike trains as an average across time window *T* = 10. We train SNNs with arctangent surrogate gradients and optimize cross-entropy loss with Adam optimizer (Kingma and Ba, 2015).

In noise perturbation experiments, we test six architectures: MLP-SNN, ConvSNN, their recurrent counterparts, and previously used deep networks: SpikingResnset18 and SpikingVGG11. In the recurrent variants of simple networks, the final hidden linear layer is enhanced with recurrent connections, as research suggests they may influence system stability (Ozeki et al., 2009; Eskikand et al., 2023). In the two deep networks, penultimate linear layer was modified to consist of 512 output neurons, reducing the complexity of experimental analyses. Additionally, SpikingVGG11 was modified by removing the last two Conv-Pool blocks to reduce computational complexity. The neuron model, surrogate function, optimizer and sample length *T* remain unchanged.

A summary of the custom architectures is provided in Table 2. For clarity, we do not describe the SpikingVGG11 and SpikingResnet18 architectures, as they are established in the literature (Sengupta et al., 2019; Hu et al., 2023).

**Table 2:** Detailed architecture specifications for the custom SNN models used in experiments.

**Model**	**Layer sequence**	**Parameters**
**ConvSNN**		
	1. Conv → BN → LIF → MaxPool	3 × 3 kernel, 64 ch, stride 1; Pool 2 × 2
	2. Conv → BN → LIF → MaxPool	3 × 3 kernel, 128 ch, stride 1; Pool 2 × 2
	3. Global avg pool	Output size: 128 × 1 × 1
	4. Linear → BN → LIF	128 → 64 neurons
	5. Linear (output)	64 → 10 neurons
**MLP-SNN**		
	1. Linear → BN → LIF	784 → 512 neurons
	2. Linear → BN → LIF	512 → 256 neurons
	3. Linear → BN → LIF	256 → 64 neurons
	4. Linear (output)	64 → 10 neurons

BN, Batch Normalization; LIF, Leaky-Integrate-Fire neuron layer.

### 3.7 Operational space mapping

To map the operational space, we perform uniform grid sweeps over neuron hyperparameters with τ_*m*_ ∈ [1.001, 5]ms and *V*_th_ ∈ [0.01, 3] V, using 10 uniformly spaced points for each parameter. For the MLP-SNN MNIST, the sweep range was extended (τ_*m*_ ∈ [1.001, 10]ms and *V*_th_ ∈ [0.01, 10] V) because within the initial bounds the network never entered a clearly silent or saturated regime, and no collapse of activity was observed, making a wider search necessary to capture the manifold boundaries. For every parameter pair, we measured test accuracy, spike statistics, and the SOP-based energy proxy. We deliberately abstain from using more advanced search methods like Bayesian search, as we want to show the entire parameter space behavior, not a single, optimal point.

### 3.8 Reset vs. carry policies

During inference in SNNs, the treatment of membrane potentials between successive inputs can substantially influence both prediction accuracy and energy efficiency. In this work, we compare two policies: *reset* and *carry* on MNIST dataset using MLP-SNN architecture.

Under the reset policy, all neuron membrane potentials are reinitialized to *V*_reset_ prior to each new input for both training and inference. This removes any residual state and eliminates cross-sample interference, which is particularly appropriate for datasets such as MNIST. A full reset, however, may increase transient noise at the beginning of the simulation window and reduces temporal continuity across inputs.

Under the carry policy, the final membrane states *V*^*T*^ are propagated to the next input, effectively endowing the network with short-term memory across samples. At the same time, it can introduce interference between unrelated samples and may degrade classification performance when the temporal window *T* is short.

The network is trained using either policy with constant sample length *T* = 10. Training parameters remain the same as in previous experiments. Afterwards, we perform inference on the test set, modifying the sample length *T*.

First, we calculate average accuracy achieved by network under given reset policy for given inference sample length *T*. In addition, we are accumulating and later averaging the activities of the final neuron layer for samples of chosen class. As the patterns remain similar across all labels, we limit presented analyzes to samples with label 1, representing digit 1 in the MNIST dataset. We observe the accumulated activity patterns for different values of *T* to assess how fast the network is able to achieve correct prediction, that is, express the highest spiking activity on the output neuron whose index correspond to the target class.

### 3.9 Stability under noise perturbation

We evaluate the effect of input perturbations on the internal dynamics and robustness of SNNs using MNIST, EventMNIST, UCF11, and CIFAR-10 datasets. After training, each model performs inference on the corresponding test set. For every correctly classified sample, we record the layer-wise spiking activity and then progressively degrade the input by randomly permuting frames in the spike sequence. After each perturbation, the modified sample is re-evaluated. This process continues until the prediction flips from correct to incorrect, at which point we store both the clean and perturbed activities. The procedure is repeated until 2000 correct–incorrect activity pairs are collected or the test dataset is exhausted. The algorithm is outlined in Algorithm 2.

Algorithm 2 **The algorithm showing the procedure of recording layer activities under noise performance.**

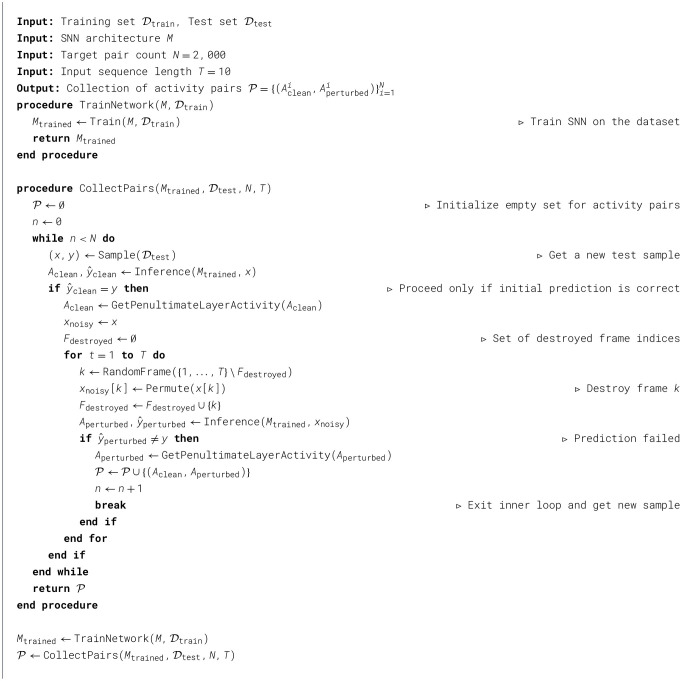

We first performed an exploratory analysis of metrics derived from layer-wise spiking activity under clean and perturbed inputs. Pairwise Pearson correlations between neuronal spike trains exhibited systematic signatures of degradation: as input noise increased, average correlations rose, and strong positive coefficients became more frequent, particularly in deeper layers. These observations motivated us to restrict subsequent analysis to the final spiking layer, where the effects were most pronounced.

For each model, we computed spike-train correlation matrices for all correctly classified samples and averaged them separately for clean and perturbed conditions. From the resulting distributions of correlation coefficients, we extracted the following summary statistics: mean, median, standard deviation, skewness, kurtosis, 99th percentile, and the counts of coefficients exceeding 0.75 and 0.9. Together, these descriptors compactly characterize noise-induced shifts in the correlation structure.

To assess the discriminative value of these descriptors, we constructed a feature dataset in which each sample was represented by the above correlation statistics and labeled by its condition (clean vs. perturbed). We then quantified the dependence between individual features and the label using mutual information and trained an XGBoost classifier to capture higher-order, multivariate relationships. Analysis of the resulting feature-importance profiles reveals which aspects of the correlation structure are most predictive of noise-induced degradation.

## 4 Results

### 4.1 Empirical manifold mapping across architectures

**Figure 2:**
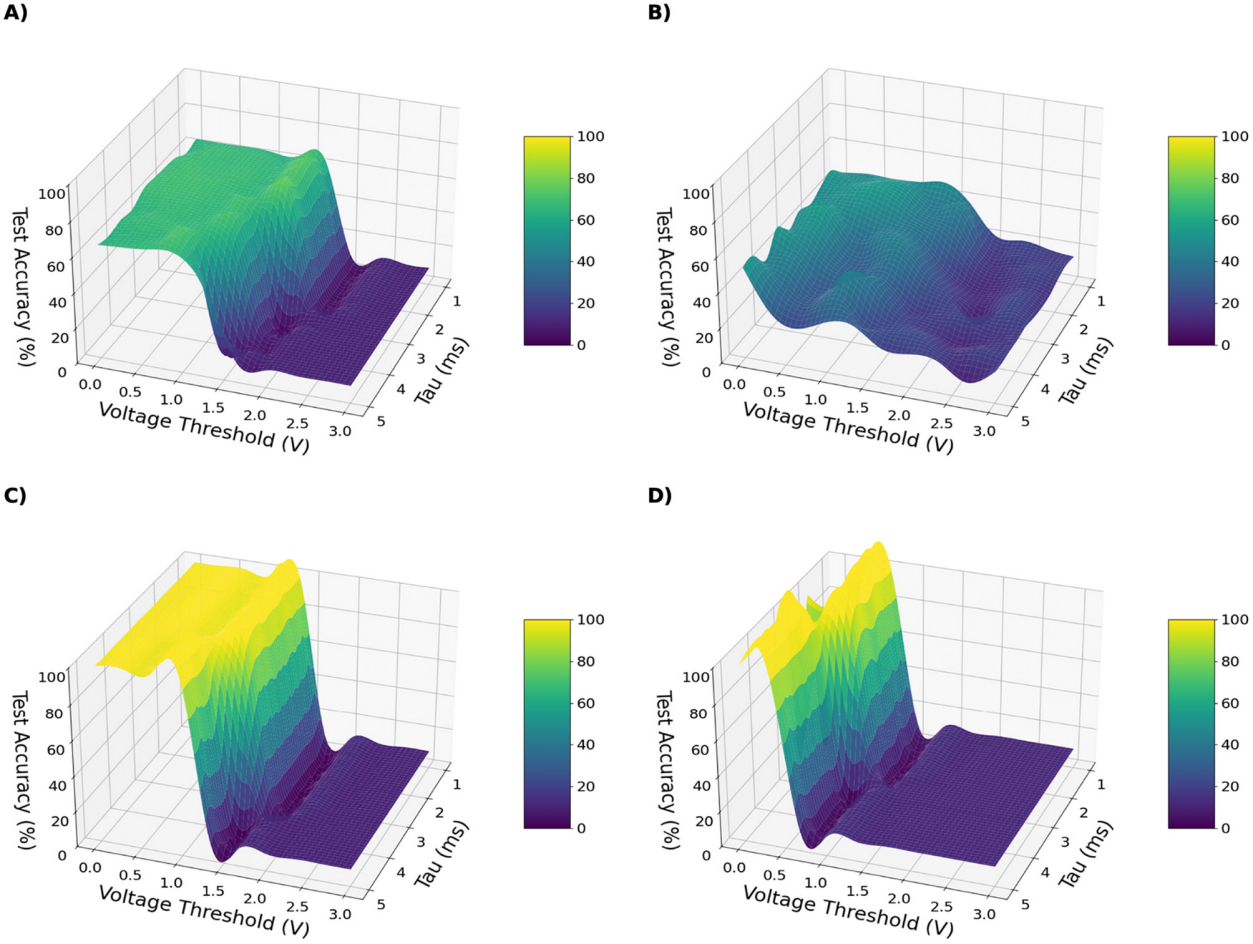
Test accuracy of model-dataset pairs: ConvSNN trained on CIFAR-10 **(A)**, SpikingResnet18 trained on UCF11 **(B)**, ConvSNN trained on MNIST **(C)**, SpikingVGG11 trained on MNIST **(D)** as a function of neuron membrane threshold and decay constant τ.

**Figure 3:**
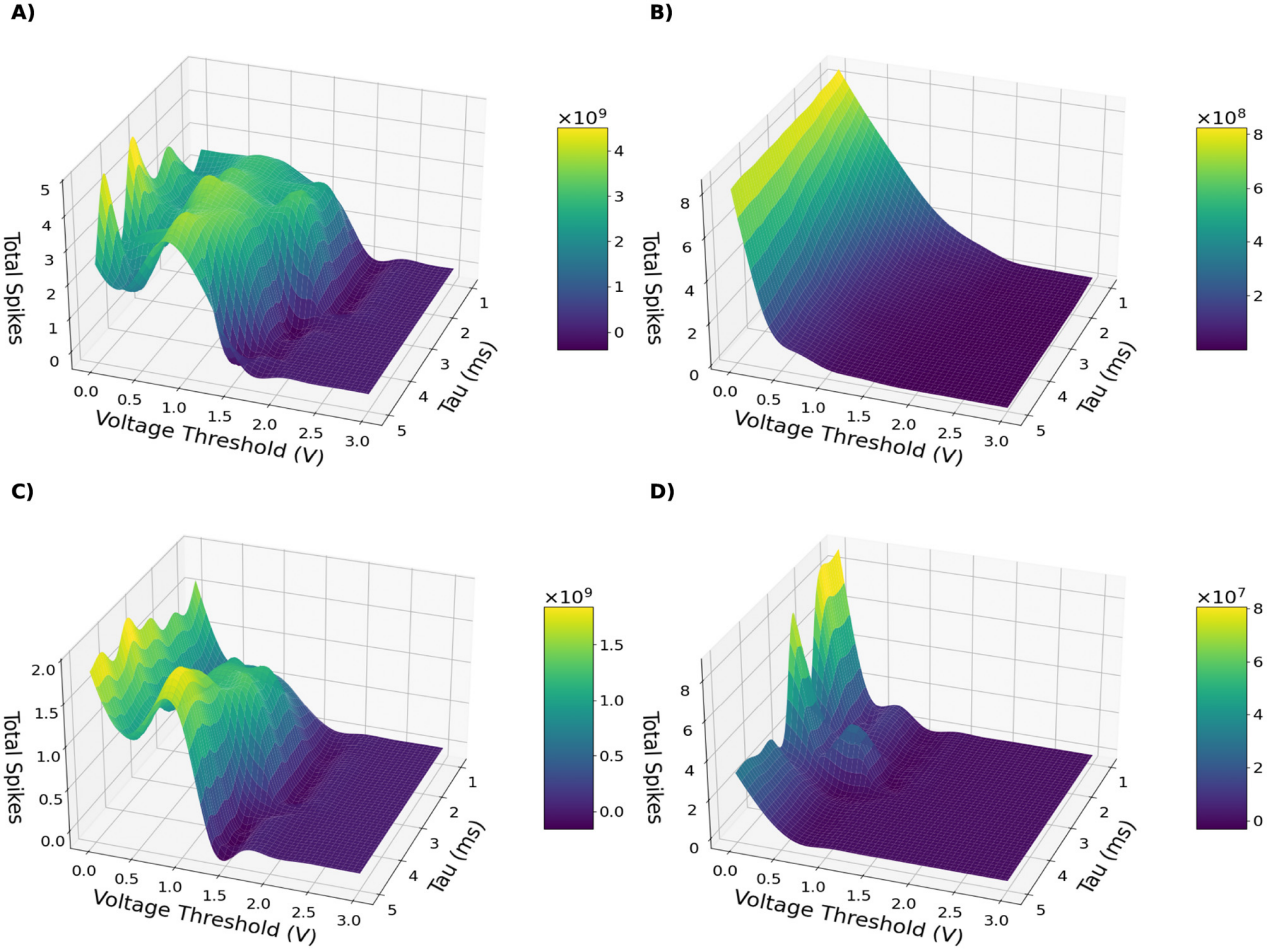
Total number of spikes fired by the network during inference for different model-dataset pairs: ConvSNN trained on CIFAR-10 **(A)**, SpikingResnet18 trained on UCF11 **(B)**, ConvSNN trained on MNIST **(C)**, SpikingVGG11 trained on MNIST **(D)** as a function of neuron membrane threshold and decay constant τ.

Across all datasets and architectures, the resulting landscapes Figures 2, 3 reveal a robust and interpretable structure. For clarity, we show only chosen model-dataset pairs, as they reliably present the described patterns. Remaining plots can be found in Supplementary material.

A characteristic band-shaped region emerges in which networks maintain non-degenerate spiking activity and achieve high accuracy. Outside this region, performance deteriorates in predictable ways:

- **Silent regime:** low τ_*m*_ combined with high *V*_th_ produces vanishing activity and loss of information propagation.
- **Saturated regime:** high τ_*m*_ together with low *V*_th_ results in near-tonic firing and inflated energy cost.

Importantly, within the balanced region many hyperparameter settings achieve near-maximal accuracy while producing substantially fewer spikes, demonstrating that high performance does not require higher firing rates when parameters are appropriately chosen.

A notable pattern appears in the SpikingResnet18 trained on UCF11 dataset (Figures 2B, 3B). The accuracy landscape exhibits a different structure than the smooth manifolds observed in simpler configurations, with performance concentrated around localized maxima. While increasing the voltage threshold rapidly suppresses spiking activity, accuracy degrades more gradually, indicating that neuron hyperparameters primarily regulate firing efficiency rather than accuracy itself. This is advantageous, as similar accuracy can be maintained at substantially reduced spiking activity. However, we note that the accuracy levels reached by the network were not high, with the best models performing at the level of accuracy ∽40%. We did not further tune the training scheme, as this was not the primary objective of this experiment.

Overall, these observations indicate that τ_*m*_ and *V*_th_ serve as primary control parameters governing activity, performance, and energy usage in SNNs.

### 4.2 Mapping the operational manifold

**Figure 4:**
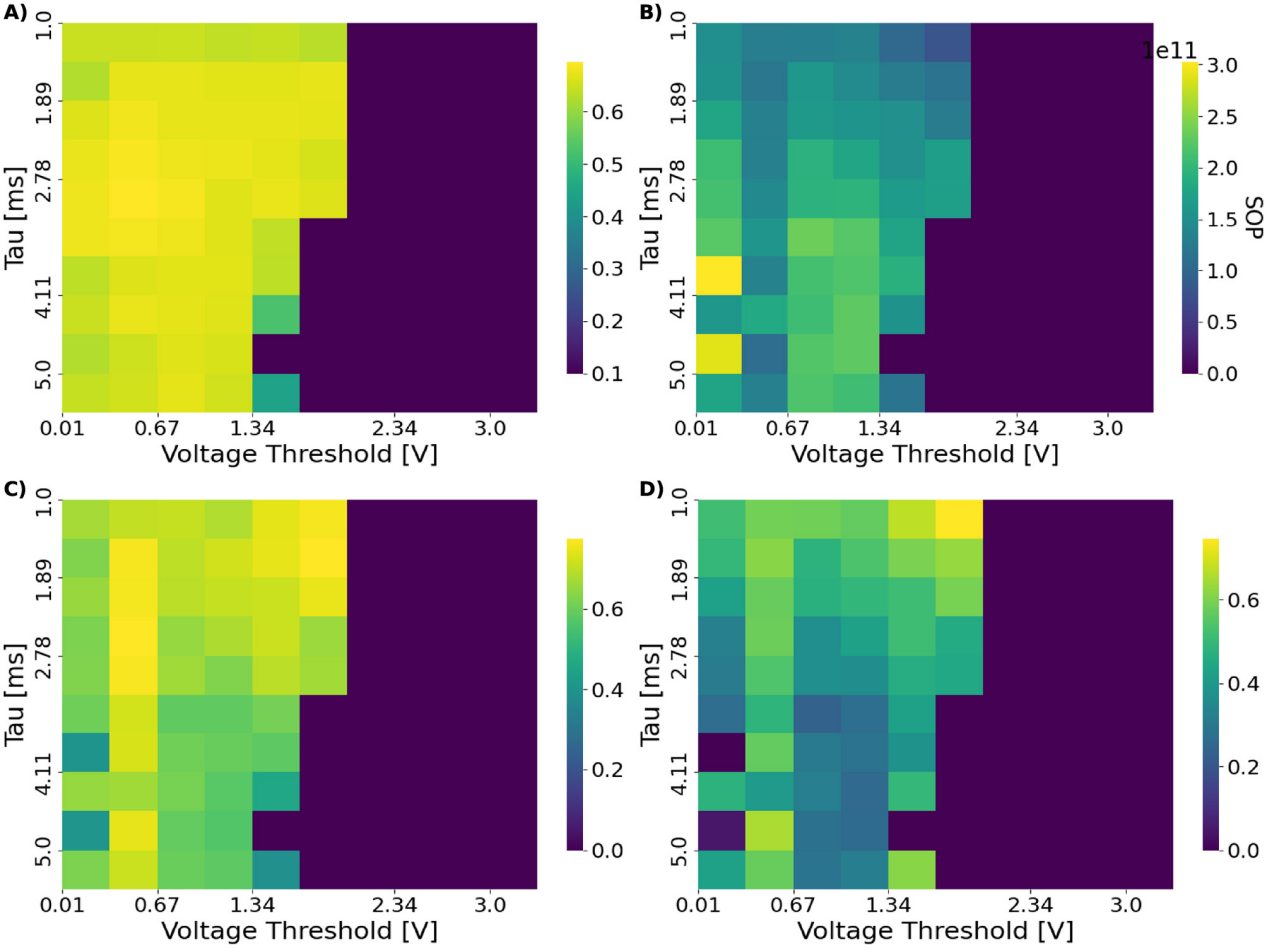
Heatmaps over (τ_*m*_, *V*_th_) showing **(A)** test accuracy, **(B)** SOP on the test set and **(C, D)** the efficiency metrics BES (λ = 0.5) and EAS (β = 5) for the ConvSNN trained on CIFAR-10. The operational manifold M emerges as a contiguous region separating silent from saturated activity regimes. The BES metric enables selecting hyperparameter configurations that balance accuracy with energy efficiency rather than optimizing either quantity in isolation, revealing how different areas of the (τ_*m*_, *V*_th_) space become preferable depending on the desired trade-off.

**Figure 5:**
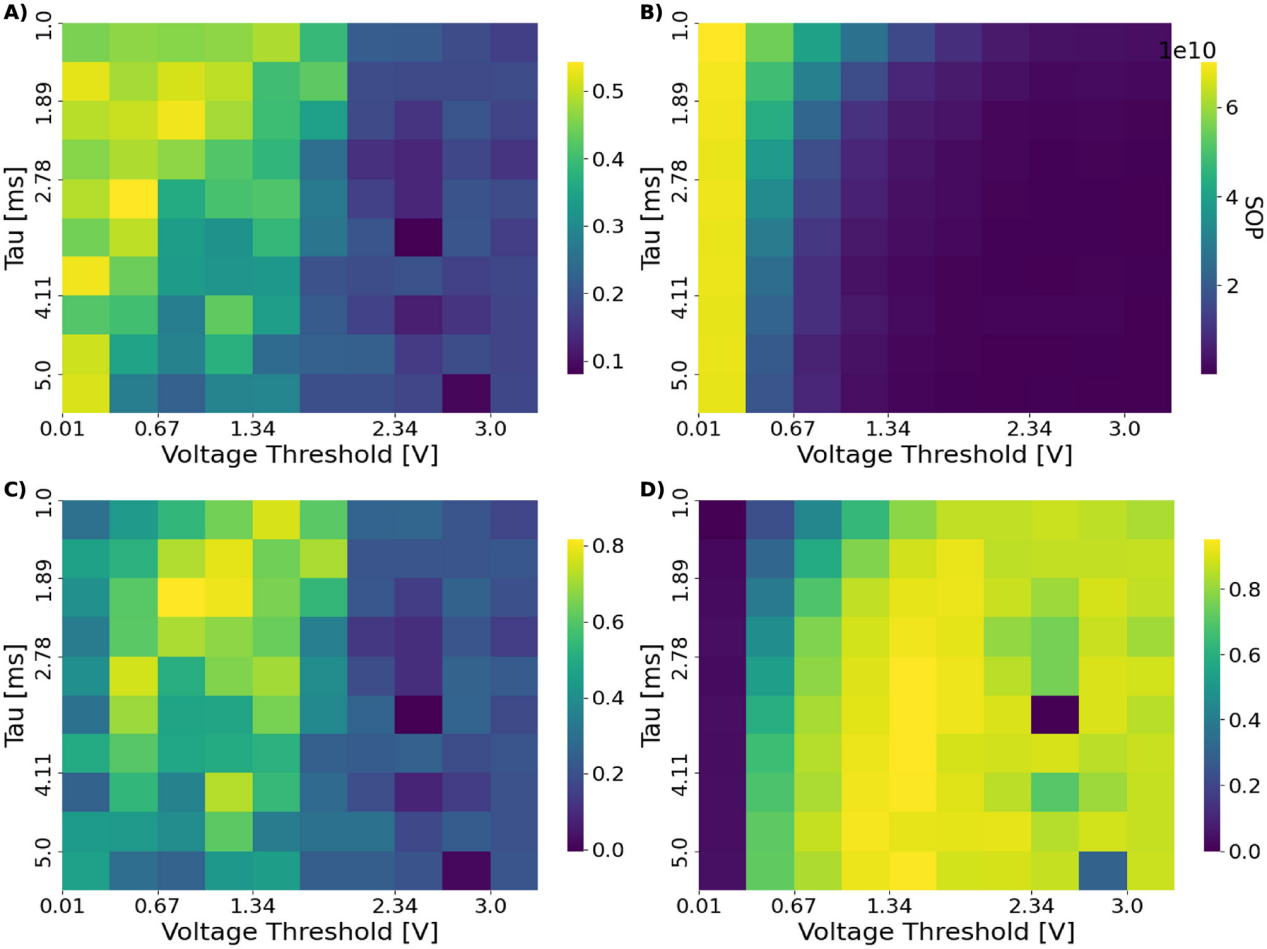
Heatmaps over (τ_*m*_, *V*_th_) showing **(A)** test accuracy, **(B)** SOP during inference on the test set, **(C, D)** the efficiency metrics BES (λ = 0.5) and EAS (β = 5) for the SpikingResnet18 model trained on UCF11. The plots reveal the structure of the operational manifold, with silent and saturated regimes framing a contiguous band of balanced activity. Because the energy proxy is tightly correlated with total spike count, low-SOP regions coincide with sparsely firing configurations. In contrast, the BES score highlights hyperparameter combinations that balance accuracy and efficiency, rather than optimizing either in isolation. Notably, the EAS map exhibits a clearly defined optimal point, reflecting its preference for high-accuracy configurations even at moderate energy cost.

To characterize accuracy–efficiency interactions across the (τ_*m*_, *V*_th_) space, we evaluated two composite metrics, BES_λ_ and EAS_β_, which combine normalized accuracy with synaptic operation cost. Across all models, these metrics reveal a contiguous, diagonally oriented region in which networks maintain balanced spiking activity, achieve high accuracy, and operate at reduced energy cost. This region defines the operational manifold M and is clearly visible in both the ConvSNN trained on CIFAR-10 results, shown in Figure 4, and the SpikingResnet18 trained on UCF11 results, shown in Figure 5. Both figures include accuracy, spike-count, SOP, EAS, and BES maps, making the manifold boundaries explicit across multiple complementary metrics. Once again, we visualize results only for chosen dataset-architecture pairs for clarity, with the remaining plots found in Supplementary material.

The transitions defining M follow the same pattern observed in the activity regimes of Section 4.1. When firing rates are too low, the network fails to propagate meaningful information and accuracy collapses; when firing rates become excessively high, energy cost increases sharply and selectivity deteriorates. Within the manifold, however, accuracy remains near its peak while SOP is substantially reduced. The two composite metrics emphasize different aspects of this behavior: EAS_β_ highlights accuracy-preserving, low-energy configurations, whereas BES_λ_ identifies broader regions that achieve stable accuracy with moderate energy expenditure.

While the existence of an operational manifold is consistent across settings (Figures 4, 5), its scope depends on both architecture and task complexity. SpikingResnet18 exhibits a wider and more diverse manifold than SpikingVGG11, indicating greater robustness to neuron hyperparameter variations.

### 4.3 Reset vs. carry

**Figure 6:**
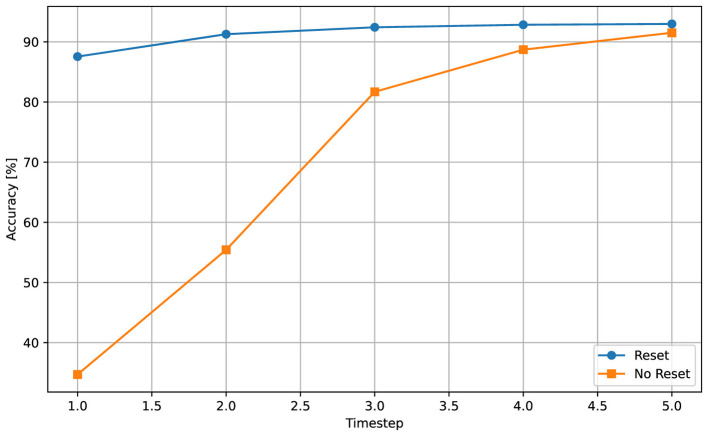
Impact of reset or carry policies on test accuracy as a function of timesteps *T*. In the reset scenario, network is able to achieve near-maximal performance starting from the first sample. Without it, similar accuracy level is achieved after assessing the full sample.

We evaluated how the handling of membrane potentials between consecutive inputs influences SNN inference by comparing two policies: *reset*, where all membrane states are reinitialized before each new sample, and *carry*, where the final membrane potential is preserved across samples. Figure 6 shows the test accuracy as a function of the number of time steps *T* for both strategies during inference.

The reset policy consistently yields higher accuracy, especially for small values of *T*. When membrane states are carried across samples, residual activity from the previous input interferes with the early integration of the next input, reducing accuracy. As *T* increases, this interference is gradually overwritten, and the gap between the two policies narrows.

The reset policy, in contrast, exhibits stable and near-saturated accuracy already for moderate values of *T* (typically *T*≈3–4), indicating that explicit state reinitialization is well suited for static, independent inputs.

**Figure 7:**
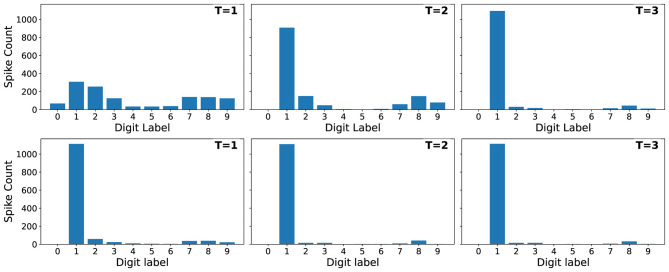
Spike counts in the output layer for the first three timesteps when classifying samples of label 1 under *carry* **(top row)** and *reset* **(bottom row)** policies. Without resetting, the first timestep exhibits strong residual activation from the previous input, producing unintended spike bursts and delaying convergence to the correct output. With reset, the network immediately produces a clean class-specific response. This illustrates how membrane-state carryover induces cross-sample interference during inference.

To further illustrate the source of the accuracy gap between reset and carry modes, Figure 7 visualizes the spike counts of the output layer when classifying samples of label 1 (Digit 1). We limit the visualization to shown only results for inference sample length *T* ∈ {1, 2, 3}, as we observed that after for values of *T*>3 the network already stabilizes in the correct prediction, with little change in spiking activities of output neruons. In the carry mode (Figure 7 top row), the activity during the first timestep is dominated by residual membrane potentials from the previous input, producing large unintended spike bursts on neurons unrelated to the current class. The network requires several timesteps to dissipate this residual activity before converging toward the correct output pattern. In contrast, under the reset policy (Figure 7 bottom row), the spiking pattern is aligned with the correct class from the very first timestep. This example highlights that, for inputs, carrying membrane state introduces cross-sample interference, which primarily affects short inference windows and explains the reduced accuracy observed at low *T*.

### 4.4 Stability under noise perturbation

To assess stability of trained SNNs, we examined how input perturbations alter internal spike-train correlations across architectures and datasets. Within the operational manifold M, networks exhibit sparse, decorrelated activity patterns consistent with balanced excitation and inhibition. When driven outside this manifold—through either noise injection or unfavorable hyperparameter settings—neuronal firing becomes progressively synchronized, producing dense blocks of high pairwise correlation coefficients. The observations and conclusions across all architecture and dataset combinations remain similar, especially visible in the distribution statistics. We therefore show and describe results for the chosen dataset-architecture combination as an example, for simple and deep network respectively. The remaining results are shown in Supplementary material.

**Figure 8:**
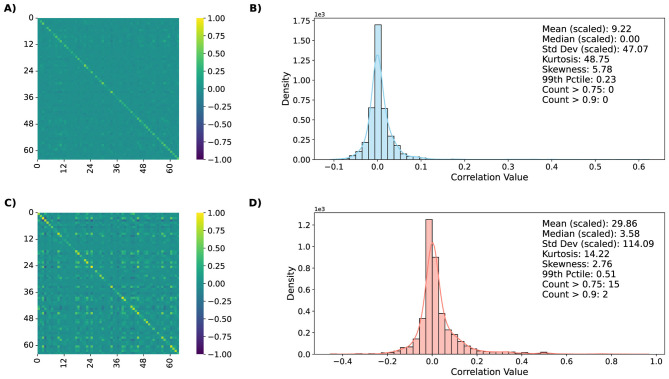
Average spike-train correlation matrices and their distributions for clean **(A, B)** and noisy **(C, D)** inputs for ConvSNN trained on UCF11. Within the operational manifold, correlations remain sparse and distributed. Outside the manifold, noise induces synchronization and clustering, indicating over-excitation and loss of representational diversity.

**Figure 9:**
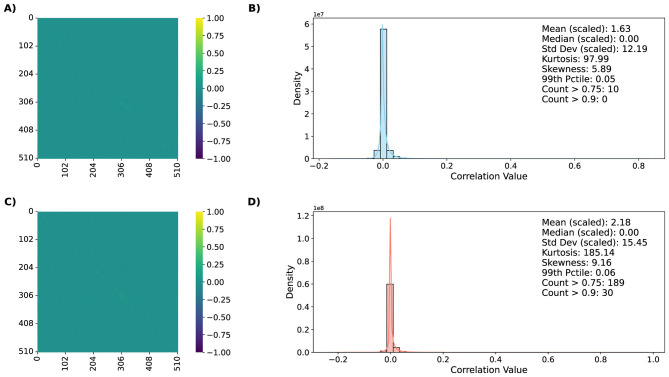
Average spike-train correlation matrices and their distributions for clean **(A, B)** and noisy **(C, D)** inputs for SpikingResnet18 trained on UCF11. Due to higher number of output neurons, correlation matrices do not display the clear patterns, yet the changes in distributions of correlation values are still visible.

Figures 8, 9 visualize these effects for ConvSNN and SpikingResnet18 trained on UCF11 dataset, respectively. For clean, in-distribution conditions, correlation matrices are dominated by weak off-diagonal elements, reflecting distributed, task-specific representations. As noise increases and the system approaches the manifold boundary, correlations strengthen and spatially cluster, indicating the onset of population-level synchronization. This transition corresponds to degraded information flow and energy inefficiency, mirroring the accuracy collapse and SOP inflation observed in earlier sections. Analysis of correlation distributions shows that with increasing input noise, the number of extreme values increases, forming a long tail. An increase in the mean and standard deviation values is also visible. Together, these shifts capture the progression from sparse, asynchronous activity to strongly coupled dynamics. Such patterns provide interpretable early-warning indicators of performance drift and may serve as lightweight on-device health metrics.

**Figure 10:**
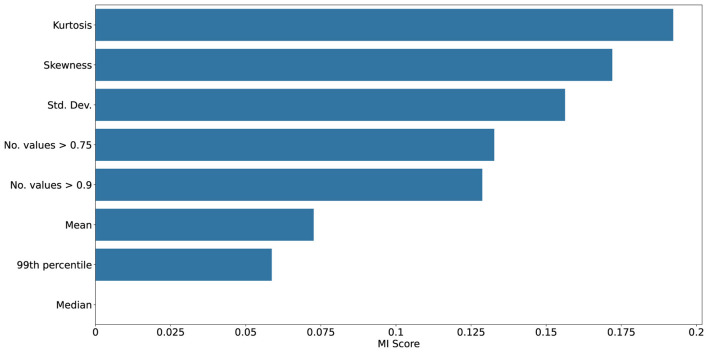
Mutual information scores between given statistic and clean vs. noisy input conditions collected from ConvSNN trained on the UCF11 dataset. Kurtosis, skewness, and number of high correlation values shows to be the most reliable predictor, underscoring their value as interpretable indicators of robustness and drift.

**Figure 11:**
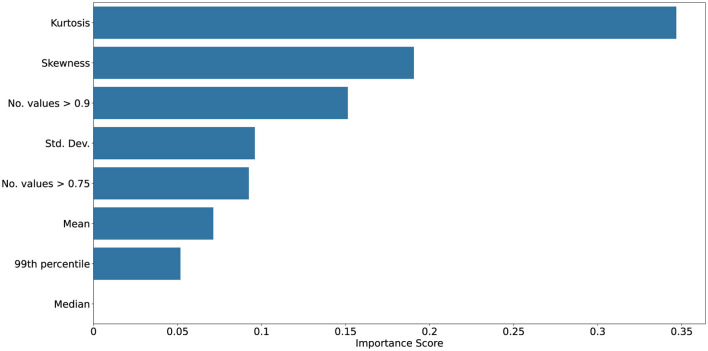
Feature importances from an XGBoost classifier distinguishing clean vs. noisy input conditions collected from ConvSNN trained on the UCF11 dataset. Kurtosis, skewness, and number of high correlation values shows to be the most reliable predictor, underscoring their value as interpretable indicators of robustness and drift.

**Figure 12:**
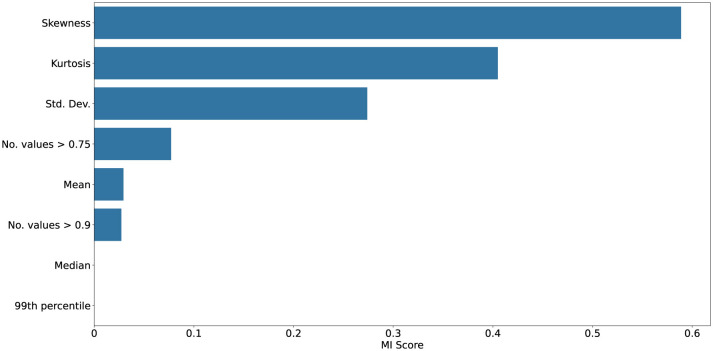
Mutual information scores between given statistic and clean vs. noisy input conditions collected from SpikingResnet18 trained on the UCF11 dataset. Kurtosis, skewness, and number of high correlation values shows to be the most reliable predictor, underscoring their value as interpretable indicators of robustness and drift.

**Figure 13:**
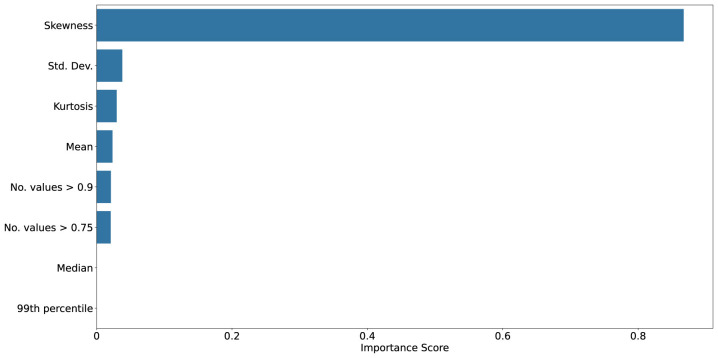
Feature importances from an XGBoost classifier distinguishing clean vs. noisy input conditions collected from SpikingResnet18 trained on the UCF11 dataset. In this case, strong dominance of higher order statistics, particularly skewness, can be seen as the most distinctive feature for distinguishing noisy samples.

Figures 10–13 show mutual information and feature importances learned by an XGBoost classifier trained to discriminate clean vs. noisy inputs. Higher-order statistics and number of extreme values contribute the most to separability, confirming that deviations in correlation structure encode diagnostic information about noise exposure and functional degradation. Detailed results of achieved XGBoost accuracies can be found in the Supplementary material.

## 5 Discussion

The operational manifold provides a practical recipe for selecting energy-efficient and robust operating points for SNNs. Hyperparameters should first be placed within the balanced band of the manifold, where population activity remains between silent and saturated regimes. Configurations in this region preserve task accuracy while substantially reducing spike counts and, consequently, synaptic operation cost.

Once a balanced configuration has been identified, the simulation horizon (number of timesteps *T*) should be chosen no larger than required for accuracy to converge. Increasing *T* beyond this point primarily inflates latency and energy consumption, with diminishing returns in performance. For standard benchmarks such as MNIST or CIFAR-10, a *reset* policy is typically preferable, as it suppresses cross-sample interference and matches the isolated-sample conditions under which the manifold was characterized.

In contrast, real-world neuromorphic sensing systems operate on continuous, temporally correlated streams. In these settings, hard resets are neither natural nor desirable, because membrane potentials encode short-term temporal context that can improve stability and expressivity. Here, a *carry* policy, possibly combined with conditional or partial resets, is more suitable and should be tuned in conjunction with the manifold to maintain balanced activity over time.

We recommend monitoring higher-order statistics of spike-train correlations—particularly skewness and kurtosis—as lightweight health indicators. Systematic drifts in these descriptors signal emerging deviations from the balanced regime and can be used to trigger adaptive retuning of hyperparameters or reset policies before severe degradation occurs.

### 5.1 Impact of higher-dimensional neuron parameters on manifold topology

The operational manifold can be understood as the contiguous region in the full neuron hyperparameter space where the network's activity remains balanced (neither quiescent nor saturated) and performance is high. In our study we focused on the two most influential parameters—membrane time constant (τ_*m*_) and firing threshold (*V*_th_)—for tractability, but the concept generalizes to additional dimensions. If we include other neuron parameters (e.g., refractory period, resting potential, synaptic scaling) or the simulation duration *T*, the operational manifold becomes a higher-dimensional surface (or hyper-volume) within that augmented parameter space. Intuitively, each extra parameter provides another “degree of freedom” that can influence firing activity, so the manifold's topology may bend or stretch along new axes. For example, allowing a longer integration window *T* can compensate for slower neuron dynamics (larger τ_*m*_), potentially extending the manifold along the τ_*m*_ dimension by keeping the network active and accurate even with more sluggish neurons. Similarly, introducing a refractory period would create an additional trade-off: extremely short refractory intervals could lead to rapid, potentially saturating spiking unless counterbalanced by a higher threshold or shorter τ_*m*_, whereas very long refractory periods might suppress spiking (risking silence) unless offset by more excitable settings (lower *V*_th_, etc.). In essence, each parameter contributes its own failure modes at extremes (e.g., too excitable or not excitable enough), but within moderate ranges these parameters can co-operate to preserve a homeostatic balance. Thus, in higher dimensions the operational manifold is expected to remain a contiguous “sweet spot” surface, though its shape may curve to reflect compensatory interactions between parameters (e.g., increasing one parameter might require decreasing another to stay in balance). This perspective aligns with the notion of a broad homeostatic regime observed in biology (akin to Griffiths phases—extended critical-like regions in brain networks). Incorporating additional neuron-level parameters would extend the manifold rather than fundamentally alter its existence—the network still occupies a topological region of stable operation, bounded on various sides by silent or epileptic (over-active) regimes, but now in a higher-dimensional space.

### 5.2 Hardware implications

Balanced regimes maximize energy savings on event-driven substrates by minimizing SOPs without triggering accuracy collapse. Within the operational manifold, average firing rates are low enough that synaptic operation counts (and thus dynamic compute energy) are significantly reduced, yet high enough to preserve representational capacity. In this regime, the primary cost on neuromorphic ASICs and other event-driven accelerators shifts from arithmetic to memory: SRAM residency of neuron state (membrane potentials, refractory flags, and adaptation variables) and spike queues becomes the dominant bottleneck.

Practically, this implies that once an SNN has been tuned into the balanced band, further gains from reducing spike rates alone are limited by the fixed cost of storing and updating network state. Each active neuron still requires per-timestep access to its local state, and each spike must be buffered and routed through on-chip queues, even when overall activity is sparse. As a result, the achievable energy–efficiency and throughput on hardware are increasingly governed by on-chip SRAM capacity, access patterns, and interconnect organization, rather than by the peak number of synaptic operators.

These observations suggest a concrete co-design guideline: hyperparameters should be used to place the network inside the operational manifold to control SOPs, while hardware-aware model design (e.g., constrained fan-out, structured sparsity, state compression, and careful partitioning across cores) should target the SRAM footprint and spike-buffer pressure. In other words, balanced SNNs are compute-light but memory-bound, and practical neuromorphic deployments must treat local memory hierarchy and spike-queue management as first-class design constraints.

### 5.3 Limitations

The operational manifold is estimated empirically and therefore its absolute location and width can be dataset-dependent. Because the manifold is defined by simultaneous constraints on firing activity and task performance, transferring an operating point from one dataset to another (with different input statistics and task difficulty) may shift the balanced region. This effect is visible when comparing manifold heatmaps across datasets where the high-performing/balanced band changes its extent and placement (e.g., CIFAR-10 vs. MNIST across comparable architectures). Consequently, for substantial distribution shifts, the manifold should be at least re-validated, and in many cases re-estimated, for the new data regime.

However, the qualitative ordering in (τ_*m*_, *V*_*th*_) space is largely preserved: across architectures and datasets we consistently observe silent regimes at low τ_*m*_ and high *V*_*th*_, saturated regimes at high τ_*m*_ and low *V*_*th*_, and a diagonally oriented balanced band separating these extremes. This structure implies that transfer does not require a search from scratch in an unconstrained space rather, a manifold estimated on one dataset can serve as a useful prior region for another dataset, enabling a coarse-to-fine refinement procedure (local scan) using the same firing-rate and accuracy criteria.

A second limitation concerns the training procedure. Our analysis is based on networks trained with a particular choice of surrogate gradient and associated hyperparameters. Different surrogate functions, slope parameters, or gradient-clipping strategies can materially affect gradient flow, convergence, and the resulting firing-rate distribution. As a consequence, the quantitative manifold boundaries reported here should not be interpreted as invariant properties of a given dataset, but rather as properties of a specific training recipe. While we expect the qualitative picture (existence of silent, balanced, and saturated regimes) to be robust, the precise transition points and their sensitivity to noise or perturbations may differ under alternative surrogate designs.

Consequently, our energy estimates rely on proxy models that approximate cost in terms of synaptic operations and simple memory-access assumptions, in the absence of chip-specific measurements. These models ignore many hardware-dependent factors, including leakage, control overheads, routing and spike-queue implementation details, clock gating, and process variations. As a result, the reported savings should be interpreted as relative trends rather than absolute figures. Accurate assessment of energy–latency trade-offs in practical deployments will require validation with silicon measurements or detailed, architecture-specific simulators, as well as extending the analysis to a broader set of neuromorphic platforms.

### 5.4 Biological and artificial SNNs: emergent similarities and cross-disciplinarity

Our analysis indicates that even relatively simple artificial SNNs exhibit several qualitative phenomena that parallel observations in biological neuronal circuits. In particular, when driven by noise or operated outside the balanced regime of the operational manifold, we observe characteristic changes in firing statistics and spike-train correlations that resemble transitions between healthy, irregular spiking and more pathological regimes such as quiescence or runaway synchrony. In the balanced band, population activity remains sparse yet responsive, with heterogeneous spike patterns and moderate correlations. As noise levels increase or operating points shift toward saturation, correlations rise, higher-order statistics (e.g., skewness, kurtosis) become distorted, and the network's representational capacity degrades. This mirrors the broader neurophysiological intuition that cortical networks must maintain a form of functional “homeostasis” to support robust computation in the presence of stochastic inputs (Turrigiano, 2012; Marder and Goaillard, 2006; Vogels et al., 2011; Denève and Machens, 2016; Chen et al., 2022).

Importantly, our results do not prescribe a specific homeostatic mechanism (e.g., synaptic scaling or intrinsic plasticity). Rather, the manifold-based perspective suggests that successful SNN operation occupies a restricted region of parameter space where effective self-regulation emerges at the population level: activity neither dies out nor explodes, and noise does not catastrophically disrupt internal representations. This resonates with concepts such as excitation–inhibition balance, criticality, and dynamic range maximization in biological networks, and highlights how tools from machine learning (operational manifolds, correlation-structure analysis) and systems neuroscience can jointly illuminate the conditions under which spiking computation is stable and efficient.

A second point of contact with biology concerns temporal continuity. Whereas conventional ANNs are typically deployed in a strictly discrete mode—processing isolated input frames and producing independent predictions—SNNs are inherently continuous-time dynamical systems. Our experiments on *reset* vs. *carry* policies for membrane potentials reinforce this view: enforcing a hard reset between inputs approximates the ANN-style i.i.d. setting and simplifies analysis, but it is at odds with how real sensory systems operate. Biological neurons do not globally reset between successive glimpses of the world; instead, they maintain and update internal state as streams of stimuli arrive. Artificial SNNs show analogous behavior: when membrane state is carried across inputs, the network effectively “lives” in time, integrating information over longer horizons and exploiting short-term context, particularly for event-based or sequential data.

These parallels have concrete implications for neuromorphic hardware and for cross-disciplinary research. From a hardware perspective, the continuous, stateful operation of SNNs suggests that architectures prioritizing persistent, low-leak storage of neuronal state and efficient event routing—potentially involving mixed-signal or analog elements—may be more natural than purely clocked, frame-based digital designs. From a scientific perspective, the fact that artificial SNNs exhibit homeostasis-like operating regimes, noise-induced transitions, and temporally extended dynamics creates an opportunity for a tighter dialogue between computational neuroscience, machine learning, and circuit design. Operational manifolds, as introduced here, offer one possible bridge: they provide a quantitative language for comparing biological and artificial spiking systems, and for guiding the co-design of learning rules, network architectures, and neuromorphic substrates that exploit, rather than fight against, the intrinsically dynamical nature of spiking computation.

## 6 Conclusion

In this work, we introduced the notion of an *operational manifold* for SNNs, defined over neuron-level hyperparameters such as membrane time constant and firing threshold. By systematically mapping this space across architectures and datasets, we demonstrated that SNNs admit a contiguous band of configurations that simultaneously avoid silence and saturation, maintain high task accuracy, and substantially reduce spike counts and synaptic operation cost. This balanced regime provides a principled target for tuning neuron excitability, replacing *ad-hoc* hyperparameter search with an interpretable operating region.

Building on this perspective, we proposed composite efficiency metrics (BES and EAS) that jointly account for normalized accuracy and SOP-based energy proxies. These scores expose accuracy–energy frontiers within the manifold and allow practitioners to select operating points according to deployment priorities (e.g., favoring peak accuracy vs. aggressive energy savings). Our experiments show that many configurations inside the manifold achieve near-maximal accuracy at a fraction of the spike cost, underscoring that high performance does not require high firing rates when hyperparameters are appropriately matched to the task.

We further analyzed the temporal and robustness aspects of SNN deployment. For inference-time state handling, we compared *reset* and *carry* policies for membrane potentials and quantified their impact on accuracy and latency. On static, i.i.d. datasets, hard reset leads to more stable and higher accuracy for short inference windows, whereas carrying membrane state becomes advantageous for temporally correlated streams. In parallel, we introduced correlation-based diagnostics that track how spike-train statistics (including higher-order descriptors such as skewness and kurtosis) evolve under input perturbations and manifold violations. These measures provide lightweight, label-free indicators of emerging synchronization, loss of representational diversity, and concept drift.

Taken together, our findings position neuron hyperparameters as practical control knobs for shaping the dynamical regime of SNNs and for co-optimizing accuracy, energy, and robustness. The operational manifold serves both as a conceptual lens—linking biophysical parameters, population activity, and task performance—and as a concrete design tool for neuromorphic deployment. Future work will extend this framework to richer neuron models and on-chip measurements, explore adaptive mechanisms that keep networks within the balanced regime during lifelong operation, and tighten the connection to biological homeostatic principles and hardware-level co-design.

## Data Availability

The original contributions presented in the study are included in the article/Supplementary material, further inquiries can be directed to the corresponding author.

## References

[B1] Ayers J. G. Ramanan B. A. Khan M. A. (2025). Detecting concept drift in neural networks using chi-squared goodness of fit testing. arXiv preprint arXiv:2505.04318.

[B2] Baier L. Schlör T. Schöffer J. Kühl N. (2021). “Detecting concept drift with neural network model uncertainty,” in Hawaii International Conference on System Sciences.

[B3] Beggs J. M. Plenz D. (2003). Neuronal avalanches in neocortical circuits. J. Neurosci. 23, 11167–11177. doi: 10.1523/JNEUROSCI.23-35-11167.2003 14657176 PMC6741045

[B4] Bodyanskiy Y. V. Savenkov D. V. (2024). Ensemble of simple Spiking Neural Networks as a concept drift detector. Radio Electronics, Computer Science, Control. doi: 10.15588/1607-3274-2024-4-8

[B5] Chen L. Li X. Tjia M. Thapliyal S. (2022). Homeostatic plasticity and excitation-inhibition balance: The good, the bad, and the ugly. Curr. Opin. Neurobiol. 75:102553. doi: 10.1016/j.conb.2022.102553 35594578 PMC9477500

[B6] Christensen D. V. (2022). 2022 roadmap on neuromorphic computing and engineering. Neurom. Comput. Eng. 2:022501.

[B7] Dean I. Robinson B. L. Harper N. S. McAlpine D. (2008). Rapid neural adaptation to sound level statistics. J. Neurosci. 28, 6430–6438. doi: 10.1523/JNEUROSCI.0470-08.2008 18562614 PMC6670892

[B8] Denéve S. Machens C. K. (2016). Efficient codes and balanced networks. Nat. Neurosci. 19, 375–382. doi: 10.1038/nn.4243 26906504

[B9] Ding J. Zhang J. Huang T. Liu J. K. Yu Z. (2025). Assisting training of deep spiking neural networks with parameter initialization. IEEE Trans. Neural Netw. Learn. Syst. 36, 15015–15028. doi: 10.1109/TNNLS.2025.3547774 40168228

[B10] El-Allami R. Marchisio A. Shafique M. Alouani I. (2021). “Securing deep spiking neural networks against adversarial attacks through inherent structural parameters,” in 2021 Design, Automation & Test in Europe Conference & Exhibition (DATE) (IEEE), 774–779. doi: 10.23919/DATE51398.2021.9473981

[B11] Eskikand P. Z. Soto-Breceda A. Cook M. J. Burkitt A. N. Grayden D. B. (2023). Inhibitory stabilized network behaviour in a balanced neural mass model of a cortical column. bioRxiv [Preprint]. doi: 10.1016/j.neunet.2023.07.020 37541162

[B12] Greco S. Vacchetti B. Apiletti D. Cerquitelli T. (2025). Unsupervised concept drift detection from deep learning representations in real-time. IEEE Trans. Knowl. Data Eng. 37, 6232–6245. doi: 10.1109/TKDE.2025.3593123

[B13] Habara T. Sato T. Awano H. (2024). BayesianSpikeFusion: accelerating spiking neural network inference via Bayesian fusion of early prediction. Front. Neurosci. 18:1420119. doi: 10.3389/fnins.2024.1420119 39161650 PMC11330889

[B14] Han B. Srinivasan G. Roy K. (2020). “RMP-SNN: residual membrane potential neuron for enabling deeper high-accuracy and low-latency spiking neural network,” in 2020 IEEE/CVF Conference on Computer Vision and Pattern Recognition (CVPR), 13555–13564. doi: 10.1109/CVPR42600.2020.01357

[B15] Hu J. Man Y. Qiu X. Chou Y. Cai Y. Qiao N. . (2024). “High-performance temporal reversible spiking neural networks with O(L) training memory and O(1) inference cost,” in International Conference on Machine Learning, ICML'24 (JMLR.org).

[B16] Hu L. Lu Y. Feng Y. (2025). Concept drift detection based on deep neural networks and autoencoders. Appl. Sci. 15:3056. doi: 10.3390/app15063056

[B17] Hu Y. Tang H. Pan G. (2023). Spiking deep residual networks. IEEE Trans. Neural Netw. Learn. Syst. 34, 5200–5205. doi: 10.1109/TNNLS.2021.3119238 34723807

[B18] Huynh P. K. Varshika M. L. Paul A. Isik M. Balaji A. Das A. (2022). Implementing spiking neural networks on neuromorphic architectures: a review. arXiv preprint arXiv:2202.08897.

[B19] Kingma D. P. Ba J. (2015). “Adam: a method for stochastic optimization,” in 3rd International Conference on Learning Representations (ICLR 2015) (San Diego, CA, USA).

[B20] Krizhevsky A. (2009). Learning Multiple Layers of Features from Tiny Images. Technical report, University of Toronto.

[B21] LeCun Y. Cortes C. Burges C. J. (2010). MNIST handwritten digit database. AT and T Labs. Available online at: http://yann.lecun.com/exdb/mnist (Accessed October 2, 2025).

[B22] Lu S. Sengupta A. (2024). Deep unsupervised learning using spike-timing-dependent plasticity. Neuromor. Comput. Eng. 4:024004. doi: 10.1088/2634-4386/ad3a95

[B23] Marder E. Goaillard J.-M. (2006). Variability, compensation and homeostasis in neuron and network function. Nat. Rev. Neurosci. 7, 563–574. doi: 10.1038/nrn1949 16791145

[B24] Masquelier T. Thorpe S. (2007). Unsupervised learning of visual features through spike timing dependent plasticity. PLoS Comput. Biol. 3:e31. doi: 10.1371/journal.pcbi.0030031 17305422 PMC1797822

[B25] Moretti P. Muñoz M. A. (2013). Griffiths phases and the stretching of criticality in brain networks. Nat. Commun. 4:2521. doi: 10.1038/ncomms3521 24088740

[B26] Nassar M. R. Scott D. Bhandari A. (2021). Noise correlations for faster and more robust learning. J. Neurosci. 41, 6740–6752. doi: 10.1523/JNEUROSCI.3045-20.2021 34193556 PMC8336712

[B27] Neftci E. O. Mostafa H. Zenke F. (2019). Surrogate gradient learning in spiking neural networks: bringing the power of gradient-based optimization to spiking neural networks. IEEE Signal Process. Mag. 36, 51–63. doi: 10.1109/MSP.2019.2931595

[B28] Niu L.-Y. Wei Y. Liu W.-B. Long J.-Y. Xue T.-H. (2023). Research progress of spiking neural network in image classification: a review. Appl. Intell. 53, 19466–19490. doi: 10.1007/s10489-023-04553-0

[B29] Orchard G. Jayawant A. Cohen G. K. Thakor N. (2015). Converting static image datasets to spiking neuromorphic datasets using saccades. Front. Neurosci. 9:437. doi: 10.3389/fnins.2015.00437 26635513 PMC4644806

[B30] Ozeki H. Finn I. M. Schaffer E. S. Miller K. D. Ferster D. (2009). Inhibitory stabilization of the cortical network underlies visual surround suppression. Neuron 62, 578–592. doi: 10.1016/j.neuron.2009.03.028 19477158 PMC2691725

[B31] Rathi N. Roy K. (2023). DIET-SNN: a low-latency spiking neural network with direct input encoding and leakage and threshold optimization. IEEE Trans. Neural Netw. Learn. Syst. 34, 3174–3182. doi: 10.1109/TNNLS.2021.3111897 34596559

[B32] Reddy K. K. Shah M. (2012). Recognizing 50 human action categories of web videos. Mach. Vis. Appl. 23, 963–987. doi: 10.1007/s00138-012-0450-4

[B33] Roy K. Jaiswal A. Panda P. (2019). Towards spike-based machine intelligence with neuromorphic computing. Nature 575, 607–617. doi: 10.1038/s41586-019-1677-2 31776490

[B34] Sengupta A. Ye Y. Wang R. Liu C. Roy K. (2019). Going deeper in spiking neural networks: Vgg and residual architectures. Front. Neurosci. 13:95. doi: 10.3389/fnins.2019.00095 30899212 PMC6416793

[B35] Serrano-Gotarredona T. Linares-Barranco B. (2015). Poker-DVS and MNIST-DVS. their history, how they were made, and other details. Front. Neurosci. 9:481. doi: 10.3389/fnins.2015.00481 26733794 PMC4686704

[B36] Strack B. Jacobs K. M. Cios K. J. (2013). “Biological restraint on the Izhikevich neuron model essential for seizure modeling,” in 2013 6th International IEEE/EMBS Conference on Neural Engineering (NER), 395–398. doi: 10.1109/NER.2013.6695955 36818466 PMC9937452

[B37] Suárez-Cetrulo A. L. Quintana D. Cervantes A. (2023). A survey on machine learning for recurring concept drifting data streams. Expert Syst. Appl. 213:118934. doi: 10.1016/j.eswa.2022.118934

[B38] Turrigiano G. (2011). Too many cooks? Intrinsic and synaptic homeostatic mechanisms in cortical circuit refinement. Annu. Rev. Neurosci. 34, 89–103. doi: 10.1146/annurev-neuro-060909-153238 21438687

[B39] Turrigiano G. G. (2012). Homeostatic synaptic plasticity: local and global mechanisms for stabilizing neuronal function. Cold Spring Harb. Perspect. Biol. 4:a005736. doi: 10.1101/cshperspect.a005736 22086977 PMC3249629

[B40] Vogels T. P. Sprekeler H. Zenke F. Clopath C. Gerstner W. (2011). Inhibitory plasticity balances excitation and inhibition in sensory pathways and memory networks. Science 334, 1569–1573. doi: 10.1126/science.1211095 22075724

[B41] Wu D. Jin G. Yu H. Yi X. Huang X. (2025). Optimizing event-driven spiking neural network with regularization and cutoff. Front. Neurosci. 19:1522788. doi: 10.3389/fnins.2025.1522788 40046439 PMC11880274

[B42] Yin B. Corradi F. (2025). “Never reset again: a mathematical framework for continual inference in recurrent neural networks,” in 2025 Neuro Inspired Computational Elements (NICE), 1–9. doi: 10.1109/NICE65350.2025.11065065

[B43] Zhang E. (2025). Revisiting reset mechanisms in spiking neural networks for sequential modeling: specialized discretization for binary activated RNN. arXiv preprint arXiv:2504.17751.

[B44] Zhang S.-Q. Zhang Z.-Y. Zhou Z.-H. (2021). Bifurcation spiking neural network. J. Mach. Learn. Res. 22, 1–21.

