# Supplementary material for: Operational manifolds in spiking neural networks
Source: Front Neurosci. 2026 Feb 18;20:1755119. doi: 10.3389/fnins.2026.1755119 (PMC12956522; doi:10.3389/fnins.2026.1755119)
Supplement: Supplementary file 1 [file Data_Sheet_1.pdf]

Supplementary Material

1 FEATURE PREDICTIVE POWER ANALYSIS UNDER INPUT NOISE CONDITIONS

In Figures S1-S42, feature importance plots are shown for all analyzed architectures and dataset combinations except the ConvSNN and SpikingResnet18 trained on MNIST dataset, which is included in the main manuscript. Table S1 presents accuracies achieved by XGBoost algorithm on given fold as well as their average.

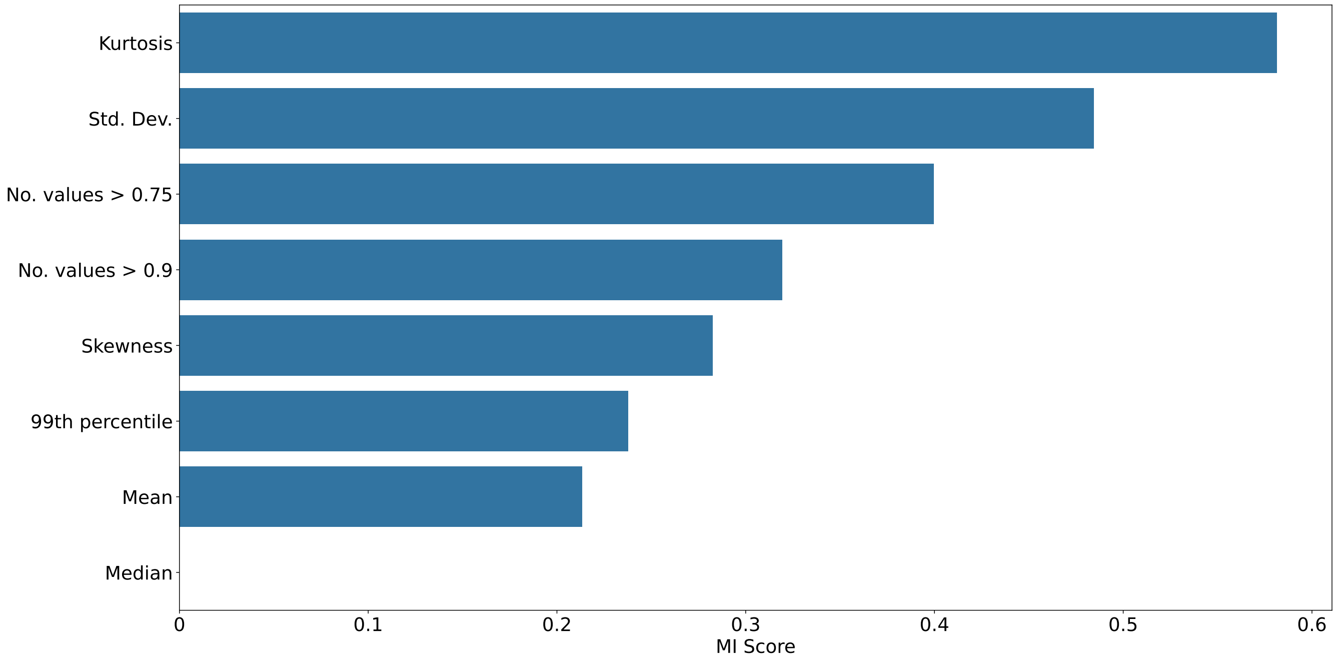

Figure S1: Mutual information scores between given statistic and clean vs. noisy input conditions collected from MLP-SNN trained on MNIST dataset.

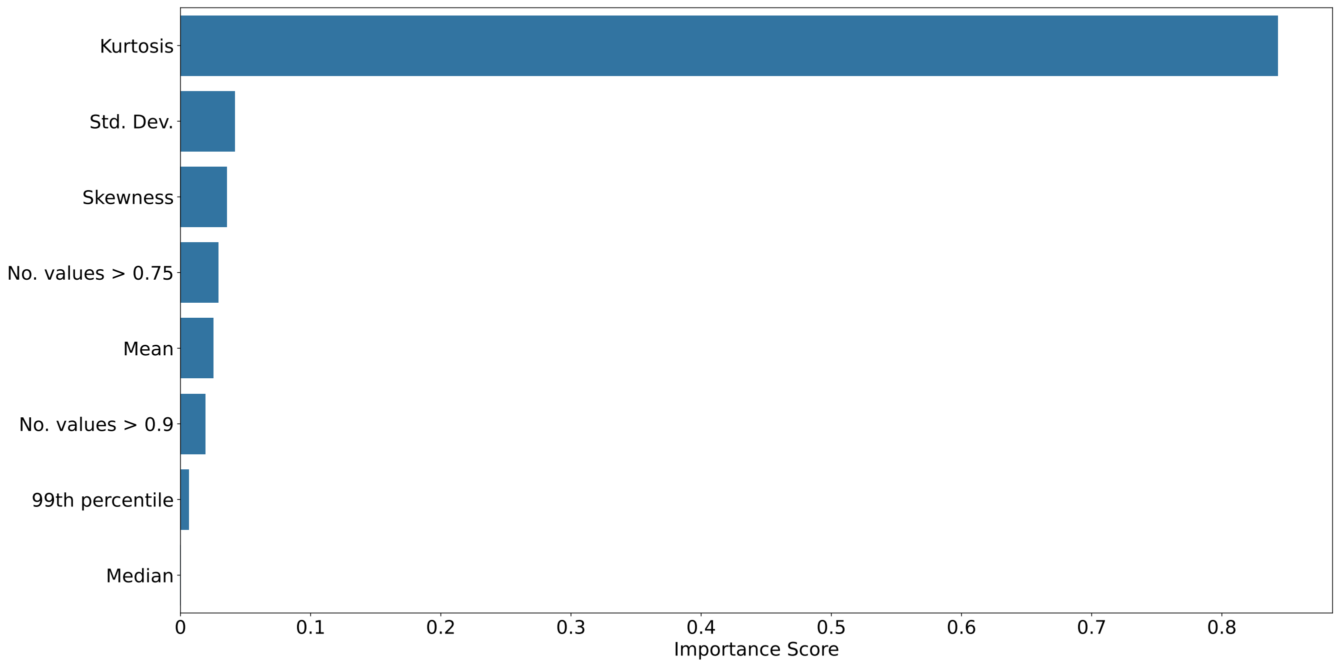

Figure S2: Feature importances from an XGBoost classifier distinguishing clean vs. noisy input conditions collected from MLP-SNN trained on MNIST dataset.

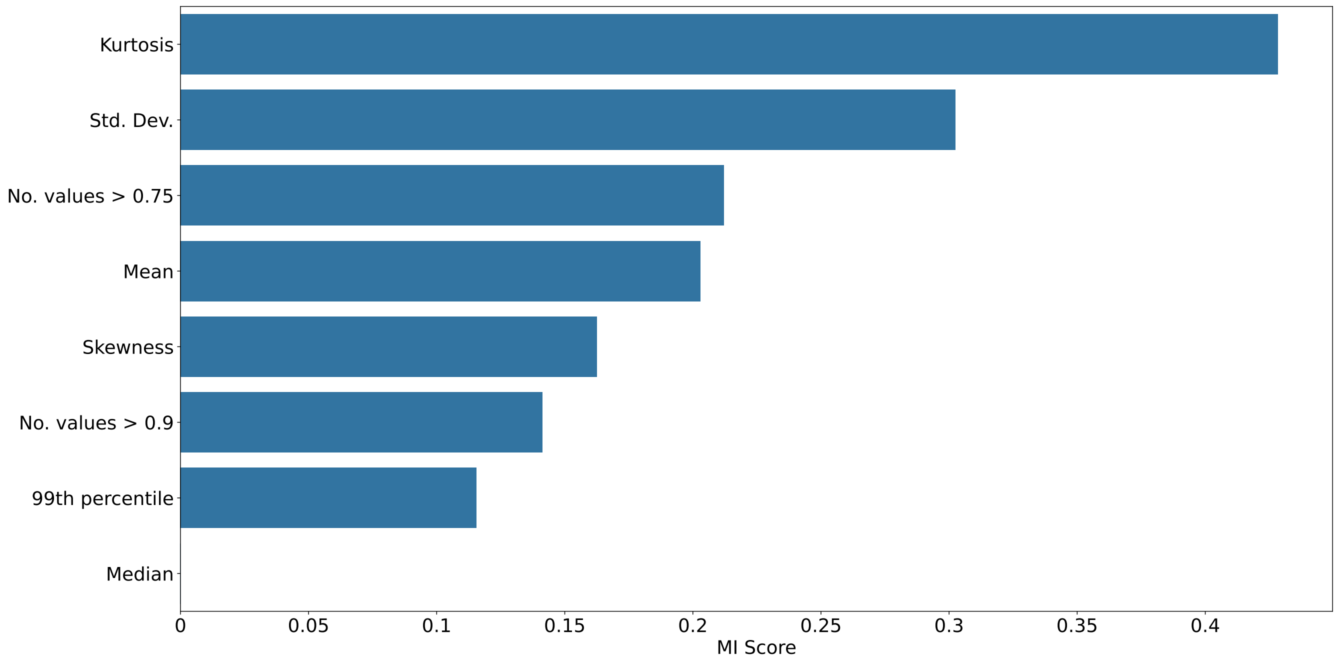

Figure S3: Mutual information scores between given statistic and clean vs. noisy input conditions collected from Recurrent MLP-SNN trained on MNIST dataset.

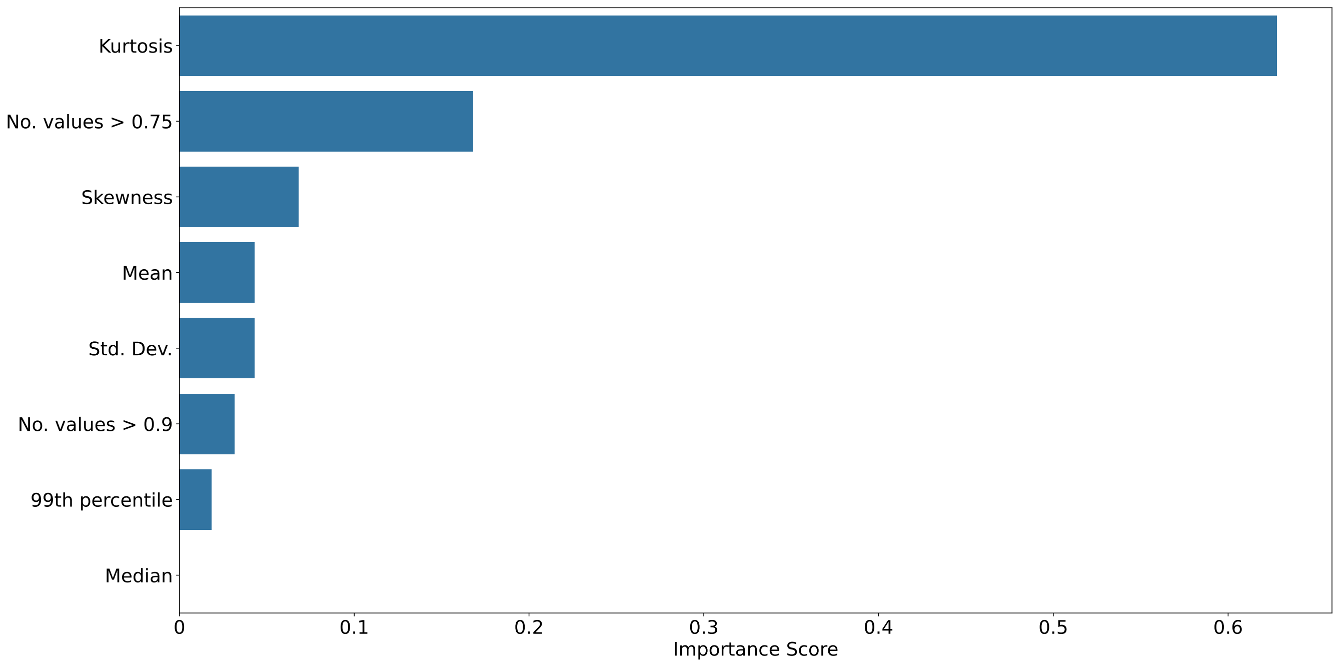

Figure S4: Feature importances from an XGBoost classifier distinguishing clean vs. noisy input conditions collected from Recurrent MLP-SNN trained on MNIST dataset.

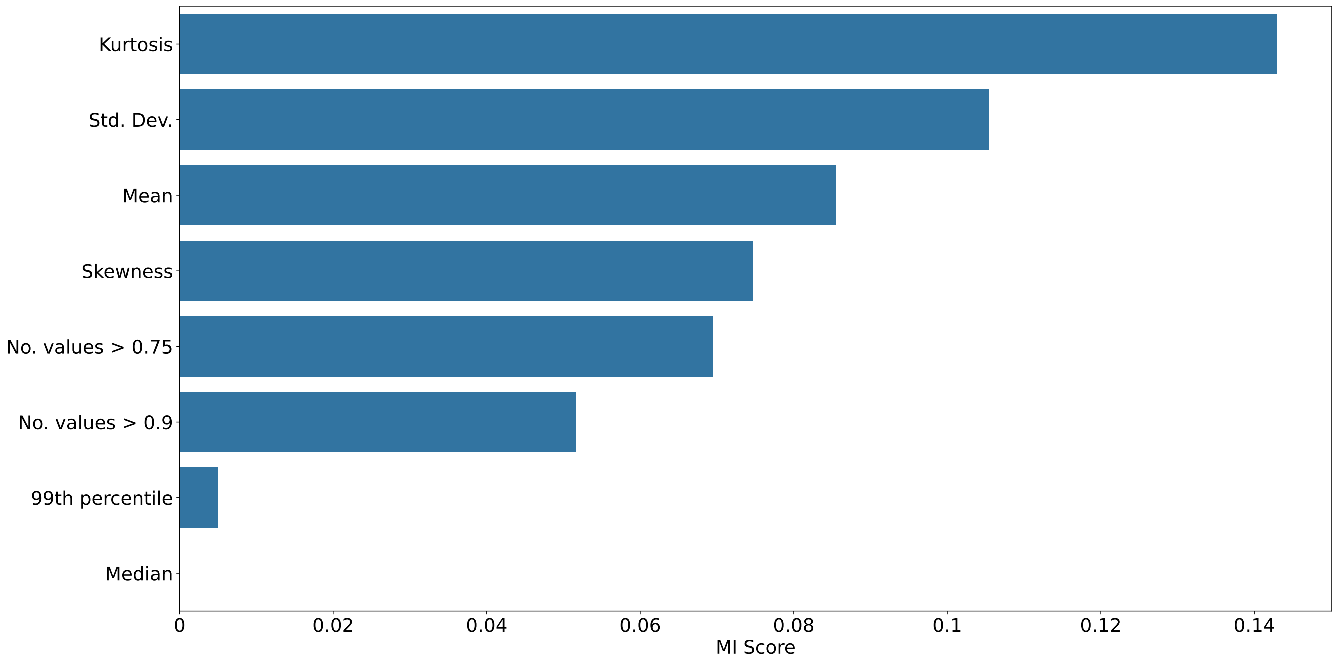

Figure S5: Mutual information scores between given statistic and clean vs. noisy input conditions collected from Recurrent ConvSNN trained on MNIST dataset.

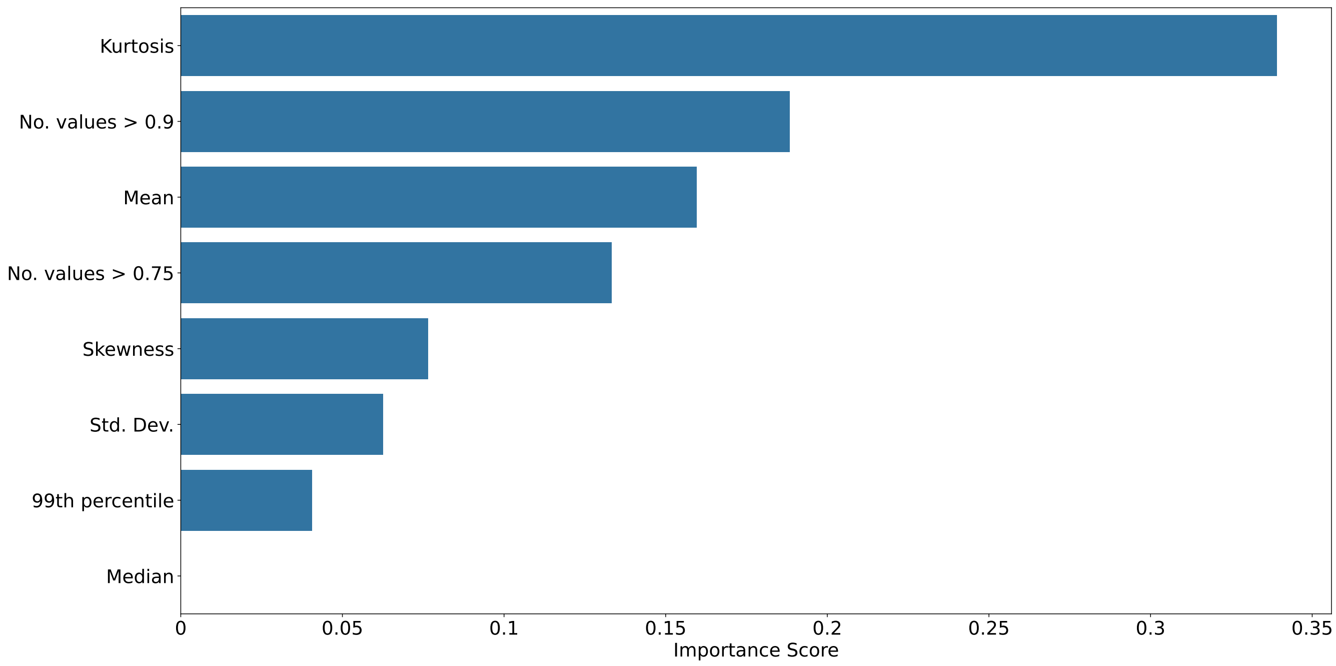

Figure S6: Feature importances from an XGBoost classifier distinguishing clean vs. noisy input conditions collected from Recurrent ConvSNN trained on MNIST dataset.

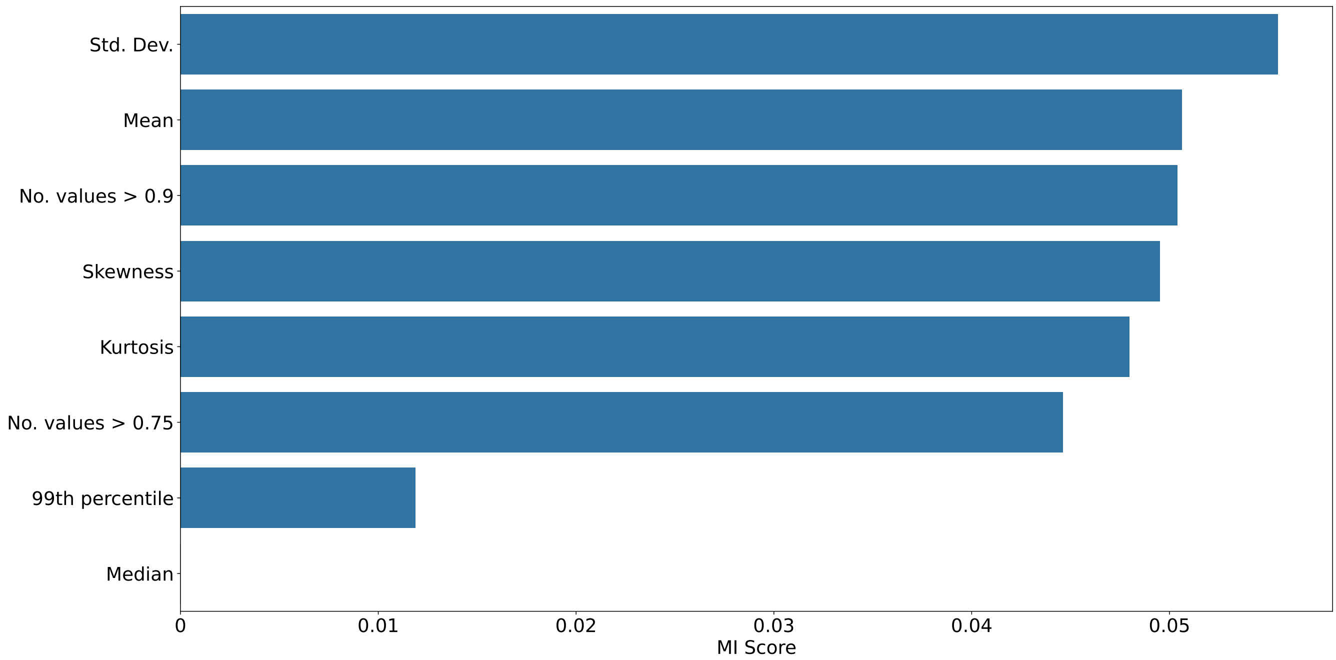

Figure S7: Mutual information scores between given statistic and clean vs. noisy input conditions collected from SpikingVGG11 trained on MNIST dataset.

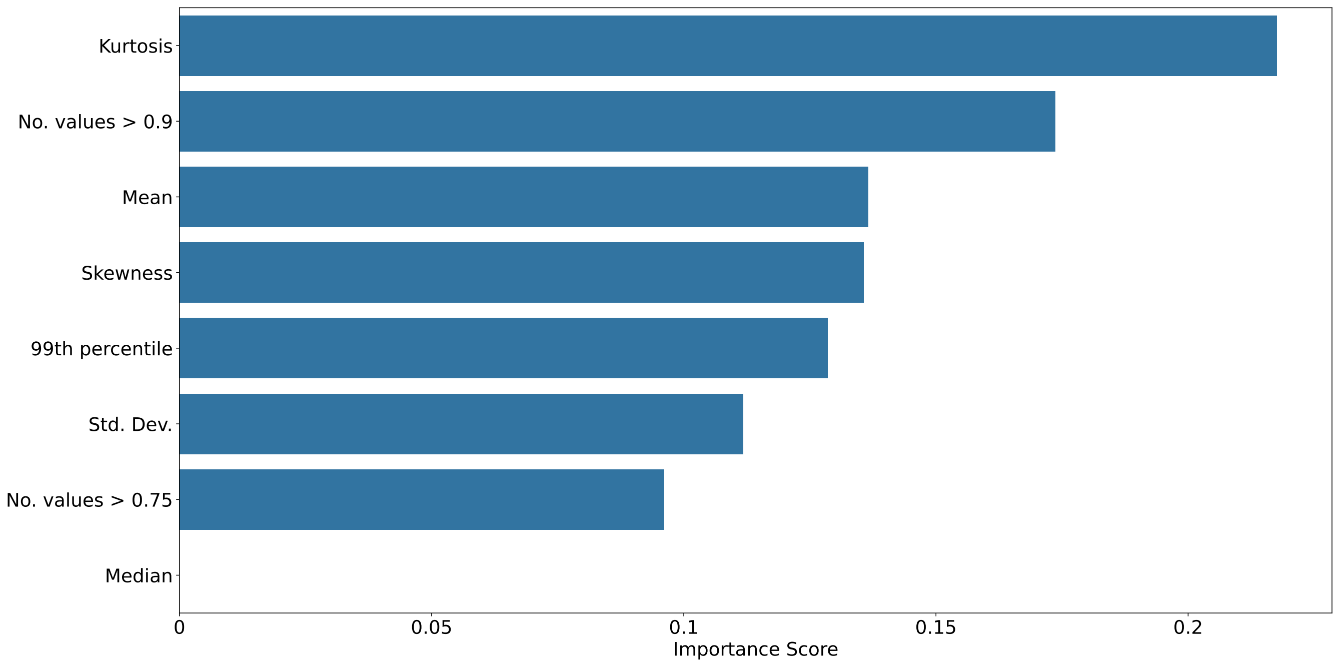

Figure S8: Feature importances from an XGBoost classifier distinguishing clean vs. noisy input conditions collected from SpikingVGG11 trained on MNIST dataset.

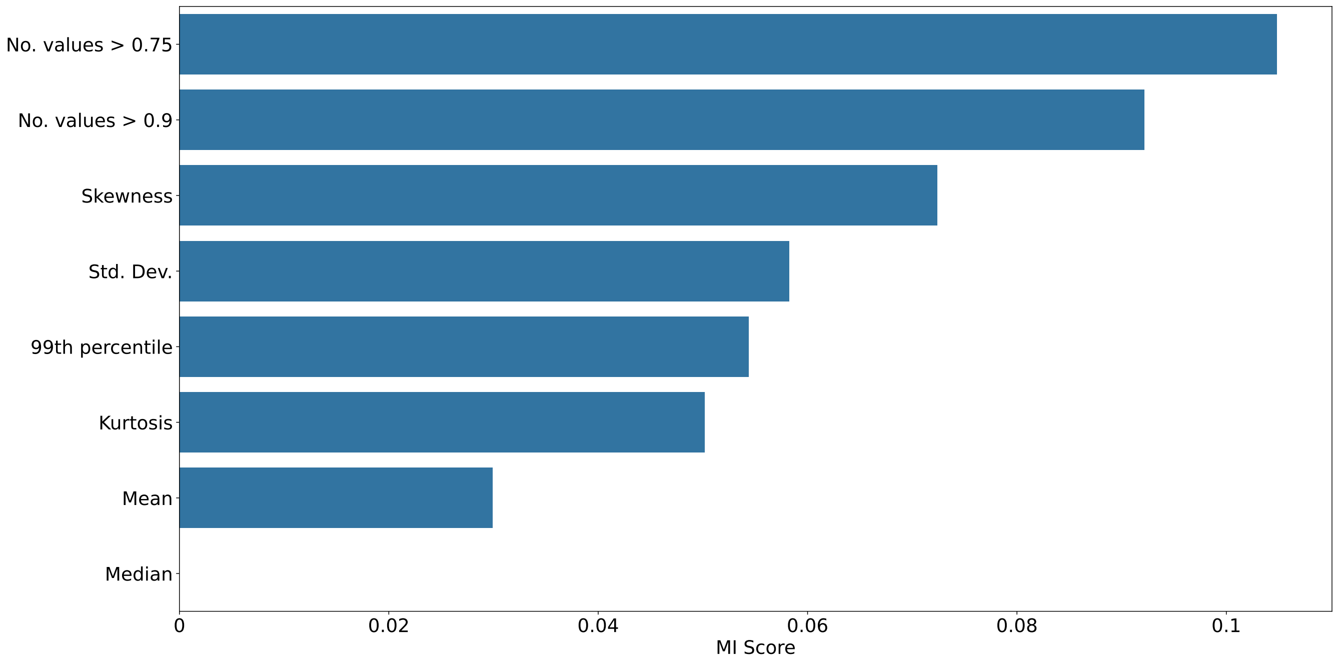

Figure S9: Mutual information scores between given statistic and clean vs. noisy input conditions collected from MLP-SNN trained on CIFAR-10 dataset.

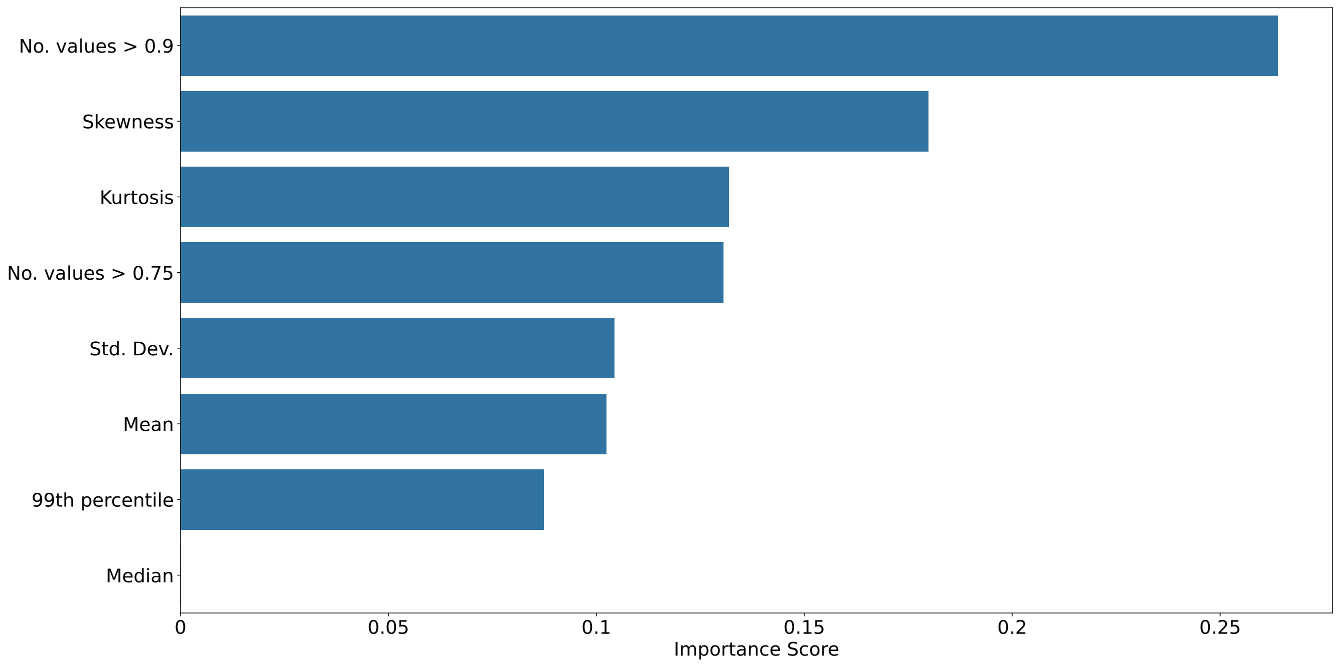

Figure S10: Feature importances from an XGBoost classifier distinguishing clean vs. noisy input conditions collected from MLP-SNN trained on CIFAR-10 dataset.

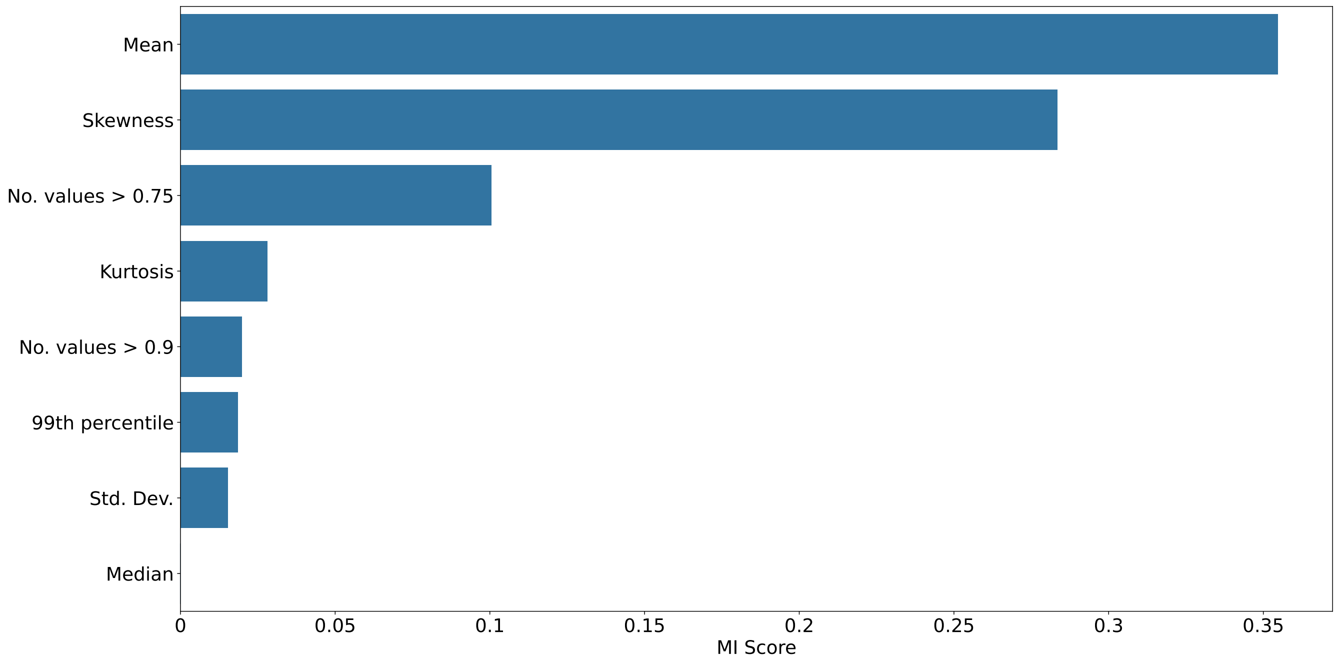

Figure S11: Mutual information scores between given statistic and clean vs. noisy input conditions collected from ConvSNN trained on CIFAR-10 dataset.

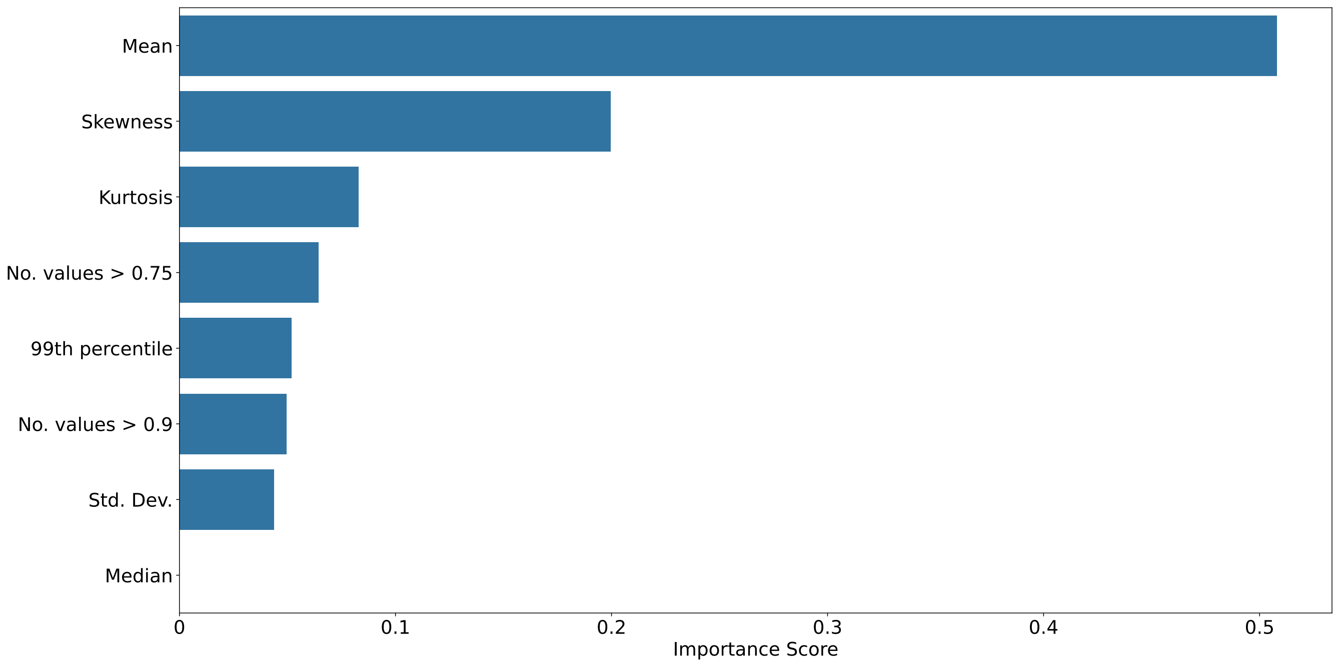

Figure S12: Feature importances from an XGBoost classifier distinguishing clean vs. noisy input conditions collected from ConvSNN trained on CIFAR-10 dataset.

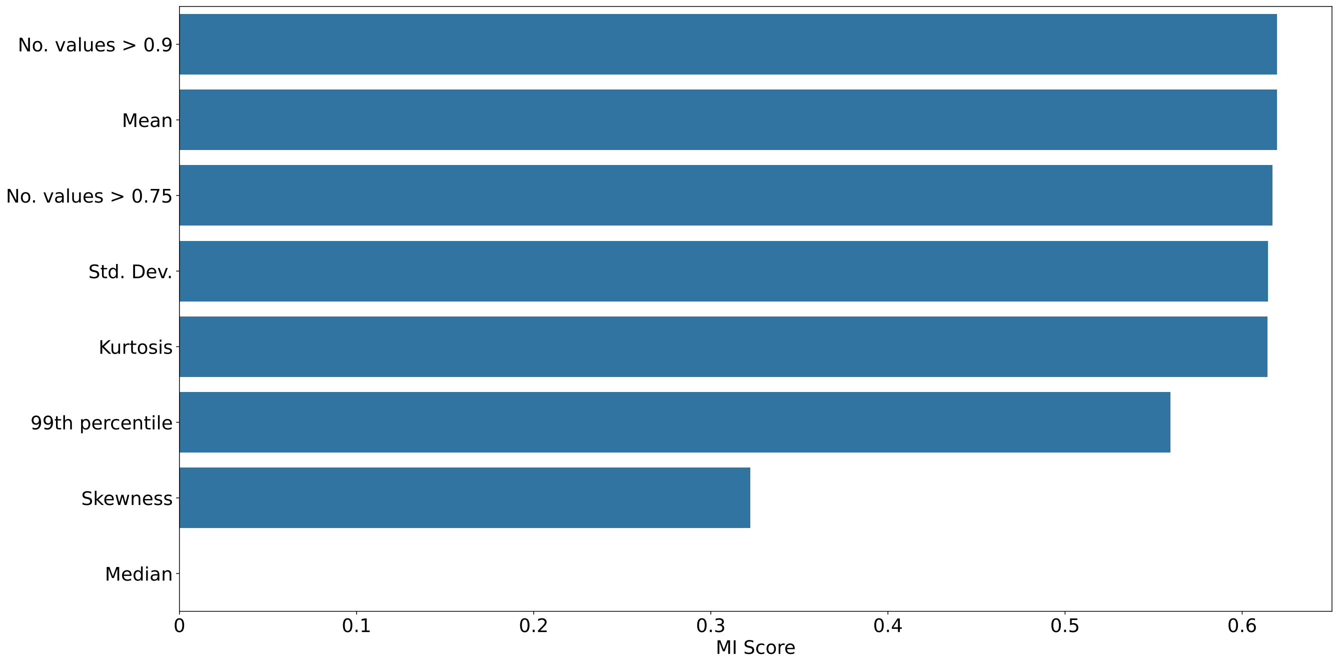

Figure S13: Mutual information scores between given statistic and clean vs. noisy input conditions collected from Recurrent MLP-SNN trained on CIFAR-10 dataset.

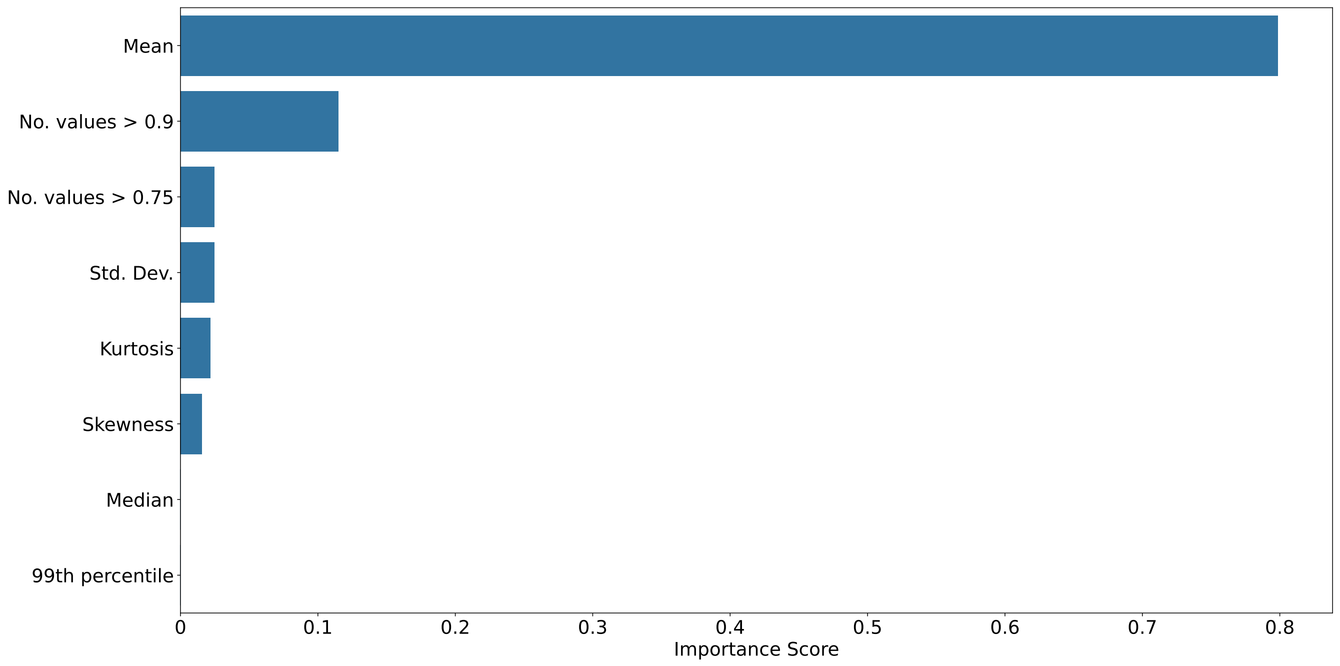

Figure S14: Feature importances from an XGBoost classifier distinguishing clean vs. noisy input conditions collected from Recurrent MLP-SNN trained on CIFAR-10 dataset.

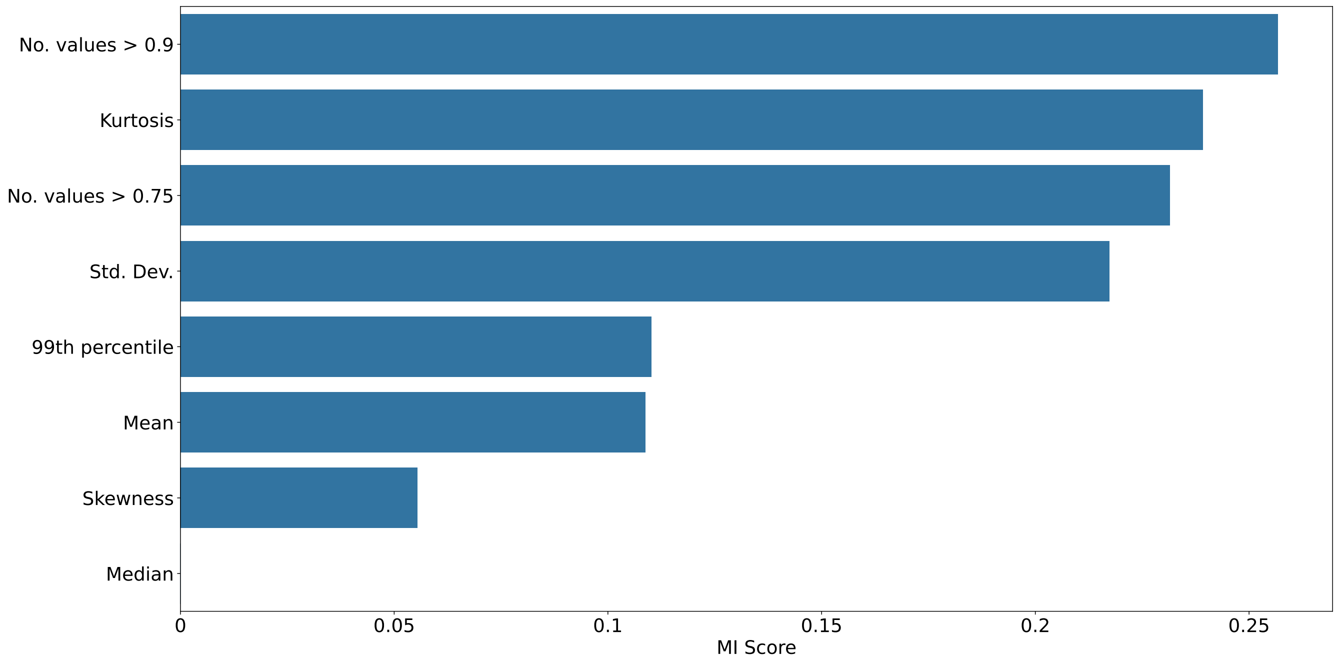

Figure S15: Mutual information scores between given statistic and clean vs. noisy input conditions collected from Recurrent ConvSNN trained on CIFAR-10 dataset.

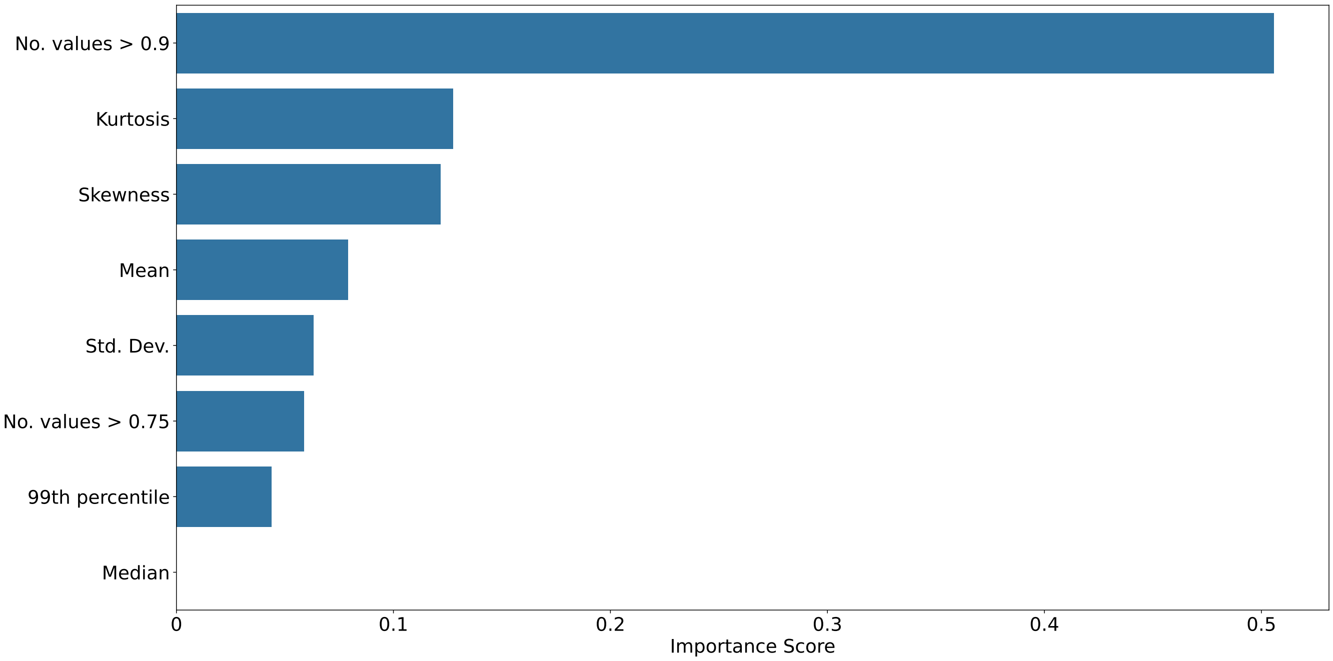

Figure S16: Feature importances from an XGBoost classifier distinguishing clean vs. noisy input conditions collected from Recurrent ConvSNN trained on CIFAR-10 dataset.

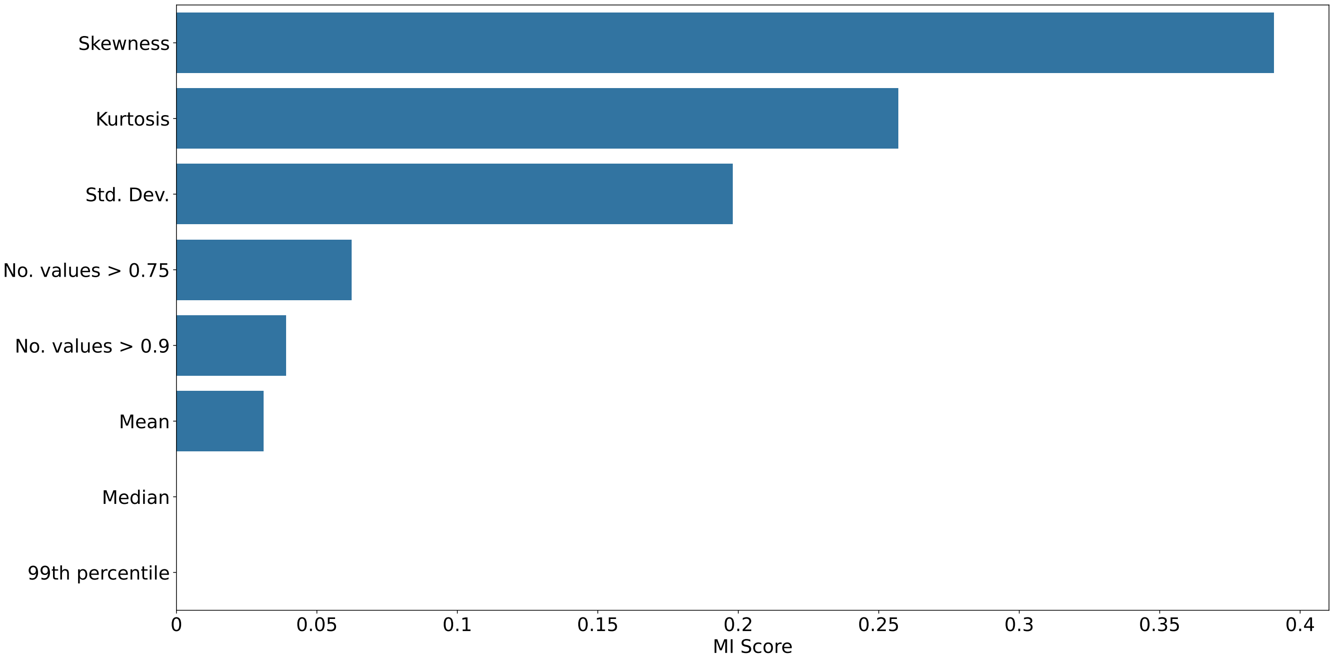

Figure S17: Mutual information scores between given statistic and clean vs. noisy input conditions collected from SpikingResnet18 trained on CIFAR-10 dataset.

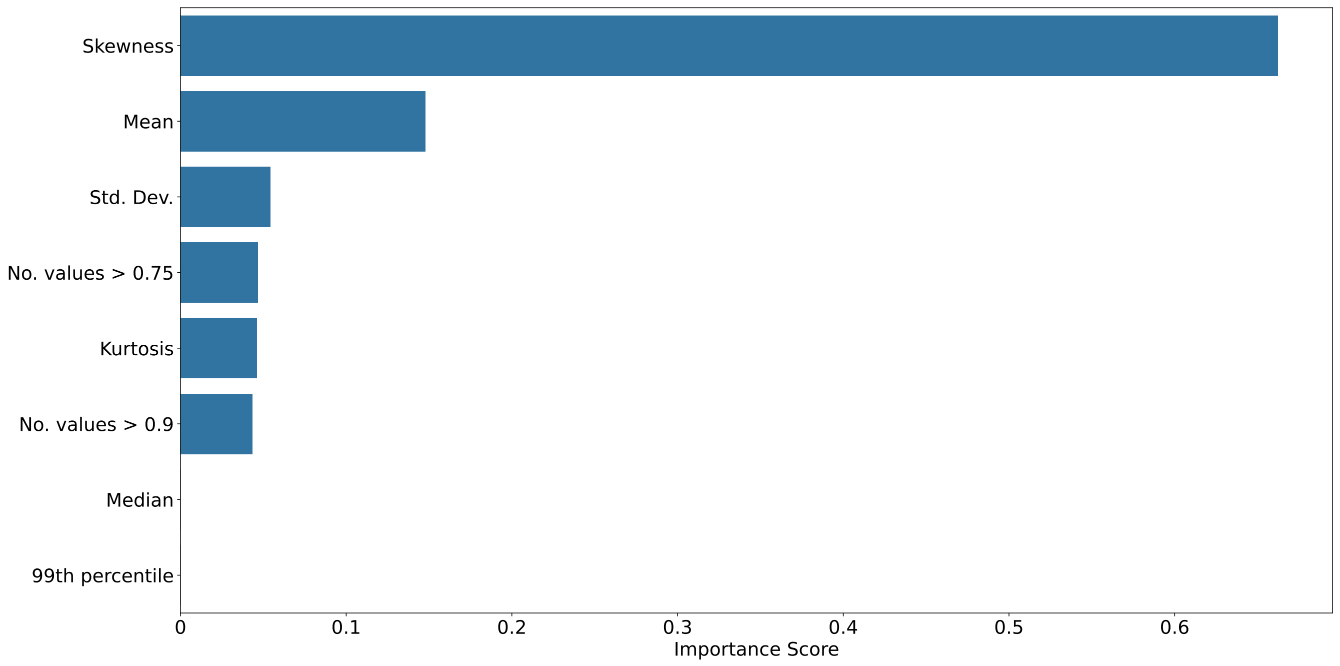

Figure S18: Feature importances from an XGBoost classifier distinguishing clean vs. noisy input conditions collected from SpikingResnet18 trained on CIFAR-10 dataset.

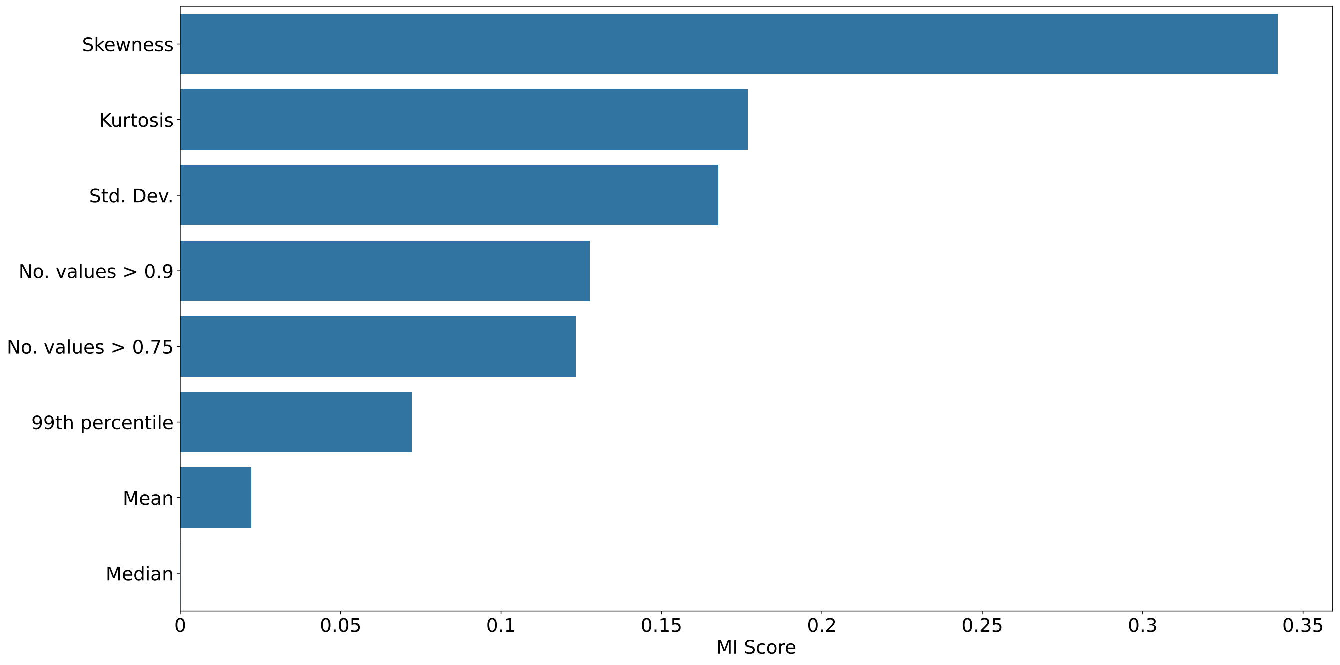

Figure S19: Mutual information scores between given statistic and clean vs. noisy input conditions collected from SpikingVGG11 trained on CIFAR-10 dataset.

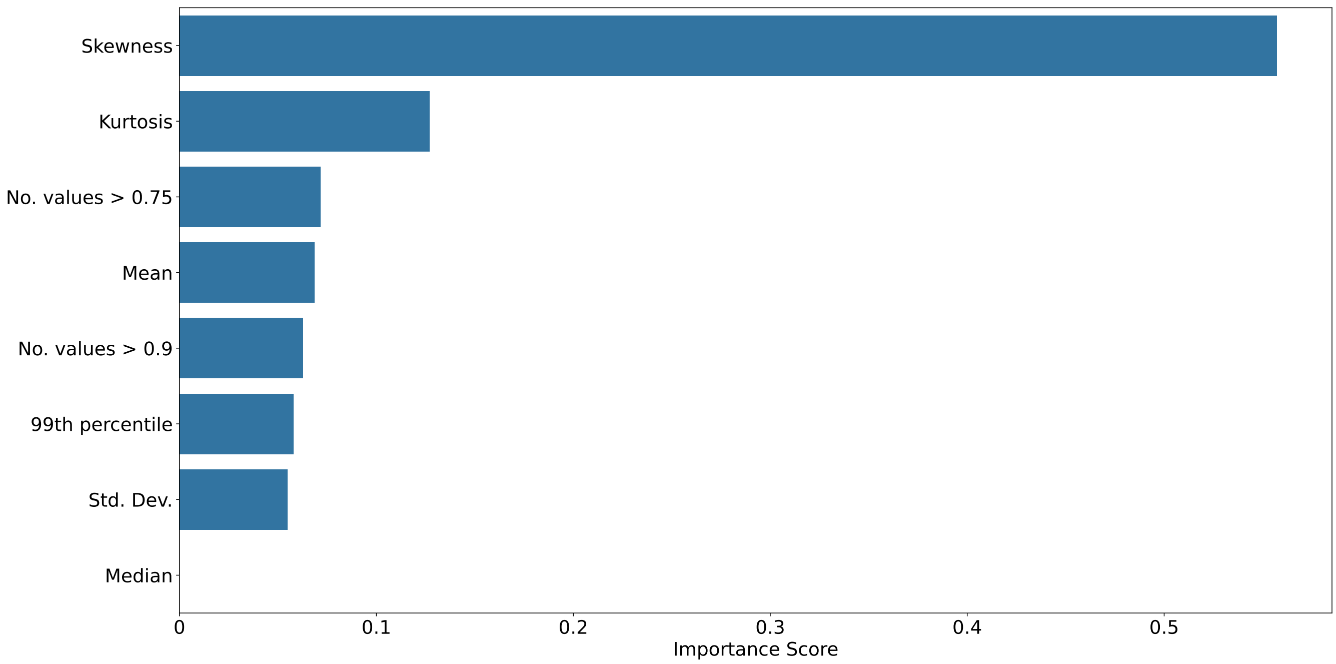

Figure S20: Feature importances from an XGBoost classifier distinguishing clean vs. noisy input conditions collected from SpikingVGG11 trained on CIFAR-10 dataset.

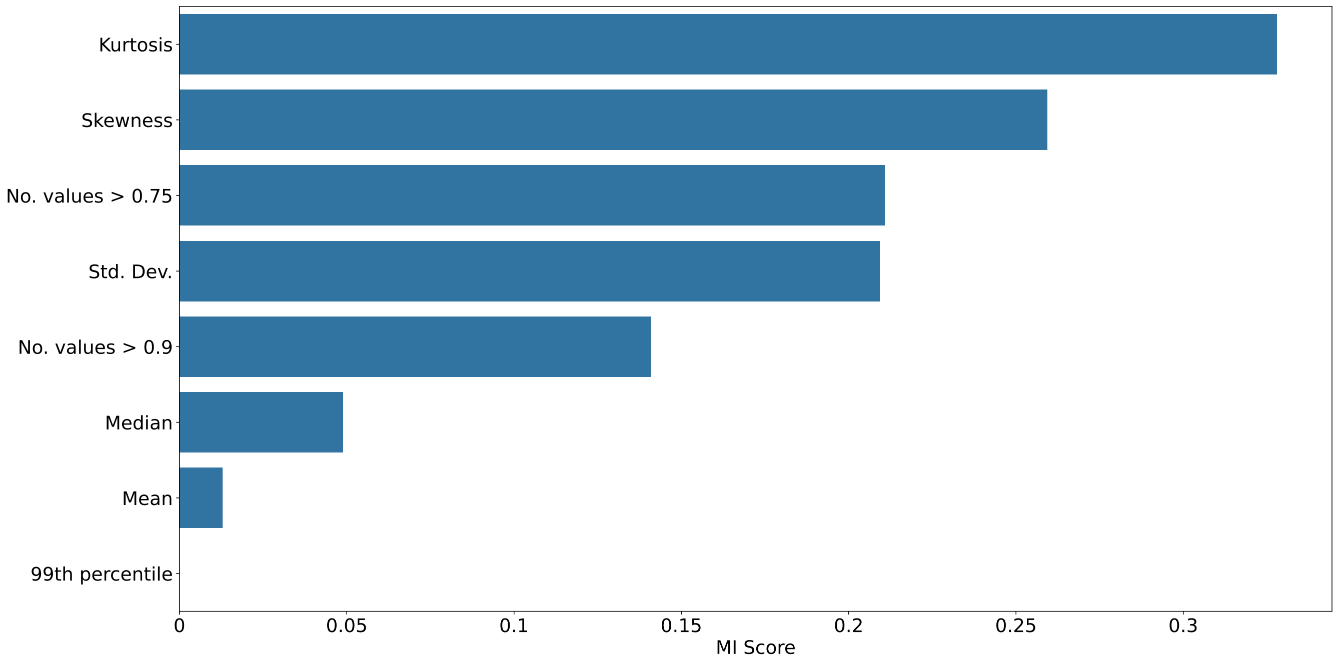

Figure S21: Mutual information scores between given statistic and clean vs. noisy input conditions collected from MLP-SNN trained on EventMNIST dataset.

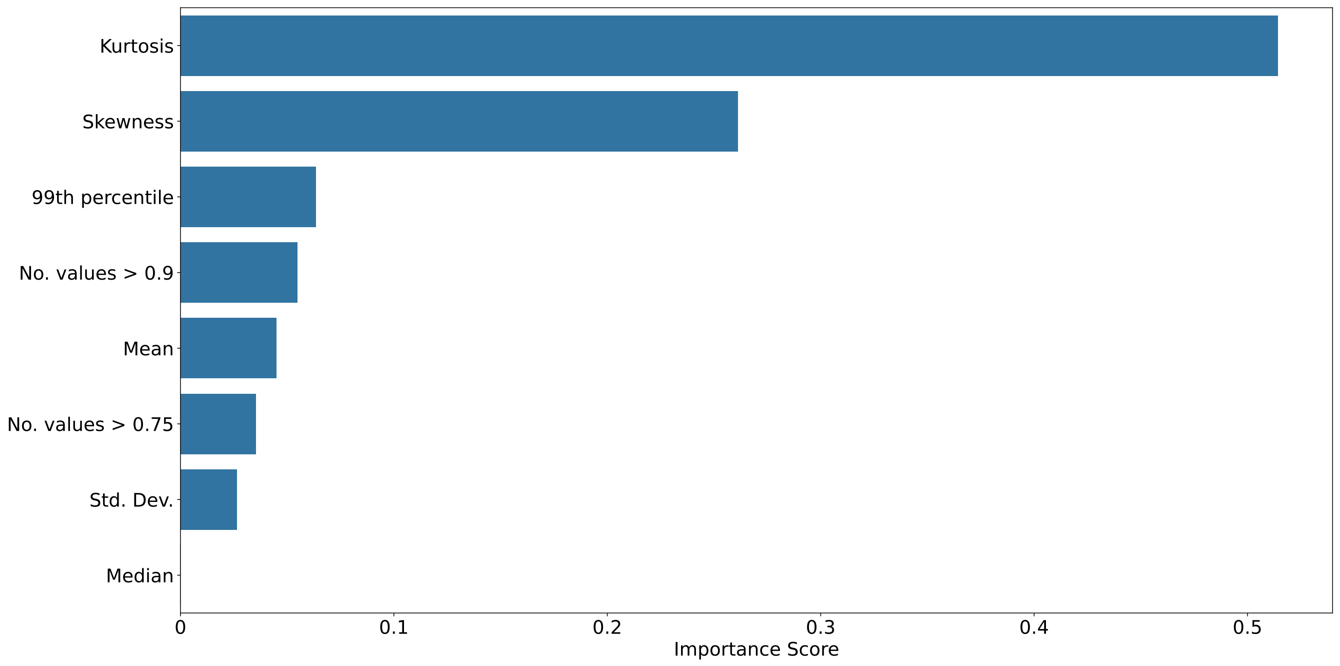

Figure S22: Feature importances from an XGBoost classifier distinguishing clean vs. noisy input conditions collected from MLP-SNN trained on EventMNIST dataset.

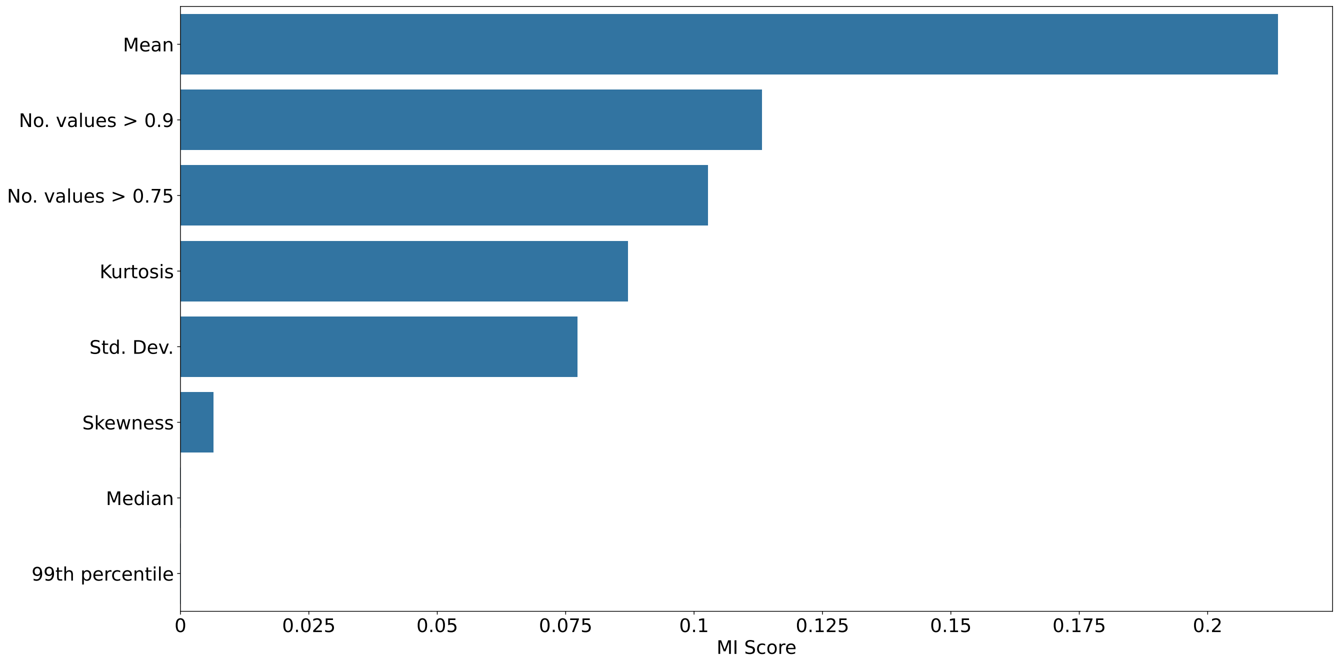

Figure S23: Mutual information scores between given statistic and clean vs. noisy input conditions collected from Recurrent MLP-SNN trained on EventMNIST dataset.

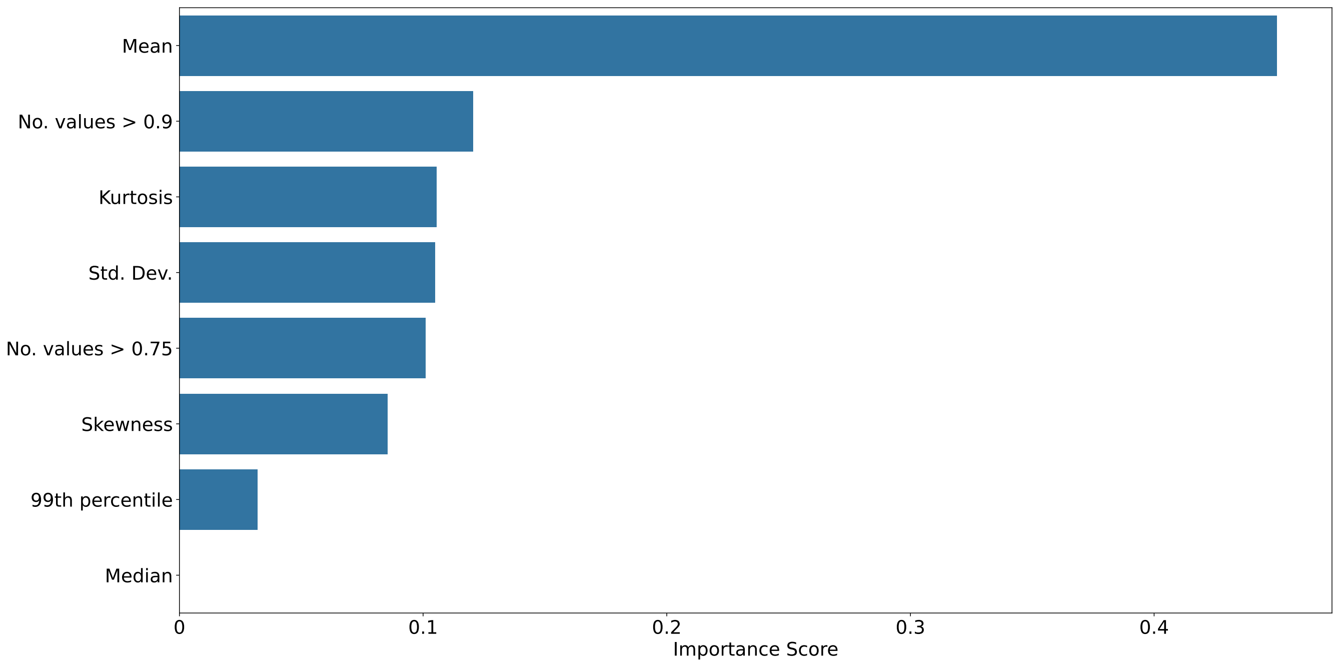

Figure S24: Feature importances from an XGBoost classifier distinguishing clean vs. noisy input conditions collected from Recurrent MLP-SNN trained on EventMNIST dataset.

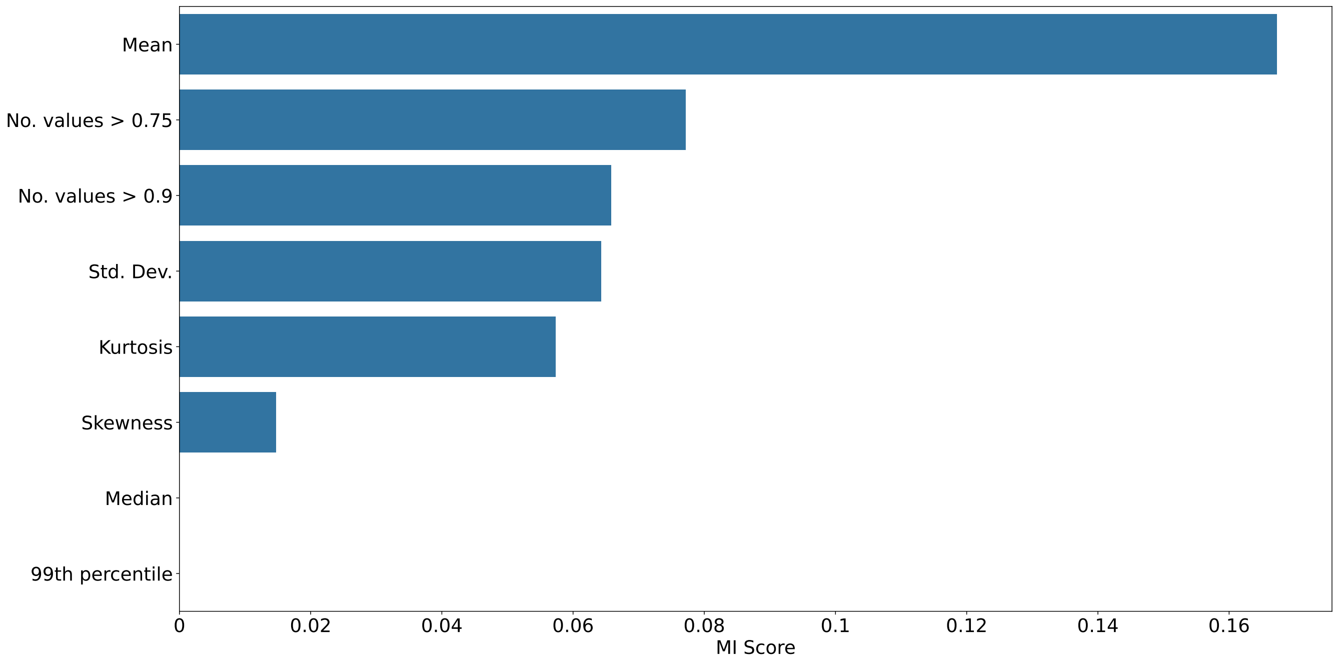

Figure S25: Mutual information scores between given statistic and clean vs. noisy input conditions collected from Recurrent ConvSNN trained on EventMNIST dataset.

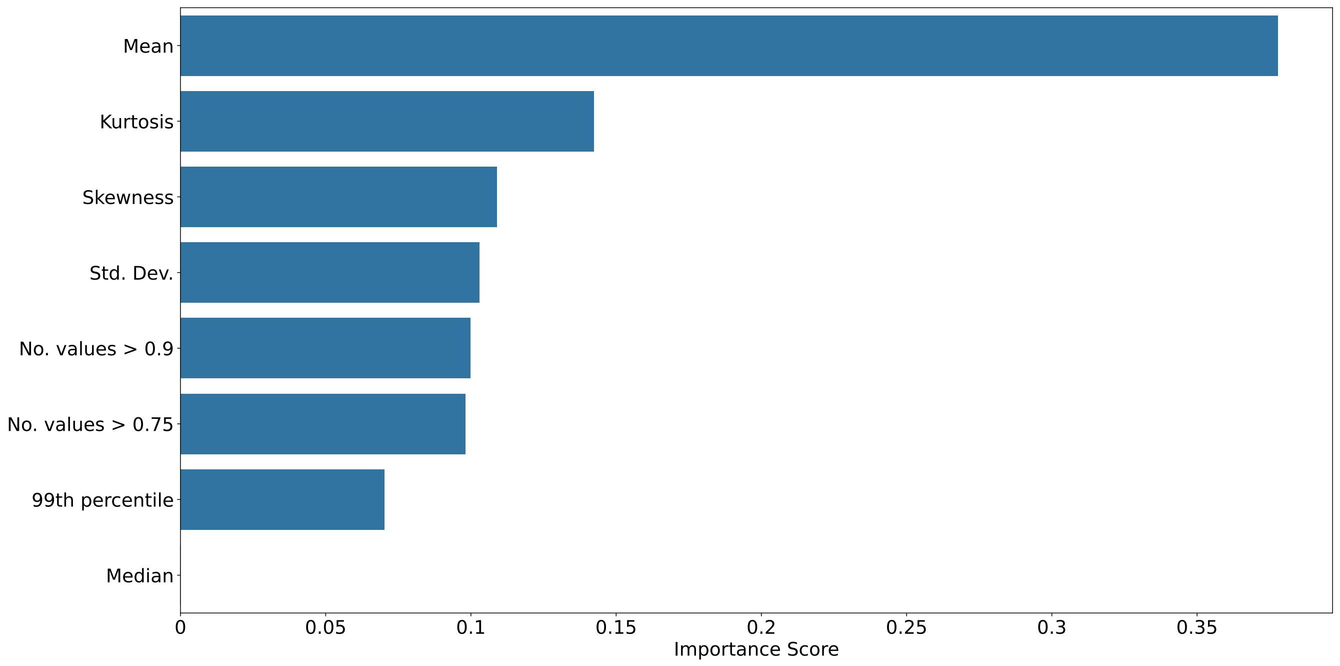

Figure S26: Feature importances from an XGBoost classifier distinguishing clean vs. noisy input conditions collected from Recurrent ConvSNN trained on EventMNIST dataset.

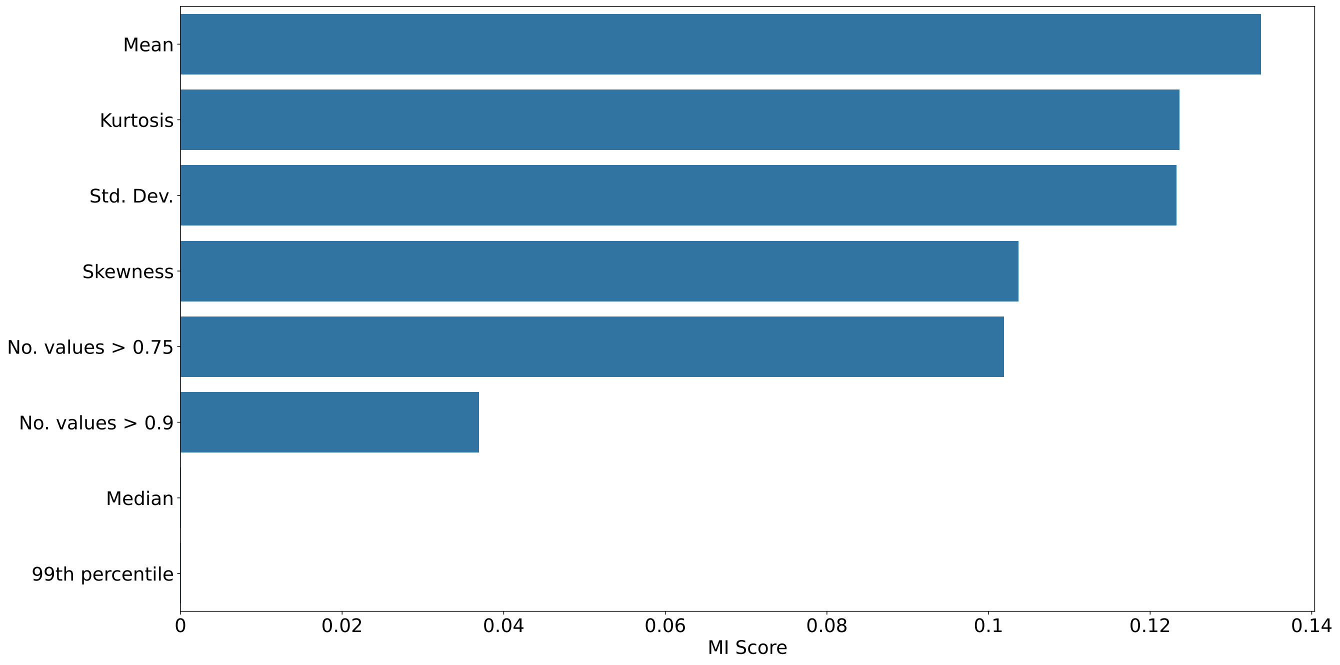

Figure S27: Mutual information scores between given statistic and clean vs. noisy input conditions collected from SpikingResnet18 trained on EventMNIST dataset.

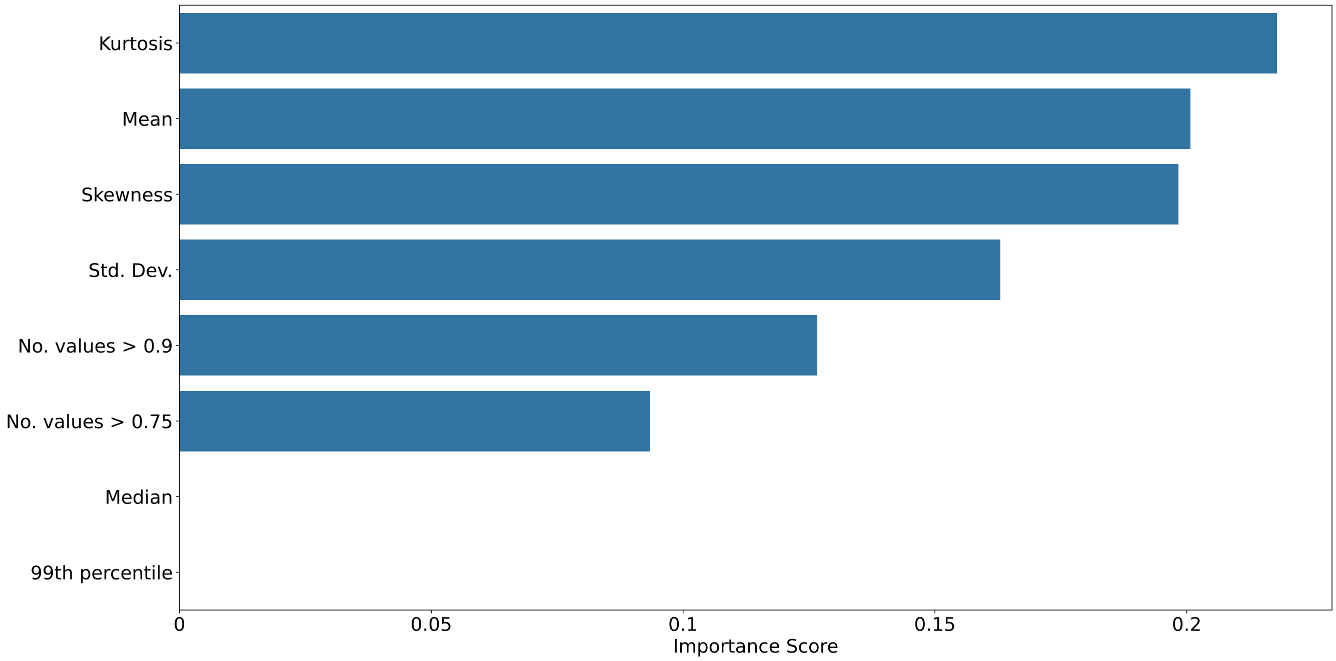

Figure S28: Feature importances from an XGBoost classifier distinguishing clean vs. noisy input conditions collected from SpikingResnet18 trained on EventMNIST dataset.

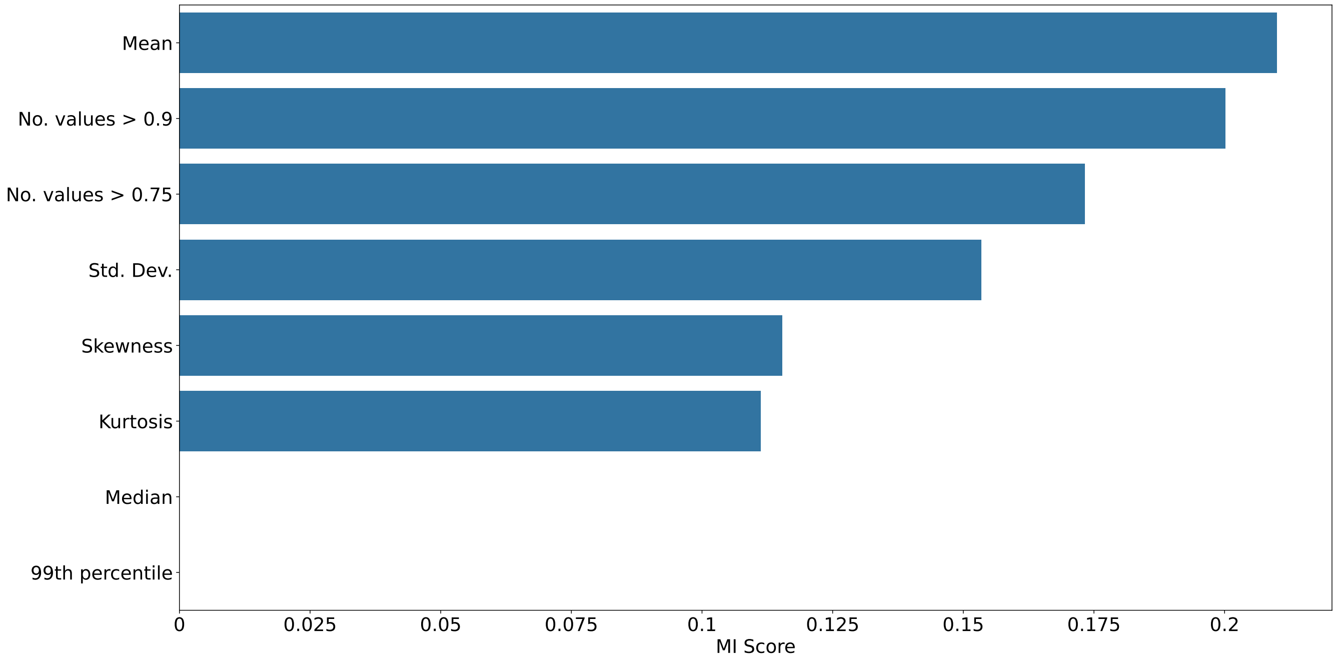

Figure S29: Mutual information scores between given statistic and clean vs. noisy input conditions collected from SpikingVGG11 trained on EventMNIST dataset.

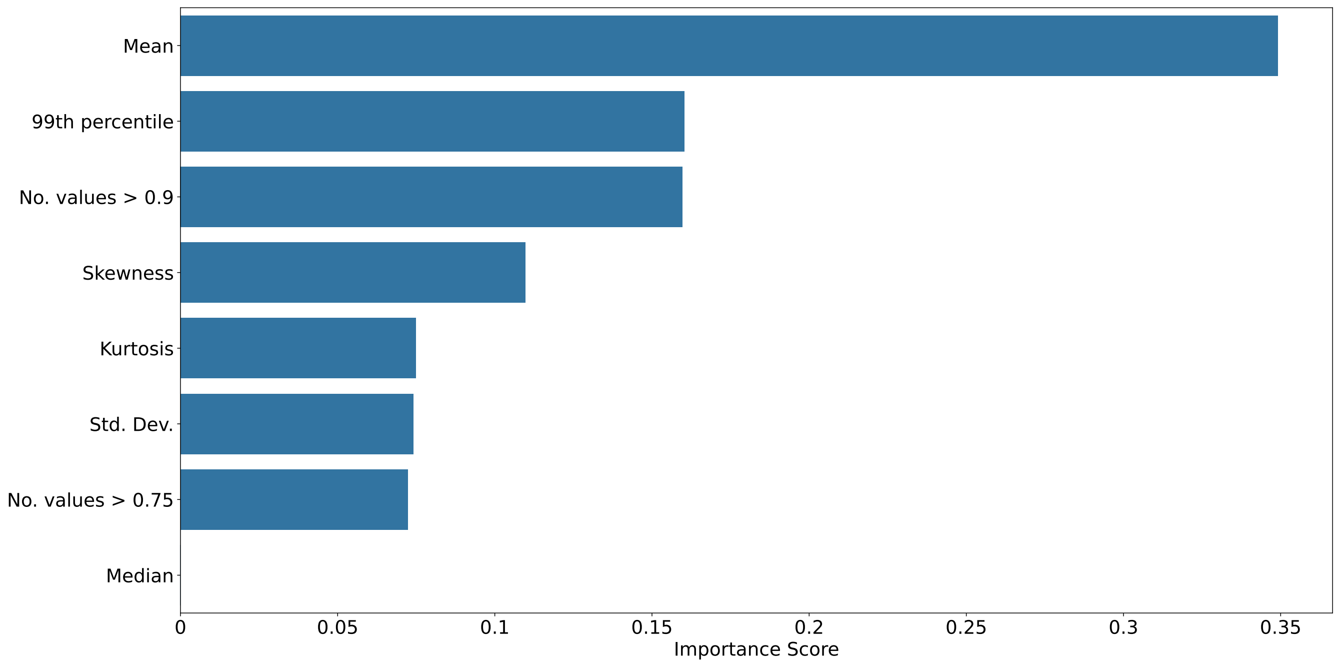

Figure S30: Feature importances from an XGBoost classifier distinguishing clean vs. noisy input conditions collected from SpikingVGG11 trained on EventMNIST dataset.

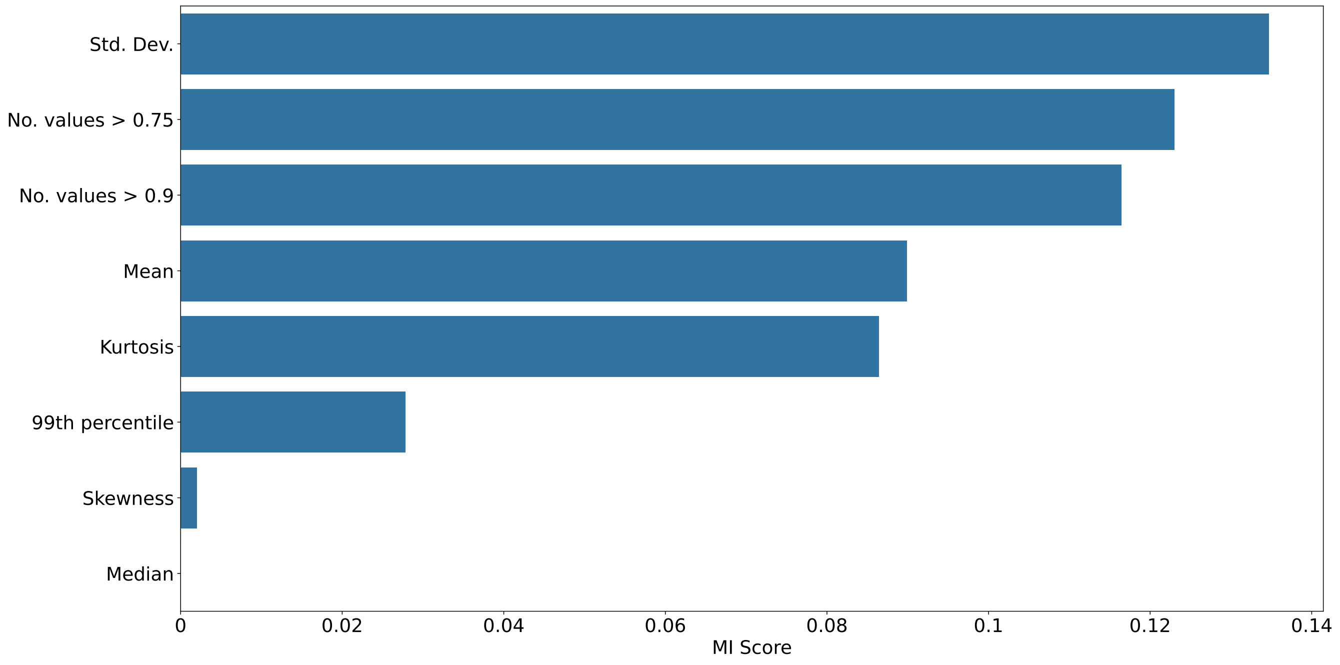

Figure S31: Mutual information scores between given statistic and clean vs. noisy input conditions collected from MLP-SNN trained on UCF11 dataset.

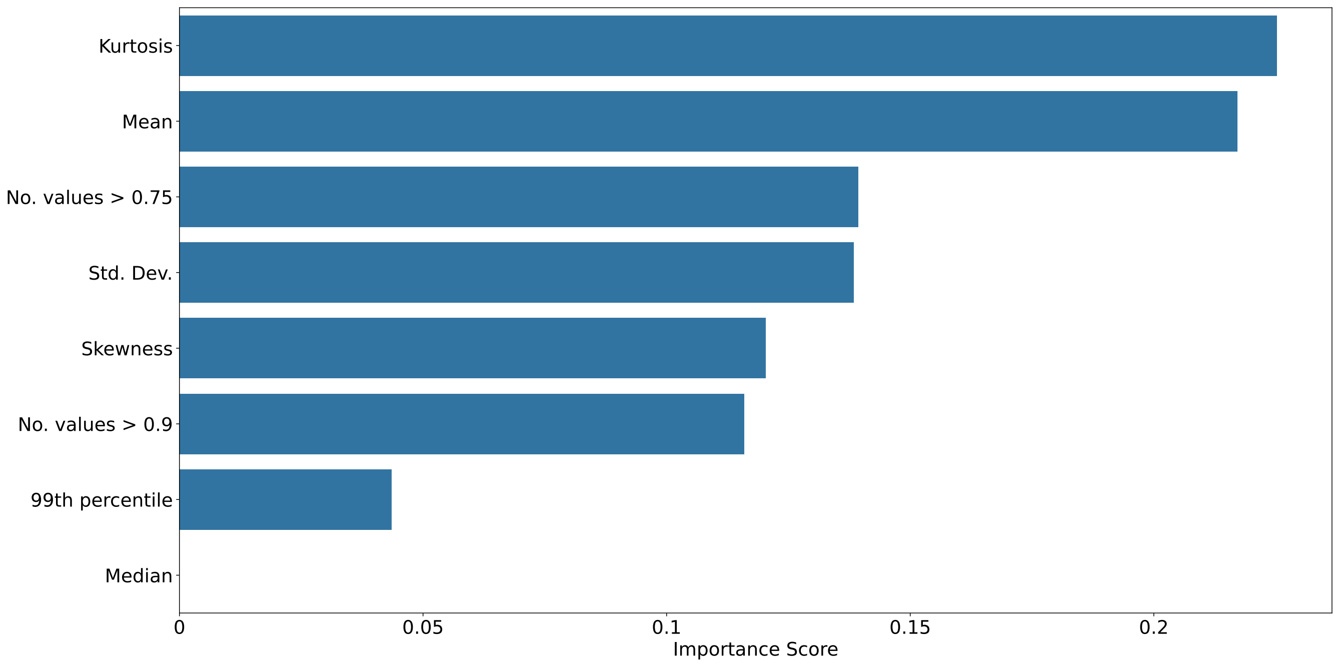

Figure S32: Feature importances from an XGBoost classifier distinguishing clean vs. noisy input conditions collected from MLP-SNN trained on UCF11 dataset.

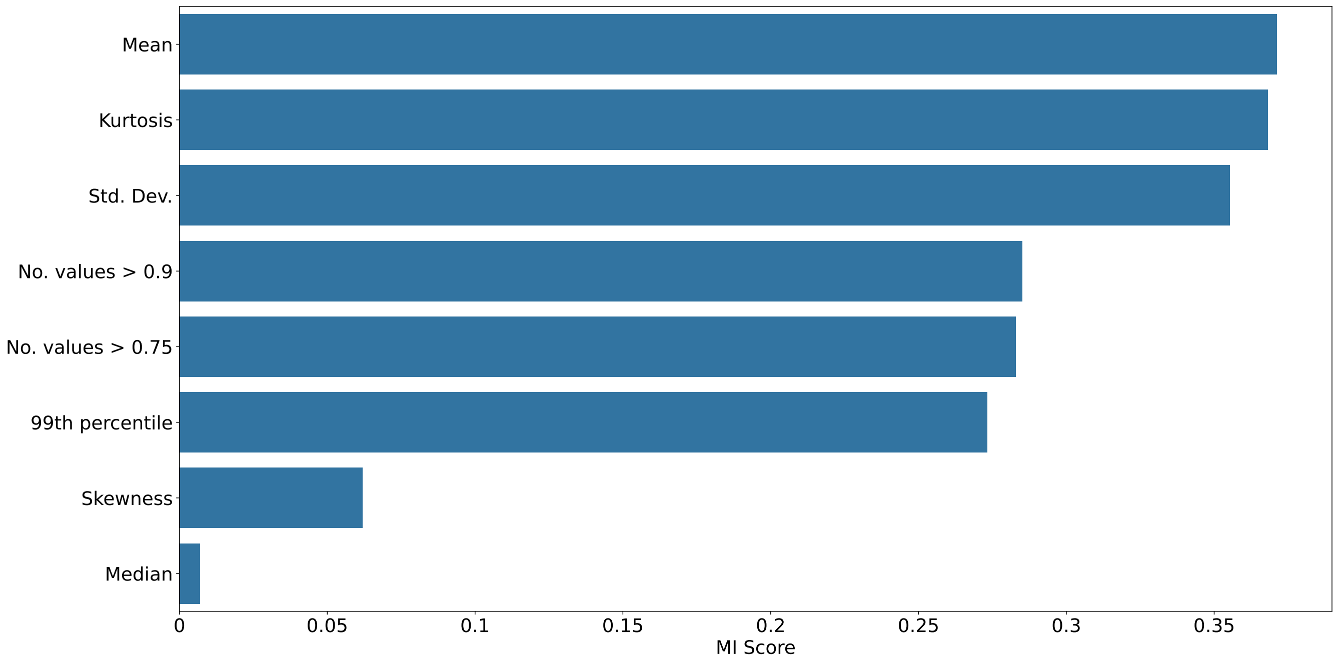

Figure S33: Mutual information scores between given statistic and clean vs. noisy input conditions collected from ConvSNN trained on UCF11 dataset.

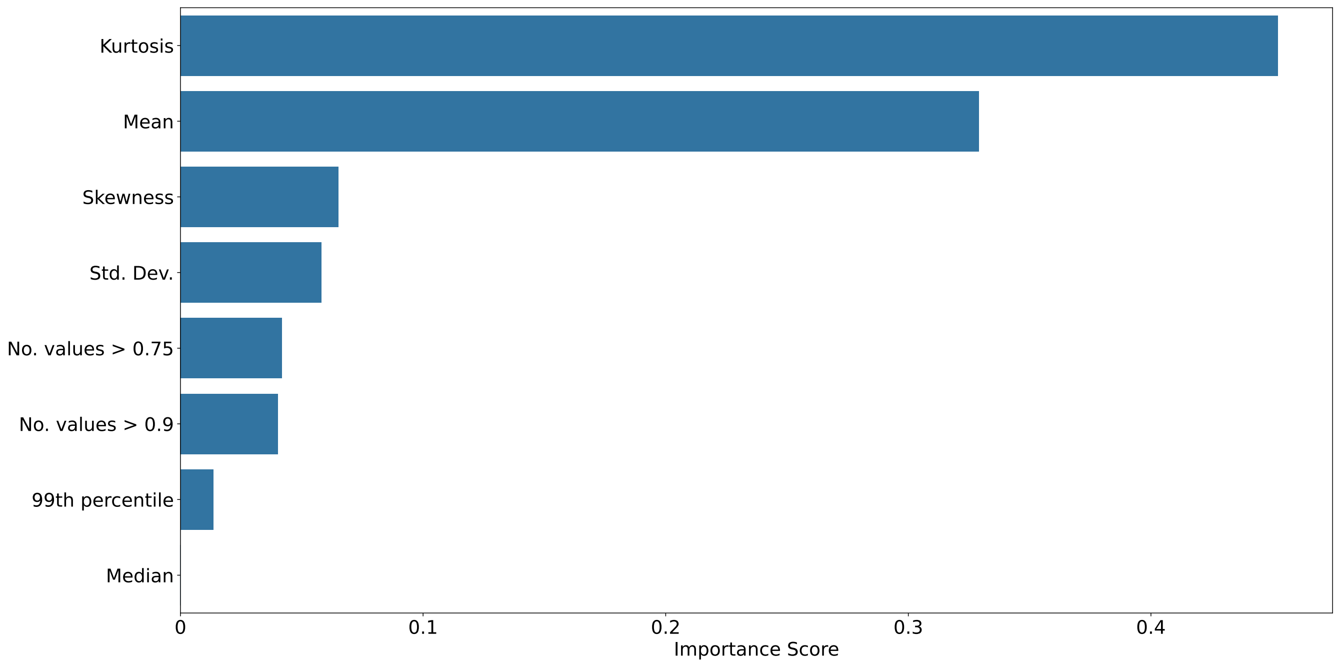

Figure S34: Feature importances from an XGBoost classifier distinguishing clean vs. noisy input conditions collected from ConvSNN trained on UCF11 dataset.

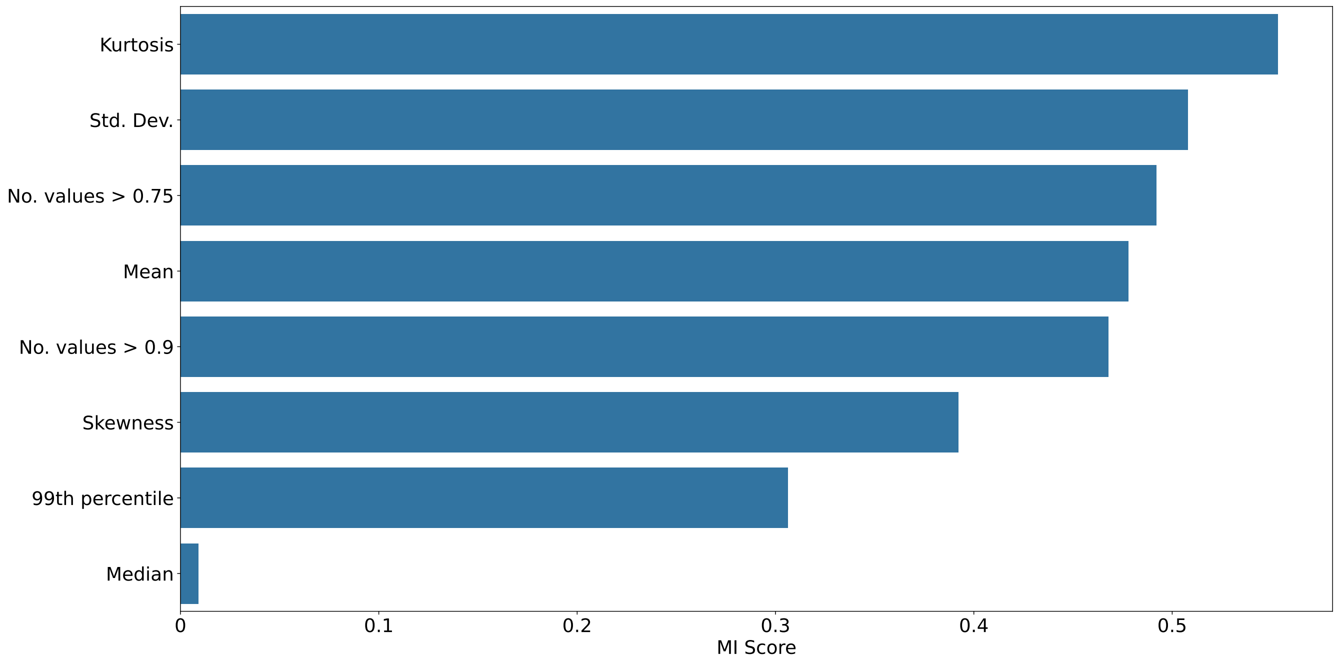

Figure S35: Mutual information scores between given statistic and clean vs. noisy input conditions collected from Recurrent MLP-SNN trained on UCF11 dataset.

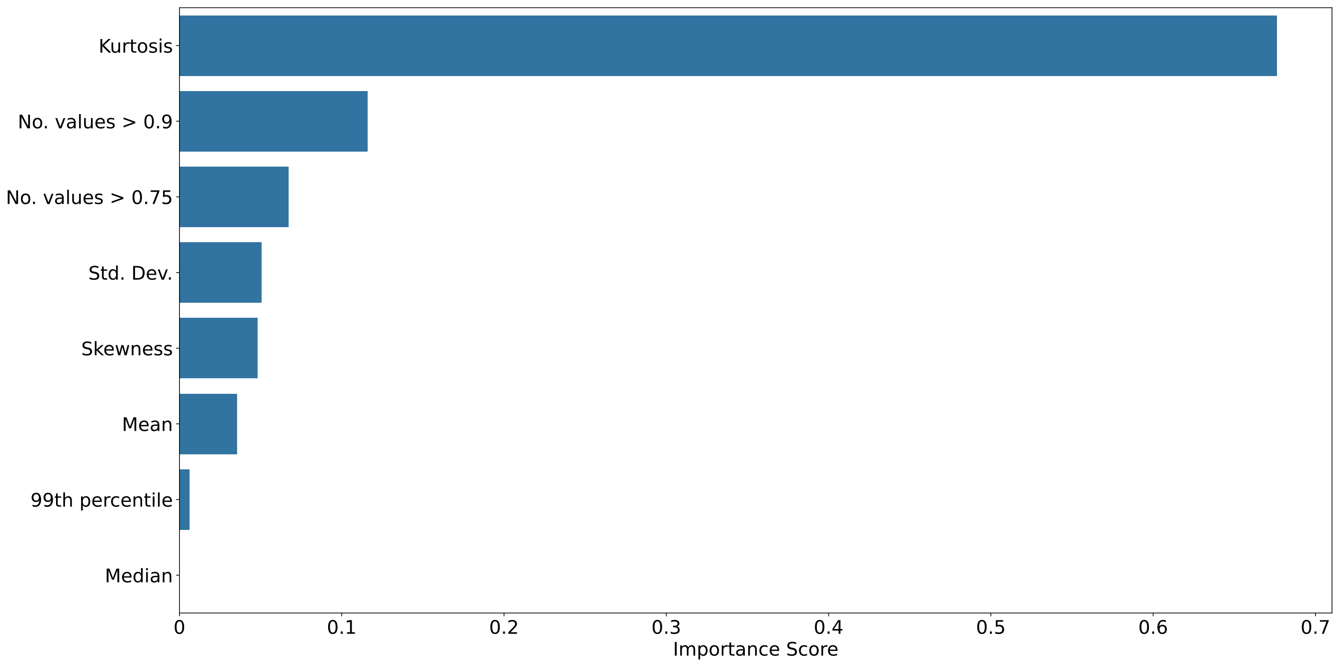

Figure S36: Feature importances from an XGBoost classifier distinguishing clean vs. noisy input conditions collected from Recurrent MLP-SNN trained on UCF11 dataset.

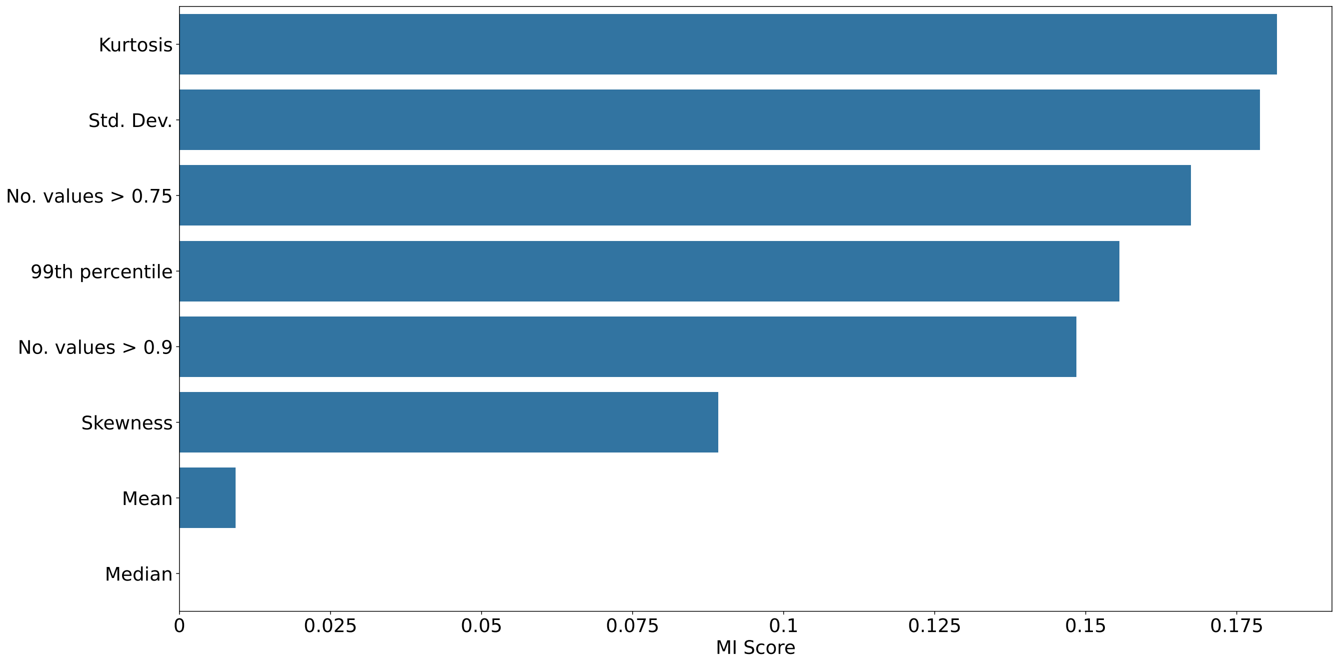

Figure S37: Mutual information scores between given statistic and clean vs. noisy input conditions collected from Recurrent ConvSNN trained on UCF11 dataset.

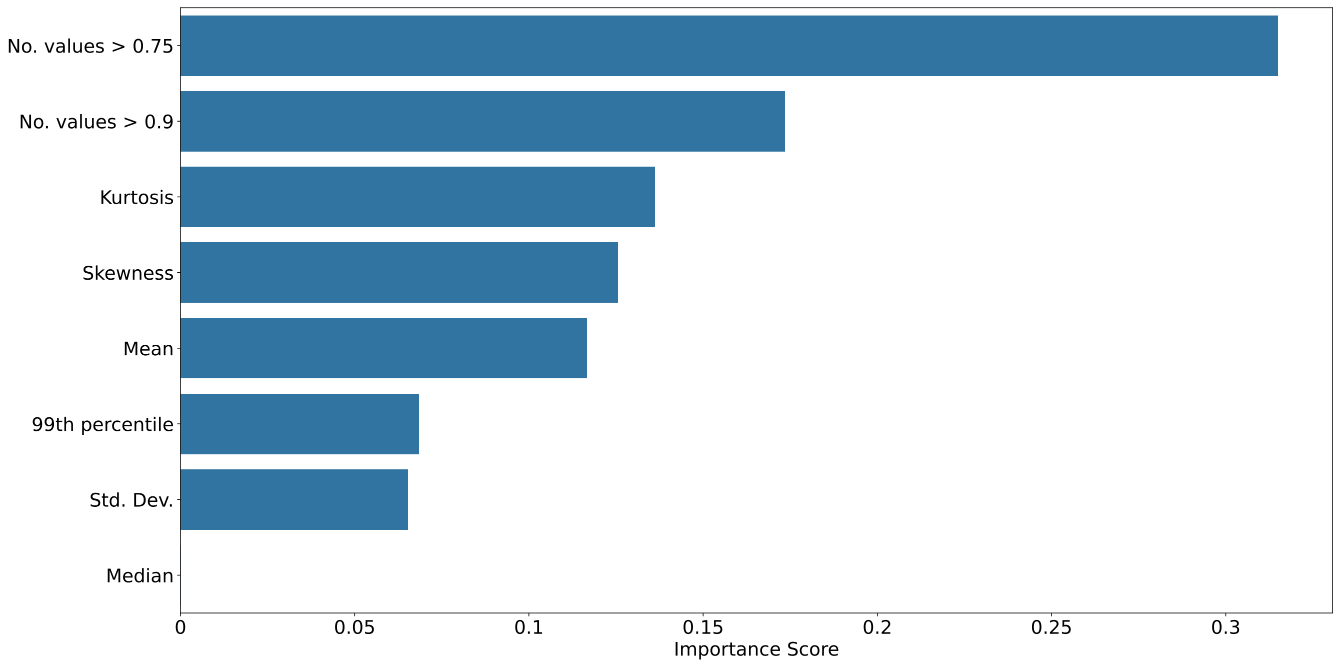

Figure S38: Feature importances from an XGBoost classifier distinguishing clean vs. noisy input conditions collected from Recurrent ConvSNN trained on UCF11 dataset.

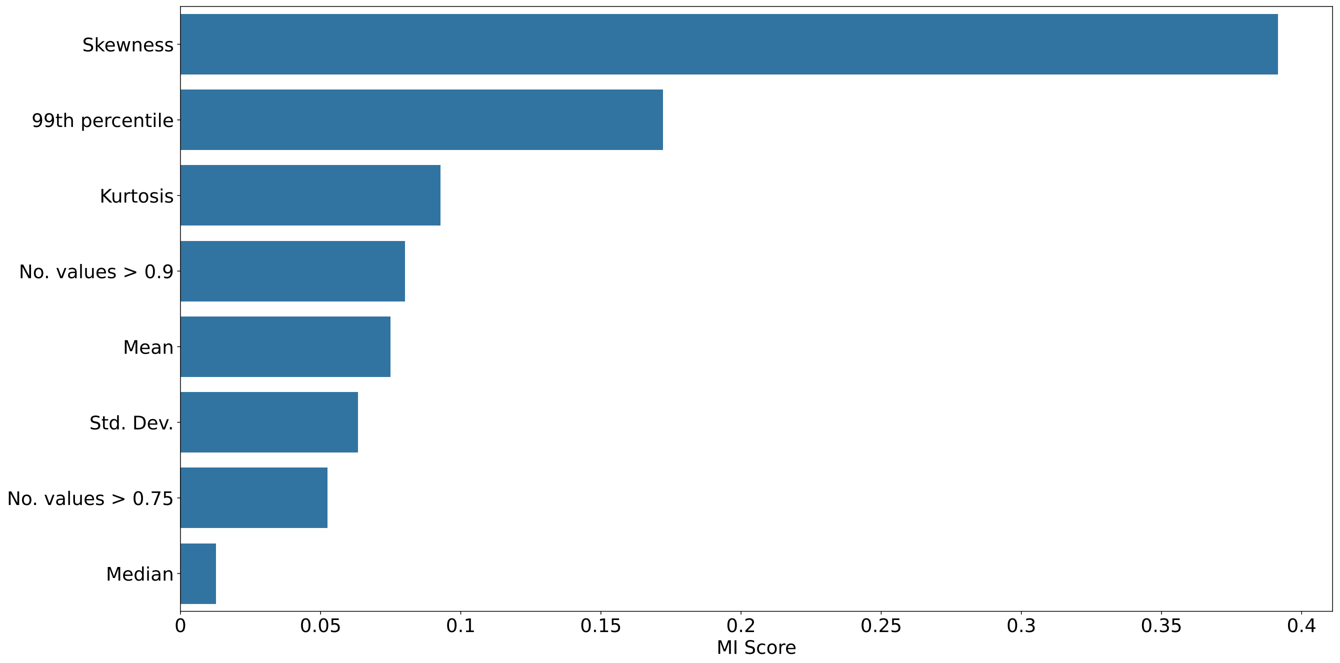

Figure S39: Mutual information scores between given statistic and clean vs. noisy input conditions collected from SpikingResnet18 trained on UCF11 dataset.

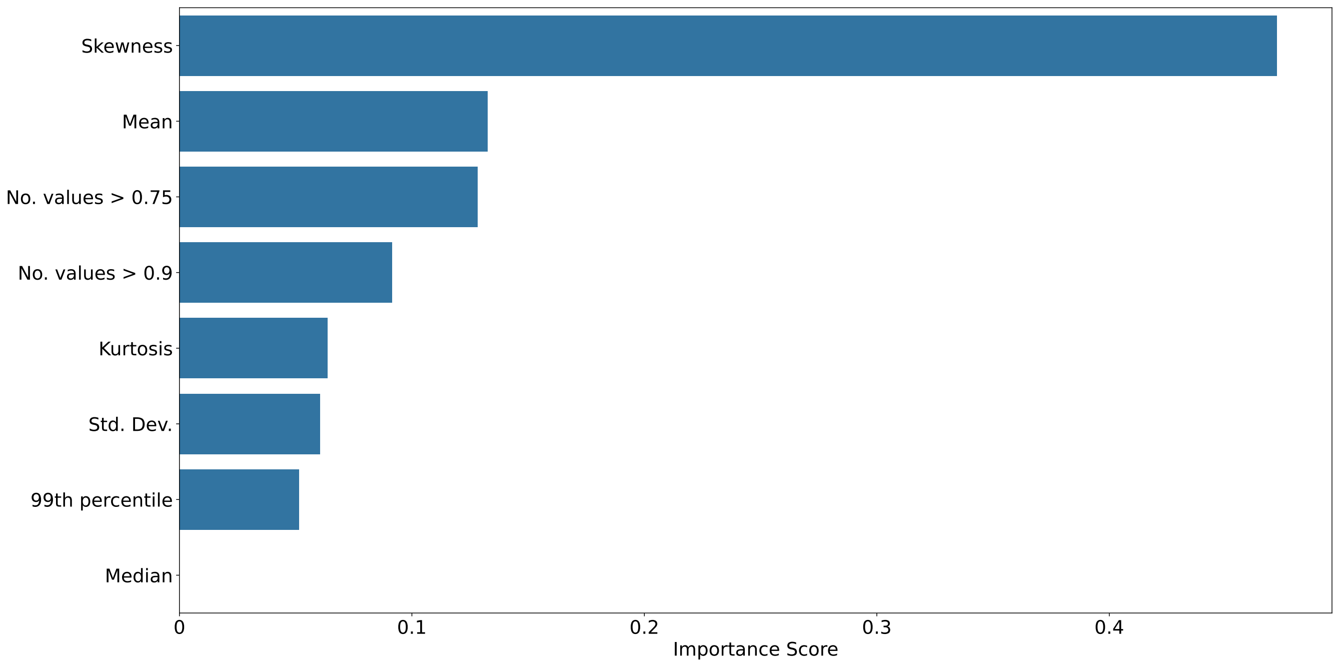

Figure S40: Feature importances from an XGBoost classifier distinguishing clean vs. noisy input conditions collected from SpikingResnet18 trained on UCF11 dataset.

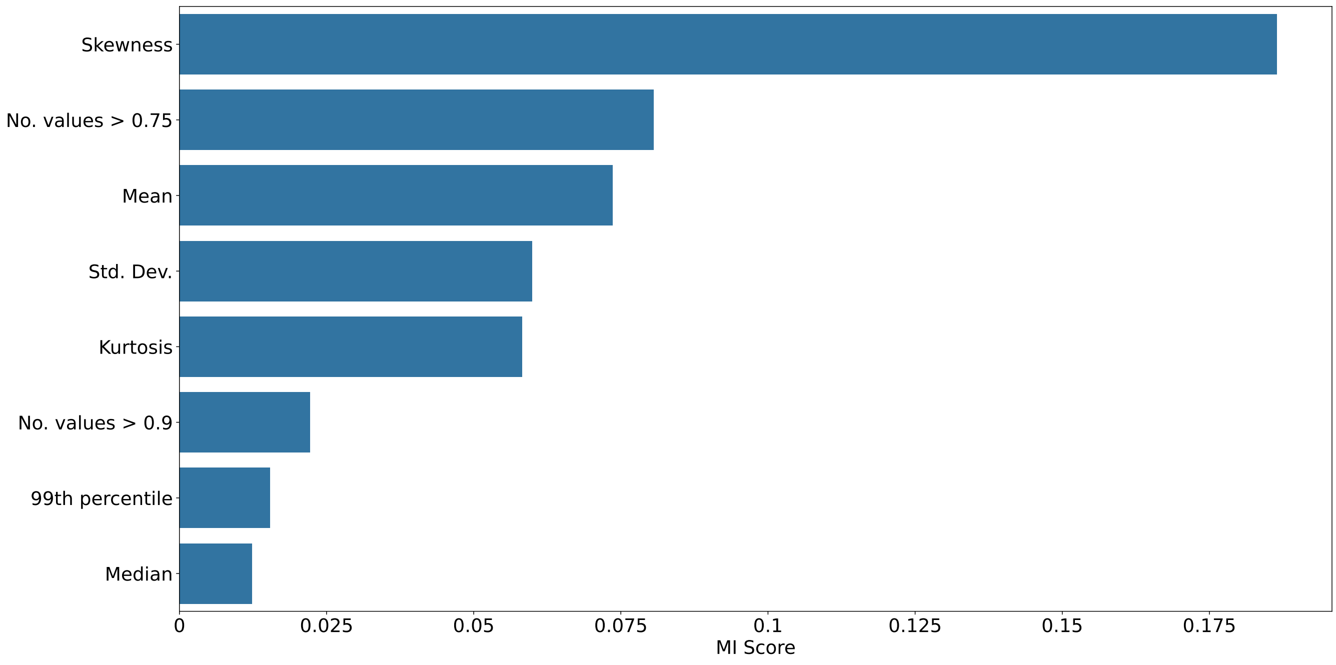

Figure S41: Mutual information scores between given statistic and clean vs. noisy input conditions collected from SpikingVGG11 trained on UCF11 dataset.

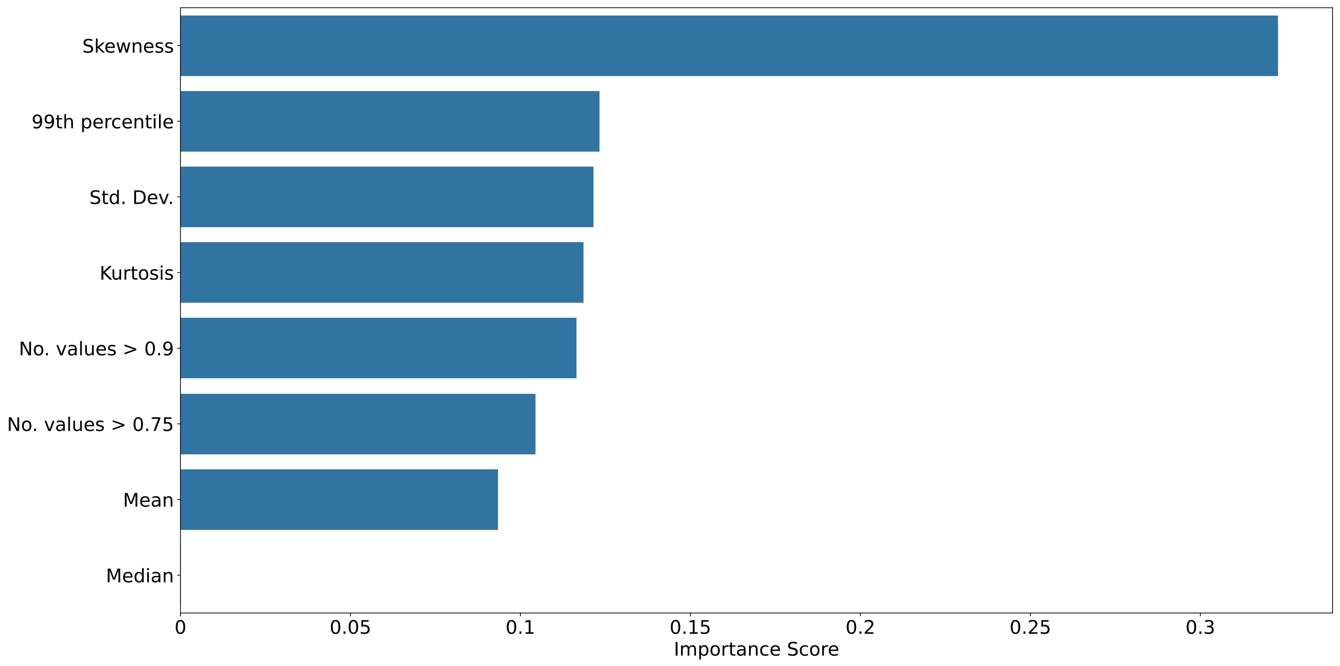

Figure S42: Feature importances from an XGBoost classifier distinguishing clean vs. noisy input conditions collected from SpikingVGG11 trained on UCF11 dataset.

Table S1. XGBoost 10-Fold Cross-Validation Accuracy with mean value ± SEM for each evaluated dataset-model combination.

Dataset	Experiment	F1	F2	F3	F4	F5	F6	F7	F8	F9	F10	Mean
**MNIST**	MLP-SNN	96.75	95.00	97.00	97.75	97.25	97.25	97.50	96.75	97.25	96.25	96.88 ± 0.25
	ConvSNN	91.50	90.75	87.25	85.00	88.75	86.25	84.50	88.25	87.00	87.50	87.68 ± 0.71
	Recurrent MLP-SNN	95.61	97.15	98.25	95.39	94.74	95.82	96.04	95.82	95.60	96.92	96.14 ± 0.32
	Recurrent ConvSNN	83.00	87.25	84.00	83.50	84.00	88.00	84.75	86.75	85.00	86.25	85.25 ± 0.54
	SpikingResnet18	96.79	98.13	97.59	96.51	96.78	97.05	97.32	96.78	98.39	98.12	97.35 ± 0.21
	Spiking VGG	76.75	78.00	80.75	80.00	84.75	81.00	81.75	80.25	78.50	80.25	80.20 ± 0.70
**CIFAR-10**	MLP-SNN	76.00	74.75	73.50	68.50	78.50	79.25	76.75	72.00	73.25	71.25	74.38 ± 1.06
	ConvSNN	92.50	93.50	92.25	90.50	93.00	94.25	93.00	91.50	93.75	91.50	92.58 ± 0.37
	Recurrent MLP-SNN	97.98	97.98	97.76	97.76	97.31	98.43	99.55	96.86	98.43	99.33	98.14 ± 0.26
	Recurrent ConvSNN	84.25	86.75	86.50	85.00	84.25	85.25	87.25	86.25	84.75	85.75	85.60 ± 0.34
	SpikingResnet18	92.50	93.50	92.25	93.00	92.50	91.75	91.50	93.50	93.25	91.00	92.48 ± 0.27
	SpikingVGG	89.50	90.50	88.75	91.75	89.25	92.00	89.75	91.50	87.75	88.75	89.95 ± 0.45
**EventMNIST**	MLP-SNN	94.50	92.25	92.25	94.00	95.25	94.00	92.50	93.00	95.25	92.75	93.57 ± 0.37
	ConvSNN	90.25	87.75	86.00	88.50	87.00	87.00	88.00	82.75	85.00	87.50	86.98 ± 0.65
	Recurrent MLP-SNN	74.75	76.25	76.00	78.00	77.25	77.00	79.25	76.25	76.00	75.50	76.62 ± 0.41
	Recurrent ConvSNN	70.75	75.25	75.25	73.50	74.50	75.50	74.50	77.25	74.25	74.75	74.55 ± 0.53
	SpikingResnet18	78.50	77.75	81.00	79.75	79.00	83.75	84.50	84.00	83.25	84.00	81.55 ± 0.83
	SpikingVGG	82.75	81.50	82.75	83.25	80.00	81.75	81.25	81.00	83.50	82.25	82.00 ± 0.35
**UCF11**	MLP-SNN	67.86	57.14	77.78	77.78	55.56	51.85	77.78	66.67	55.56	70.37	65.83 ± 3.22
	ConvSNN	69.23	84.62	91.67	91.67	91.67	83.33	91.67	75.00	83.33	83.33	84.55 ± 2.43
	Recurrent MLP-SNN	95.83	91.67	100.00	95.83	83.33	100.00	95.83	86.96	95.65	86.96	93.21 ± 1.82
	Recurrent ConvSNN	81.82	81.82	72.73	77.27	86.36	77.27	81.82	68.18	76.19	71.43	77.49 ± 1.77
	SpikingResnet18	96.00	92.00	100.00	92.00	96.00	88.00	87.50	100.00	83.33	95.83	93.07 ± 1.75
	SpikingVGG	93.33	66.67	86.67	66.67	80.00	80.00	80.00	66.67	78.57	71.43	77.00 ± 2.87
