# Supplementary material for: Operational manifolds in spiking neural networks
Source: Front Neurosci. 2026 Feb 18;20:1755119. doi: 10.3389/fnins.2026.1755119 (PMC12956522; doi:10.3389/fnins.2026.1755119)
Supplement: Supplementary file 3 [file Data_Sheet_3.pdf]

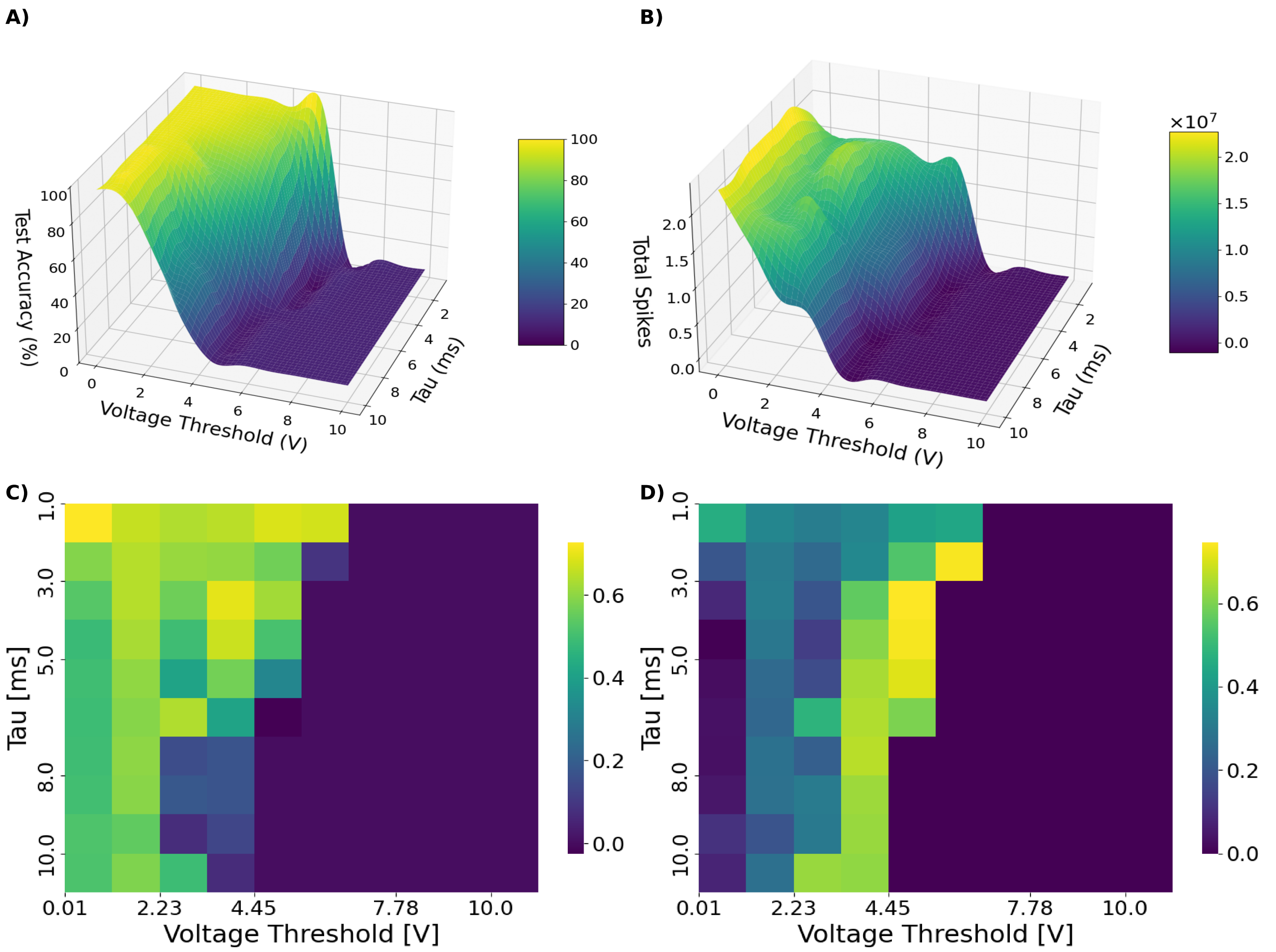

Figure S6: Heatmaps over (τ_m, V_{th}) showing **(A)** test accuracy, **(B)** total number of spikes during inference and **(C-D)** the efficiency metrics BES ($\lambda = 0.5$) and EAS ($\beta = 5$) for the MLPSNN trained on MNIST.

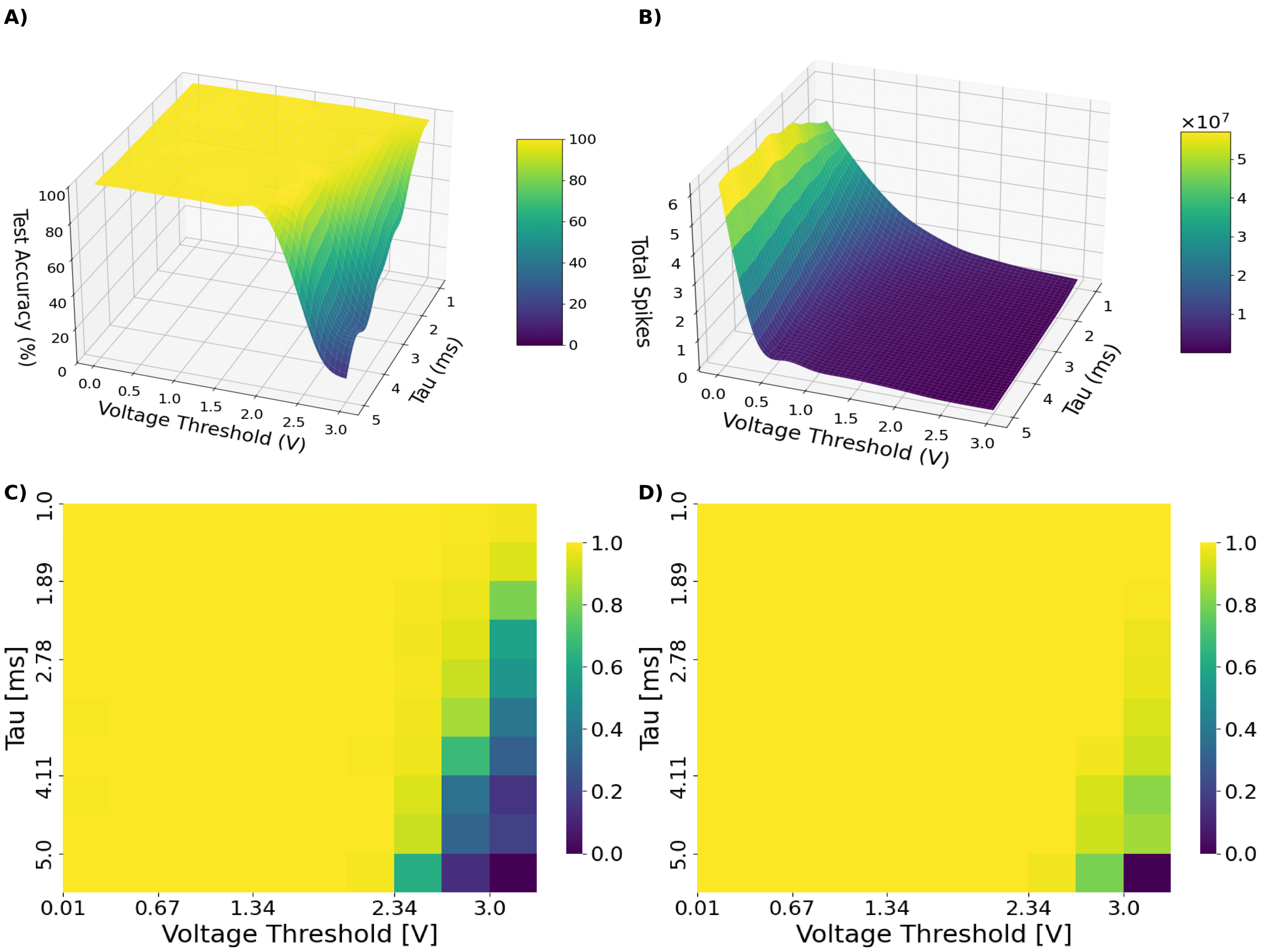

Figure S7: Heatmaps over (τ_m, V_{th}) showing **(A)** test accuracy, **(B)** total number of spikes during inference and **(C-D)** the efficiency metrics BES ($\lambda = 0.5$) and EAS ($\beta = 5$) for the SpikingResnet18 trained on MNIST.

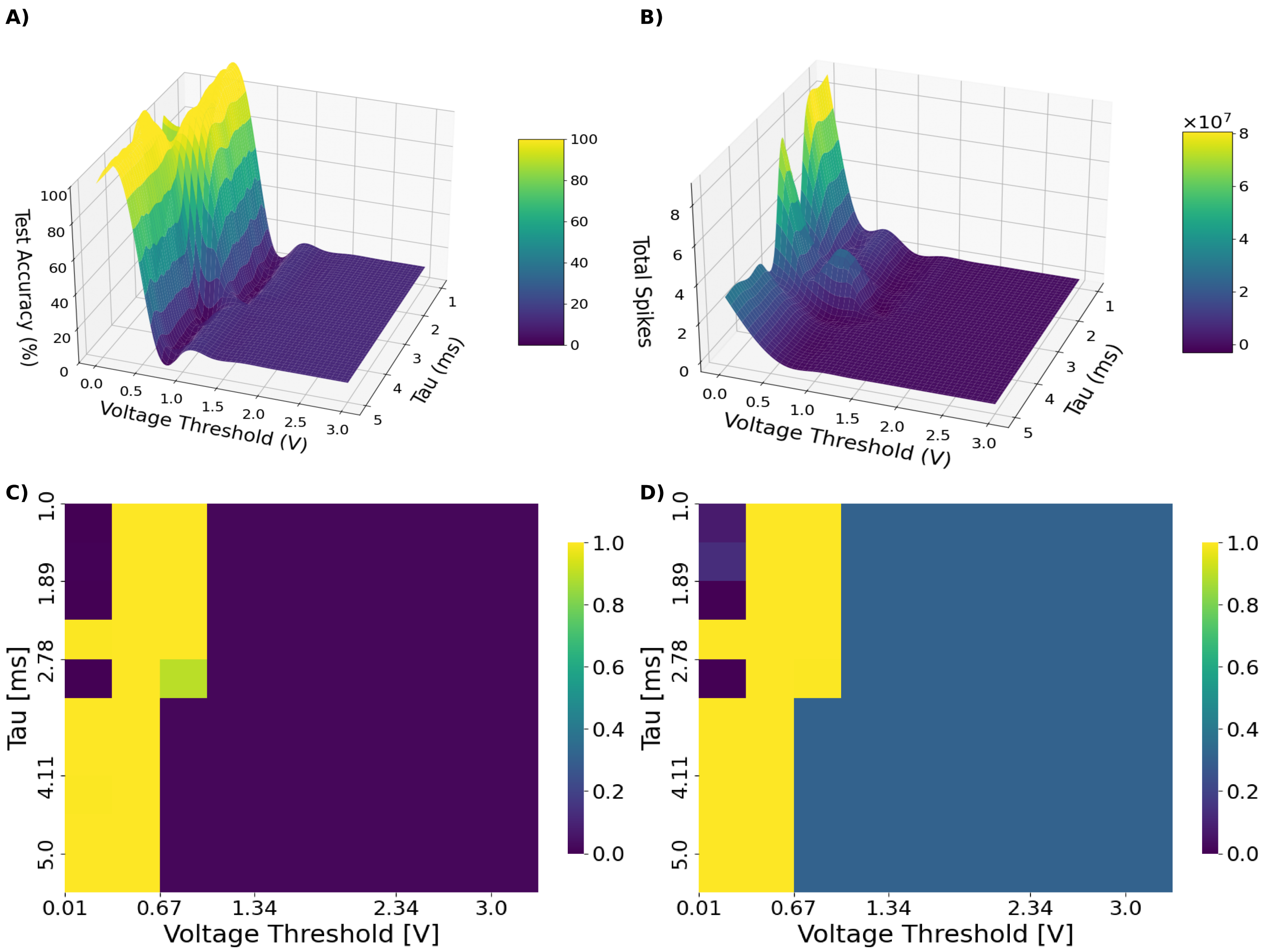

Figure S8: Heatmaps over $(\tau_m, V_{\mathrm{th}})$ showing **(A)** test accuracy, **(B)** total number of spikes during inference and **(C-D)** the efficiency metrics BES $(\lambda = 0.5)$ and EAS $(\beta = 5)$ for the VGG-11 trained on MNIST.

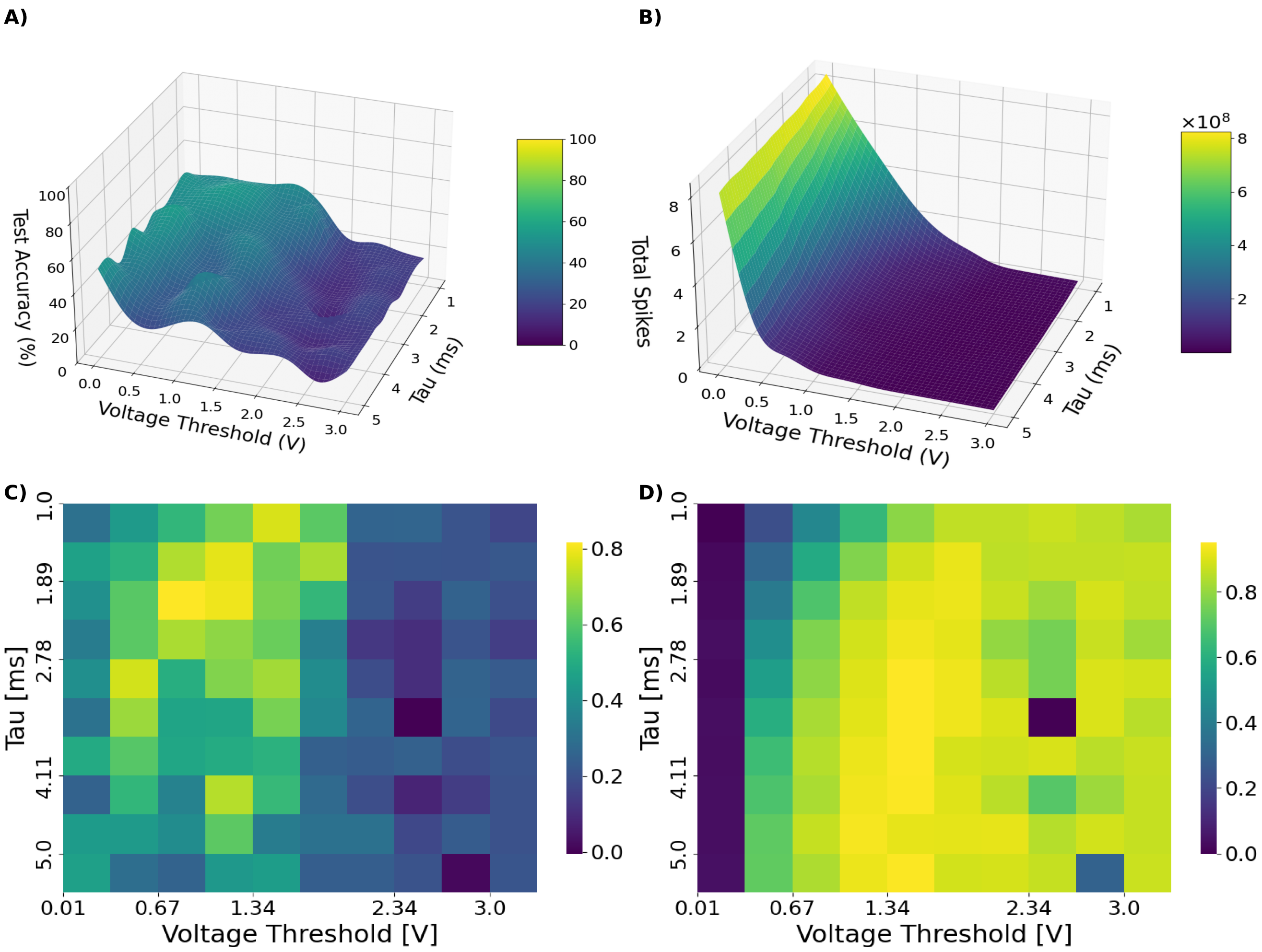

Figure S9: Heatmaps over (τ_m, V_{th}) showing **(A)** test accuracy, **(B)** total number of spikes during inference and **(C-D)** the efficiency metrics BES ($\lambda = 0.5$) and EAS ($\beta = 5$) for the SpikingResnet18 trained on UCF11.

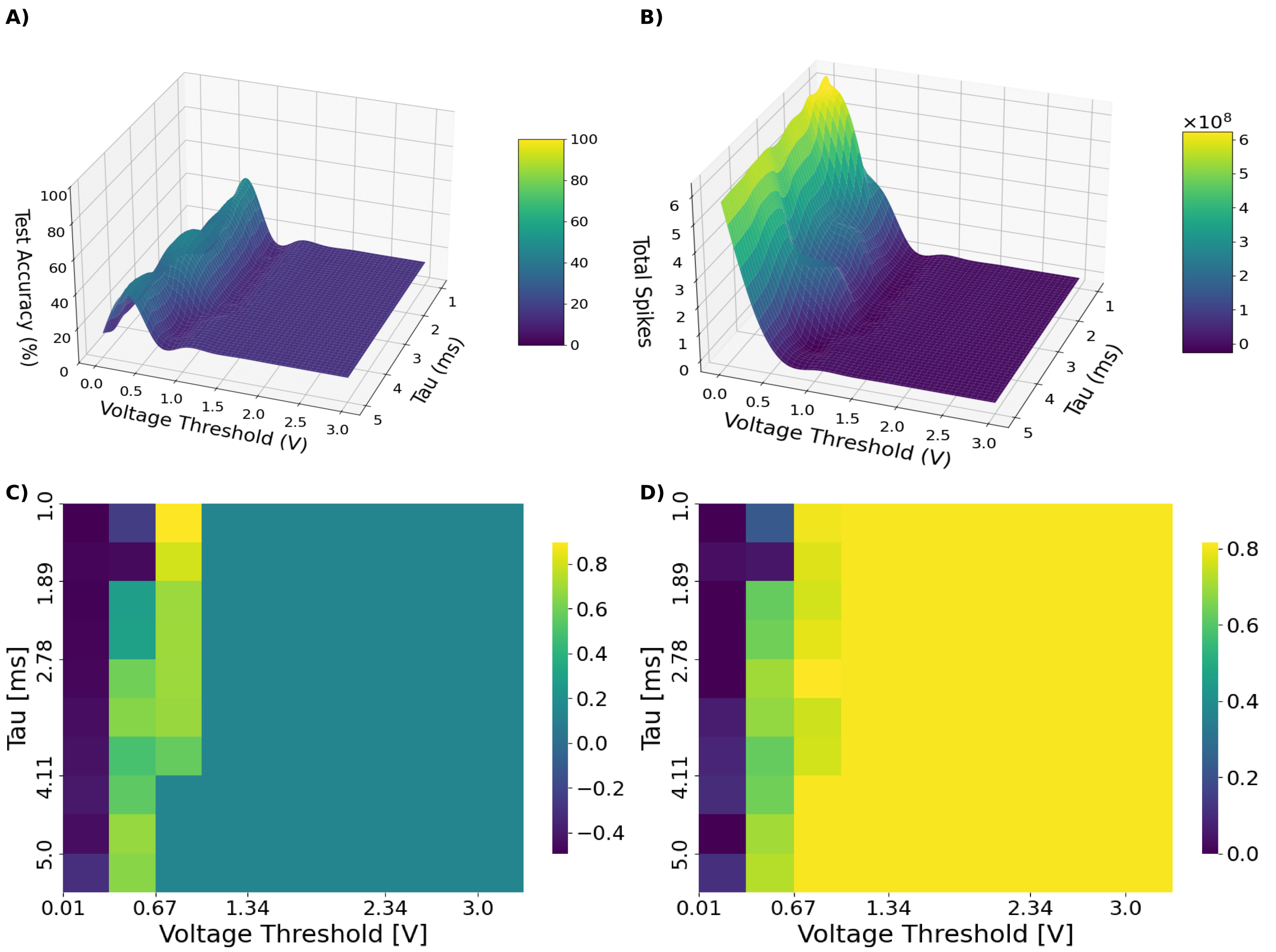

Figure S10: Heatmaps over (τ_m, V_{th}) showing **(A)** test accuracy, **(B)** total number of spikes during inference and **(C-D)** the efficiency metrics BES $(\lambda = 0.5)$ and EAS $(\beta = 5)$ for the VGG-11 trained on UCF11.
