# Supplementary material for: Operational manifolds in spiking neural networks
Source: Front Neurosci. 2026 Feb 18;20:1755119. doi: 10.3389/fnins.2026.1755119 (PMC12956522; doi:10.3389/fnins.2026.1755119)
Supplement: Supplementary file 4 [file Data_Sheet_4.pdf]

Supplementary Material

1 CORRELATION MATRICES AND FEATURE PREDICTIVE POWER ANALYSIS UNDER INPUT NOISE CONDITIONS

In Figures S1-S21, the average correlation analysis are shown for all analyzed architectures and dataset combinations except the ConvSNN and SpikingResnet18 trained on MNIST dataset, which is included in the main manuscript.

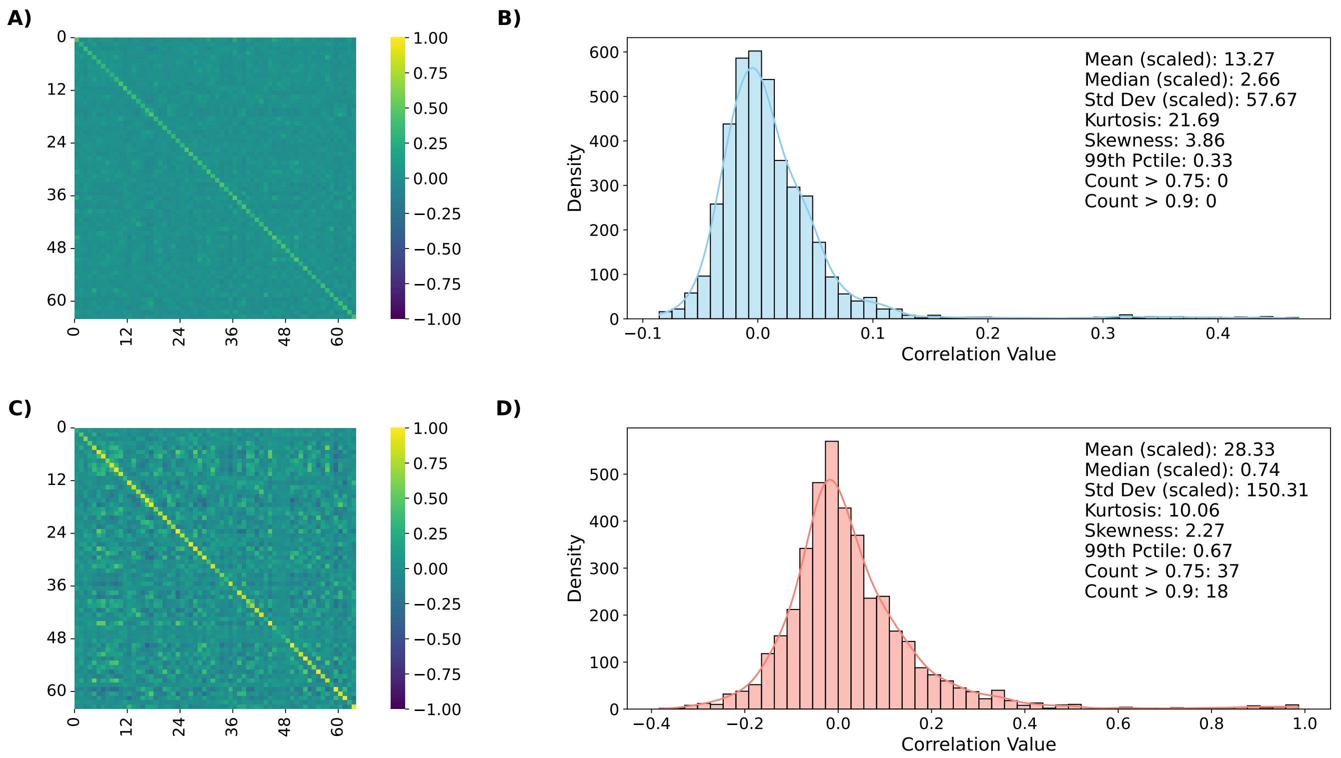

Figure S1: Average spike-train correlation matrices and their distributions for clean (**A,B**) and noisy (**C,D**) inputs for MLP-SNN trained on MNIST dataset.

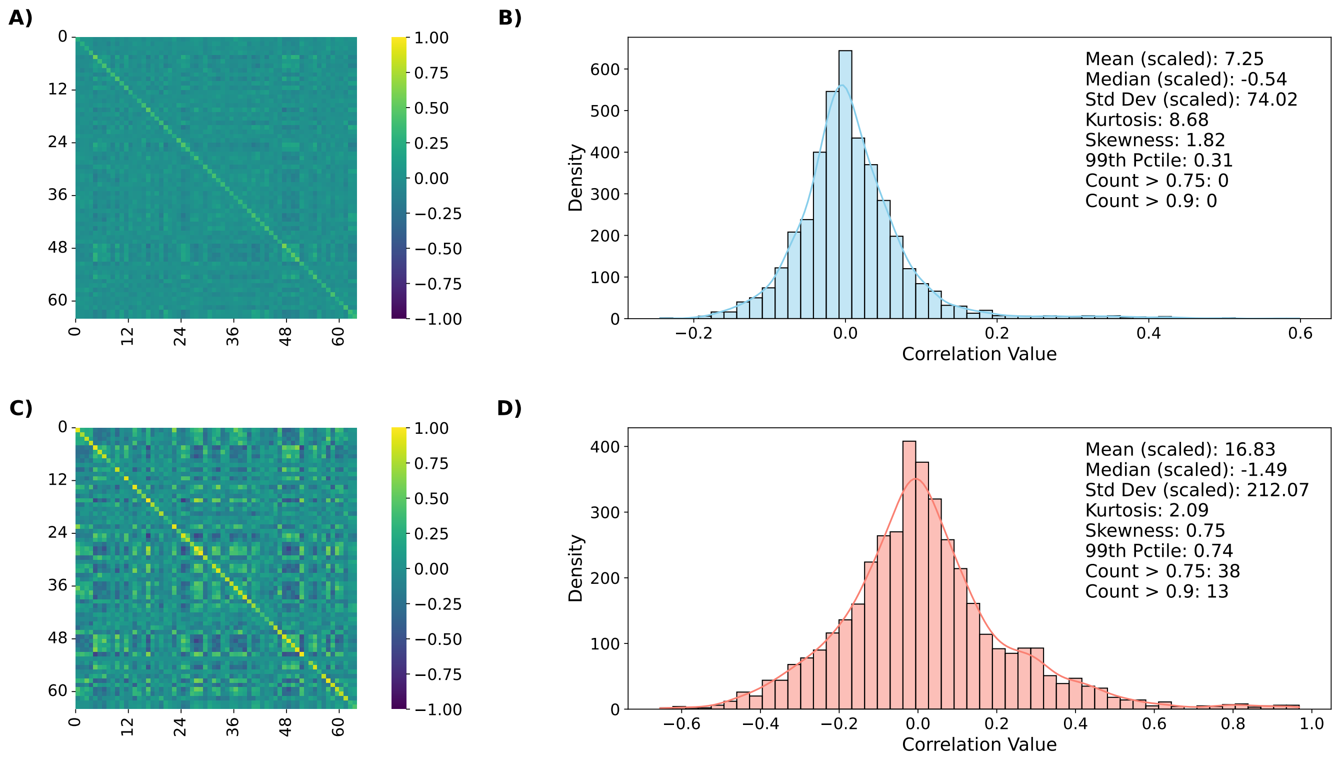

Figure S2: Average spike-train correlation matrices and their distributions for clean (**A,B**) and noisy (**C,D**) inputs for Recurrent MLP-SNN trained on MNIST dataset.

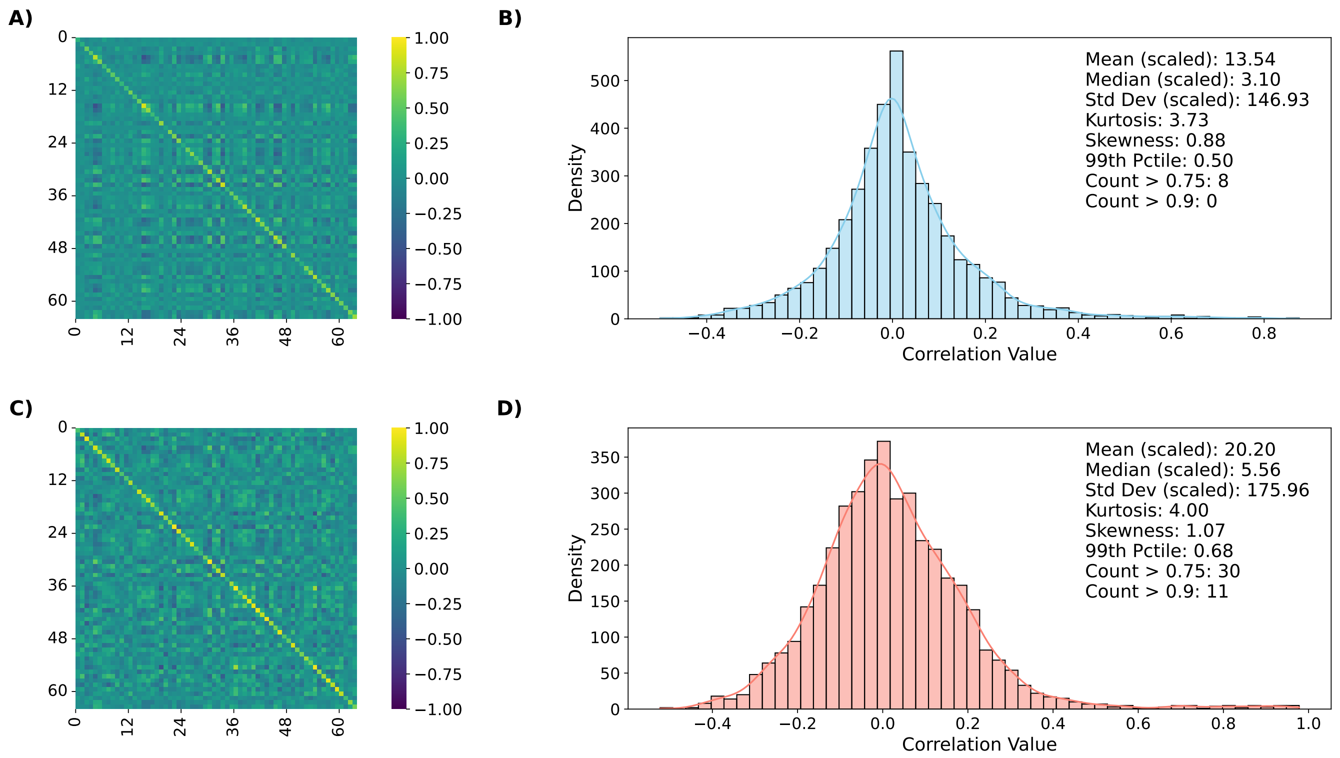

Figure S3: Average spike-train correlation matrices and their distributions for clean (**A,B**) and noisy (**C,D**) inputs for Recurrent ConvSNN trained on MNIST dataset.

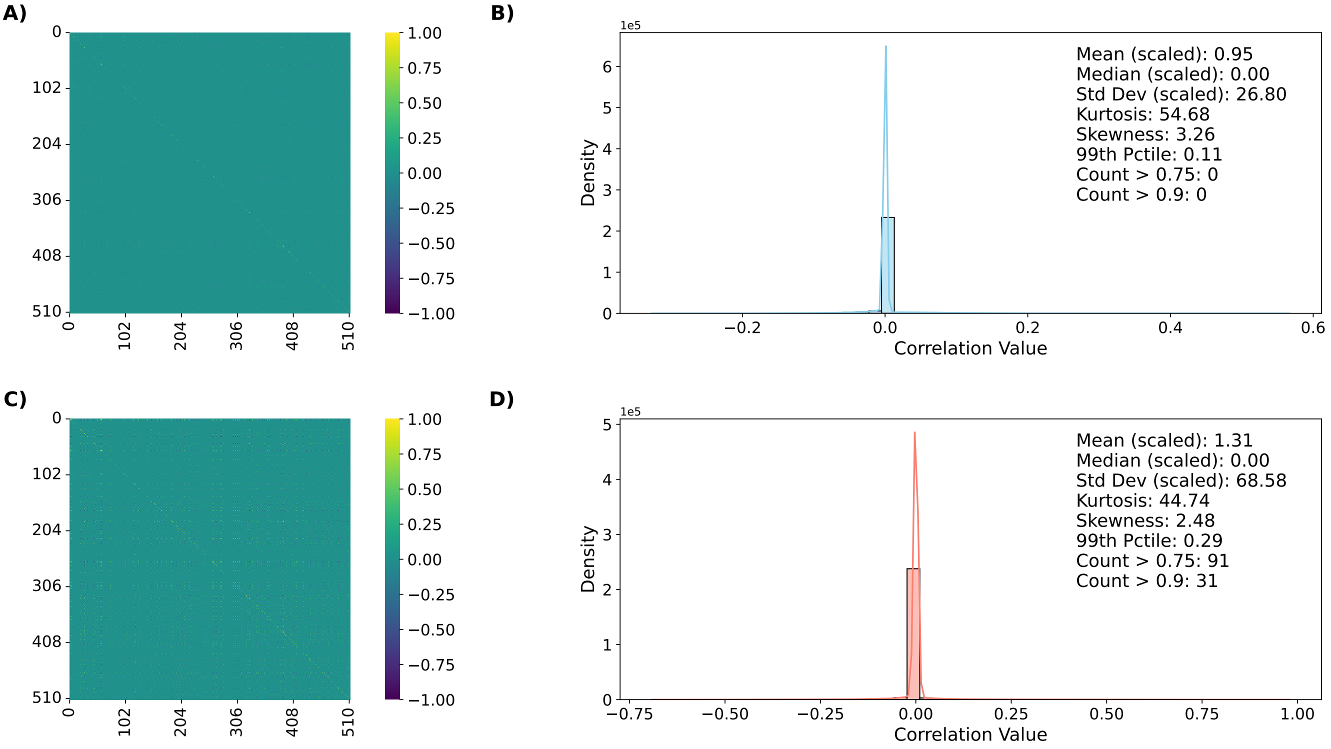

Figure S4: Average spike-train correlation matrices and their distributions for clean (**A,B**) and noisy (**C,D**) inputs for SpikingVGG11 trained on MNIST dataset.

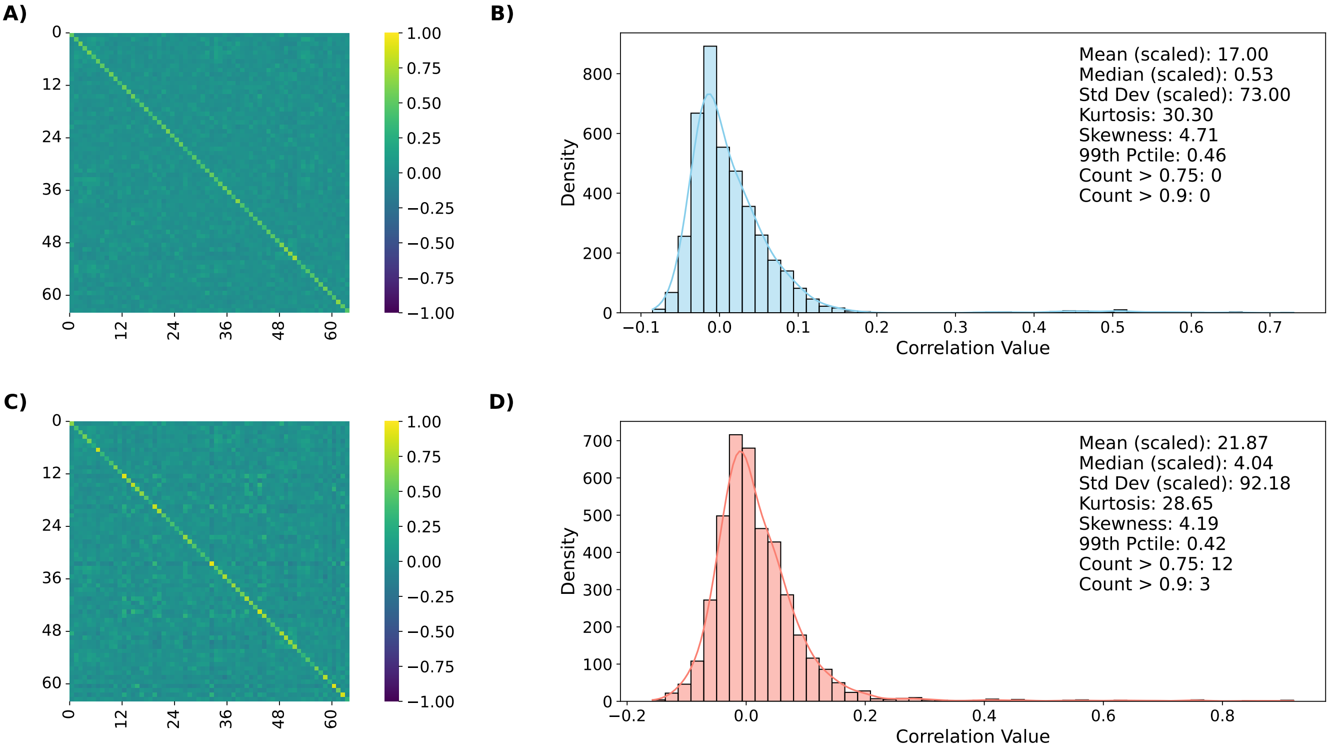

Figure S5: Average spike-train correlation matrices and their distributions for clean (**A,B**) and noisy (**C,D**) inputs for MLP-SNN trained on CIFAR-10 dataset.

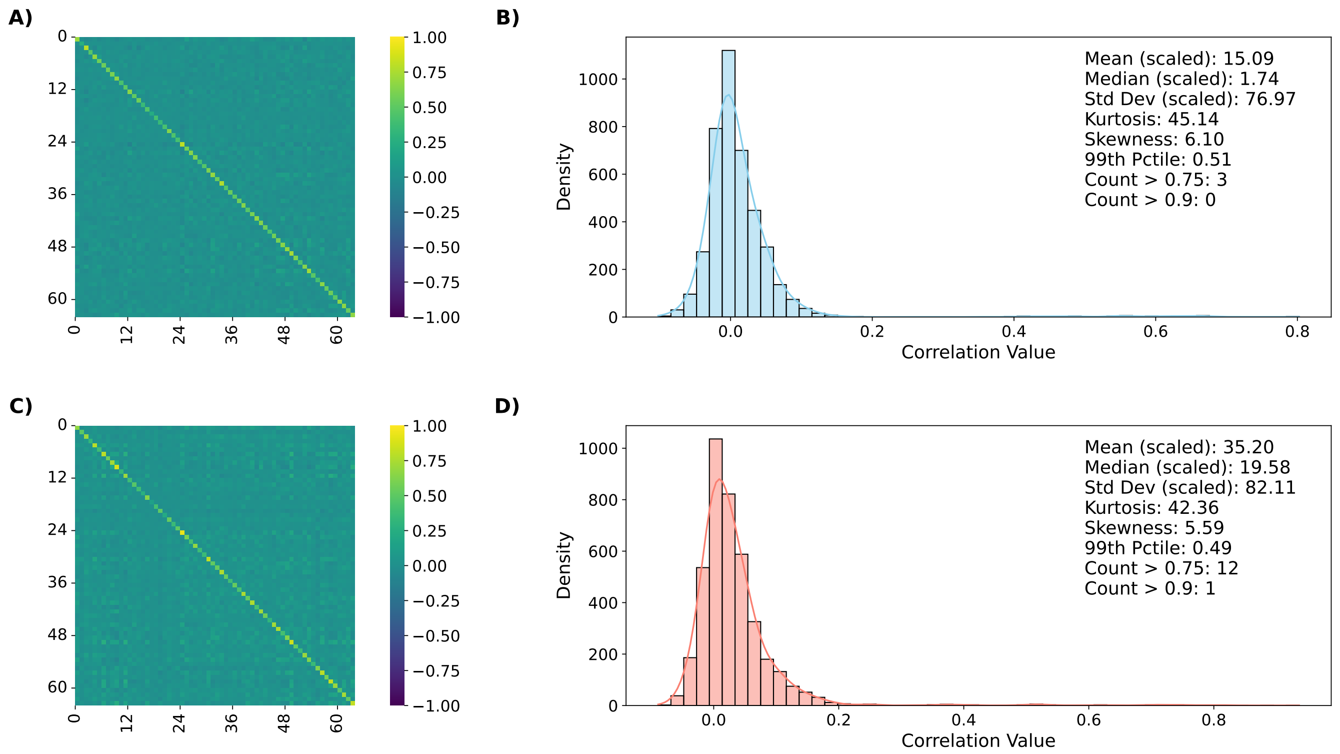

Figure S6: Average spike-train correlation matrices and their distributions for clean (**A,B**) and noisy (**C,D**) inputs for ConvSNN trained on CIFAR-10 dataset.

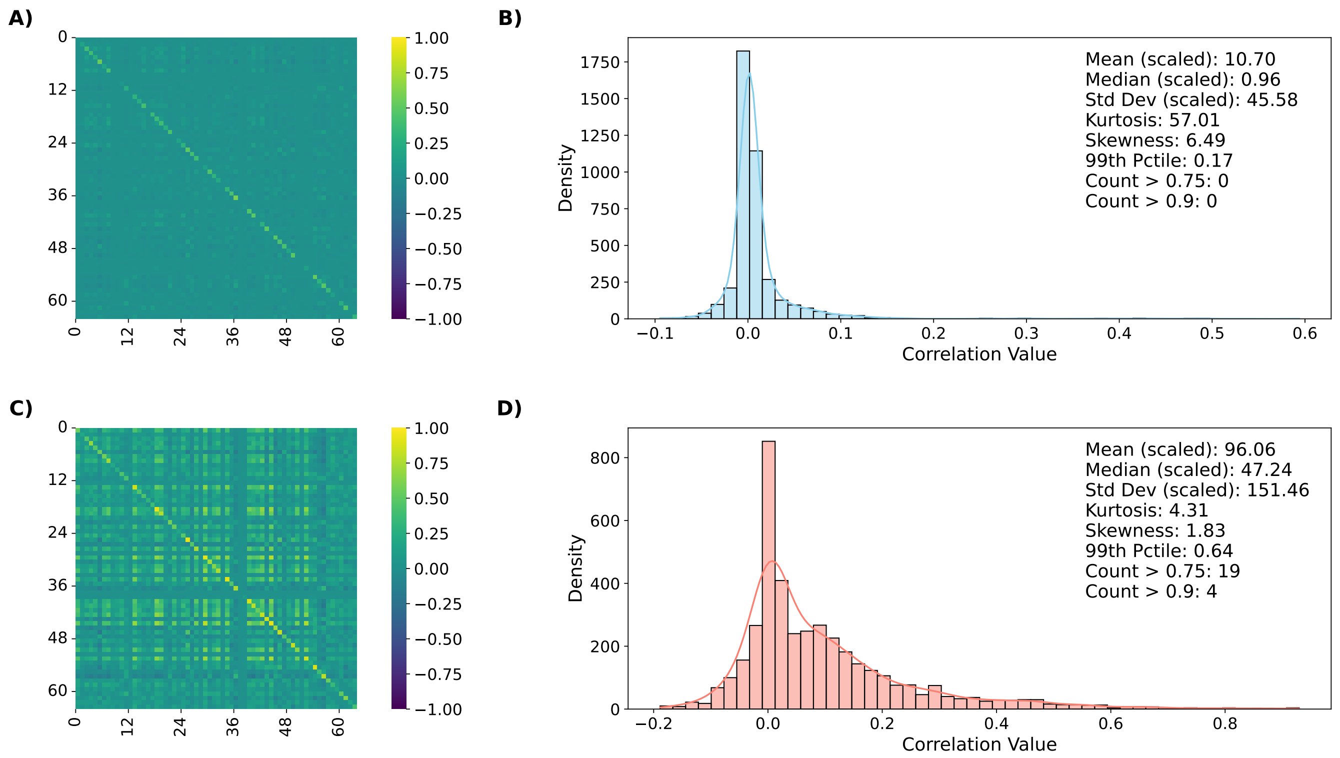

Figure S7: Average spike-train correlation matrices and their distributions for clean (**A,B**) and noisy (**C,D**) inputs for Recurrent MLP-SNN trained on CIFAR-10 dataset.
