# Supplementary material for: Operational manifolds in spiking neural networks
Source: Front Neurosci. 2026 Feb 18;20:1755119. doi: 10.3389/fnins.2026.1755119 (PMC12956522; doi:10.3389/fnins.2026.1755119)
Supplement: Supplementary file 5 [file Data_Sheet_5.pdf]

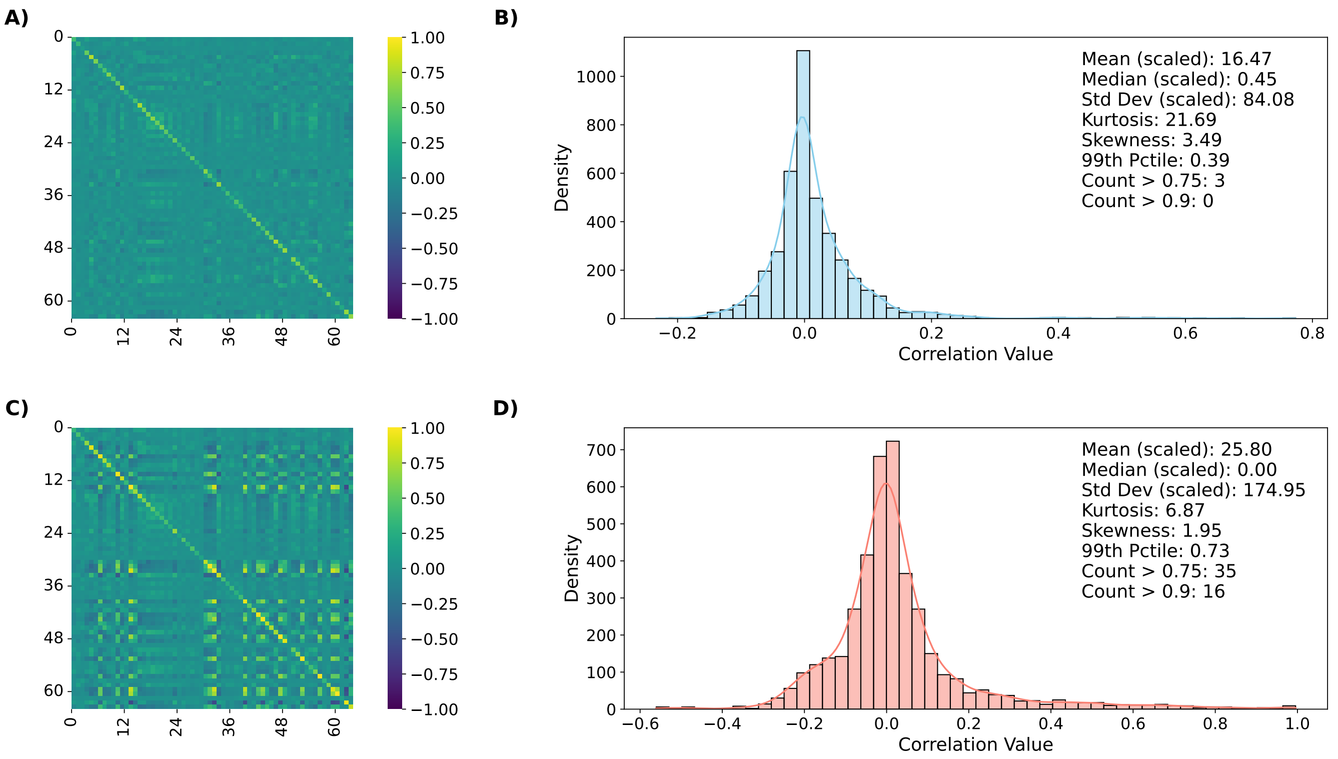


Figure S8: Average spike-train correlation matrices and their distributions for clean (**A,B**) and noisy (**C,D**) inputs for Recurrent ConvSNN trained on CIFAR-10 dataset.

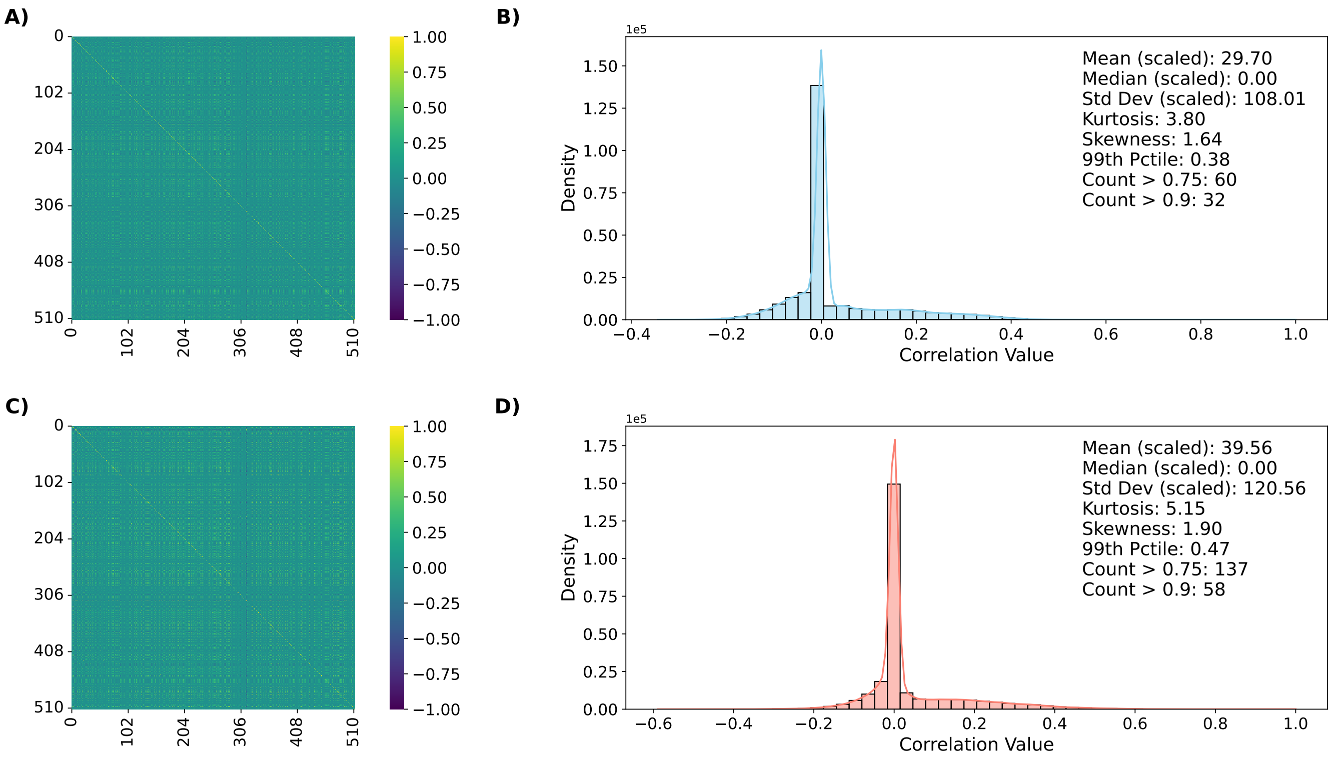


Figure S9: Average spike-train correlation matrices and their distributions for clean (**A,B**) and noisy (**C,D**) inputs for SpikingResnet18 trained on CIFAR-10 dataset.

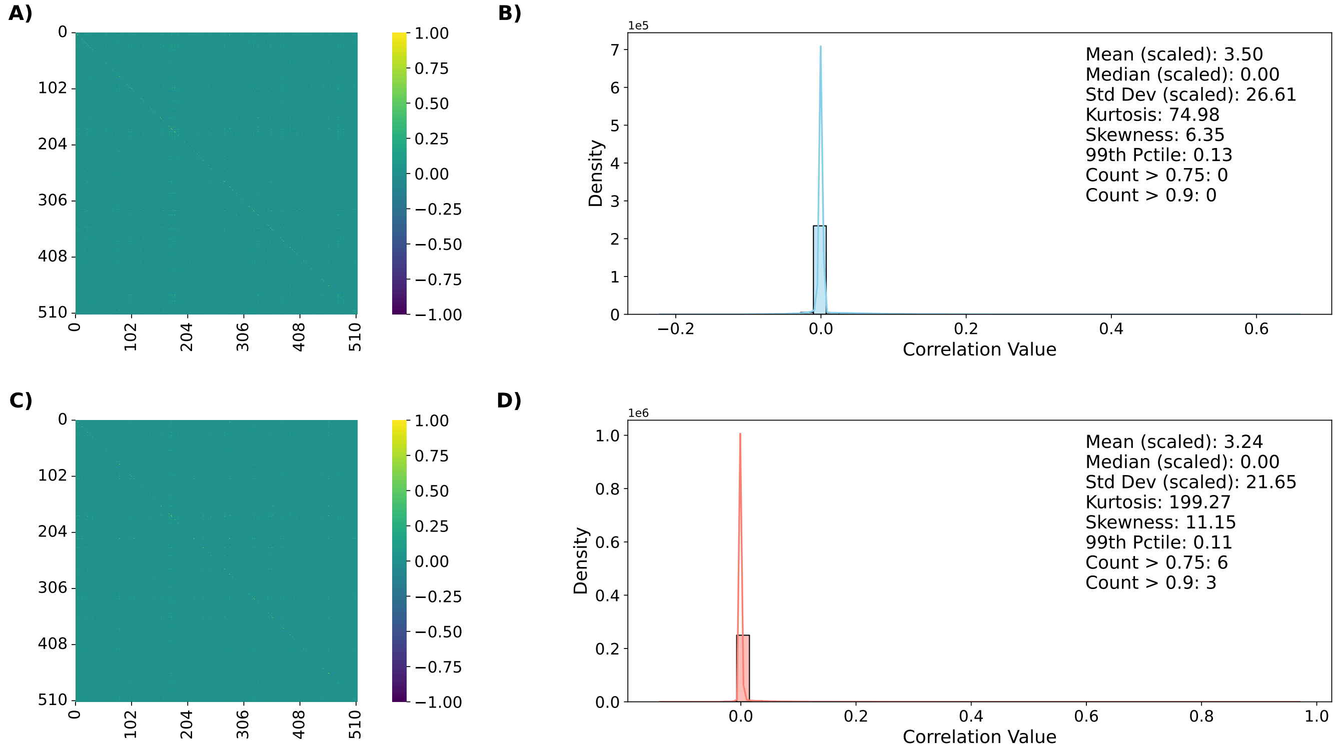


Figure S10: Average spike-train correlation matrices and their distributions for clean (**A,B**) and noisy (**C,D**) inputs for SpikingVGG11 trained on CIFAR-10 dataset.

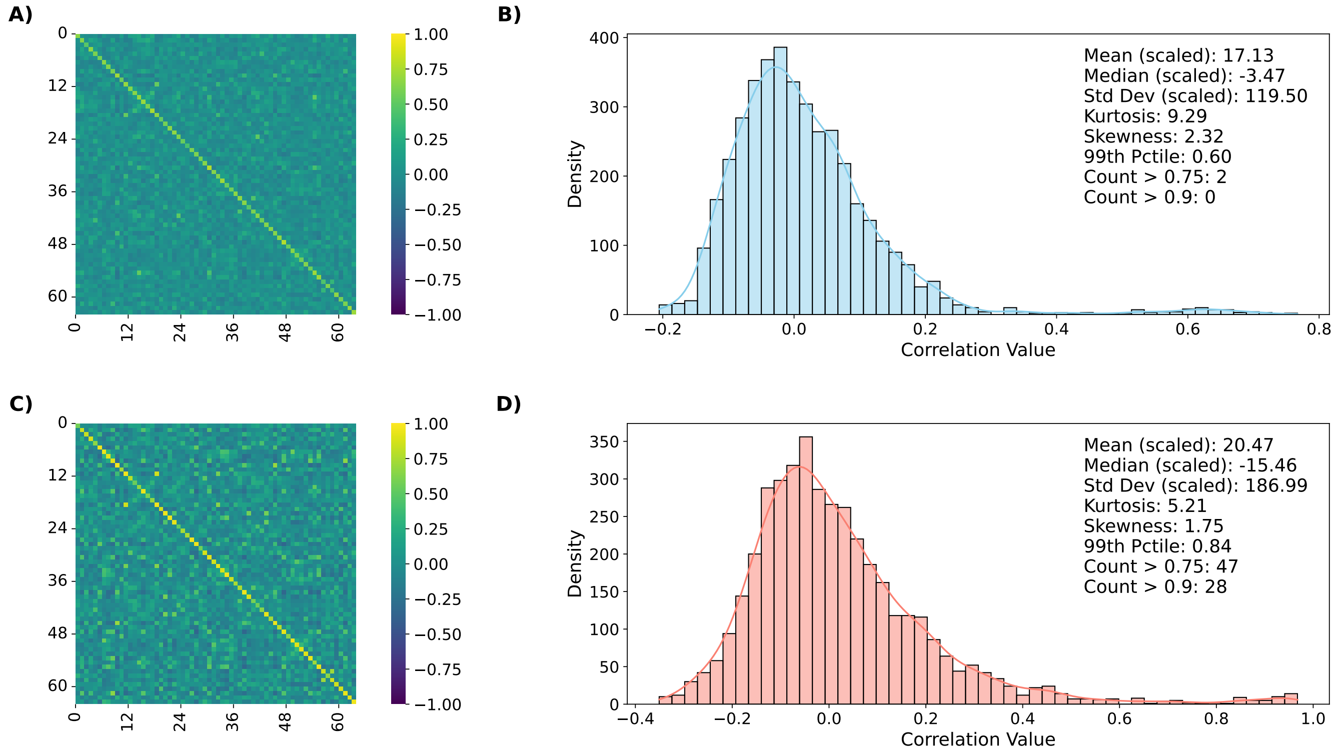


Figure S11: Average spike-train correlation matrices and their distributions for clean (**A,B**) and noisy (**C,D**) inputs for MLP-SNN trained on EventMNIST dataset.

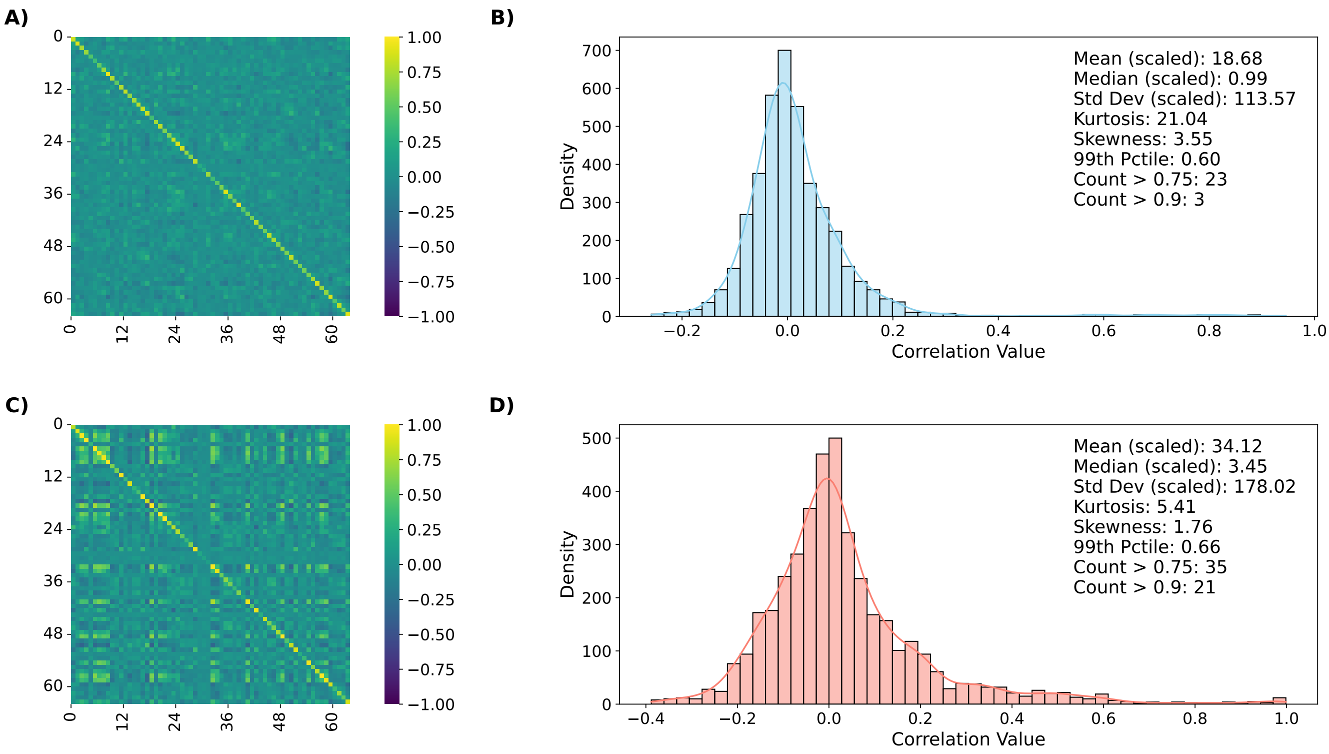


Figure S12: Average spike-train correlation matrices and their distributions for clean (**A,B**) and noisy (**C,D**) inputs for MLP-SNN trained on EventMNIST dataset.

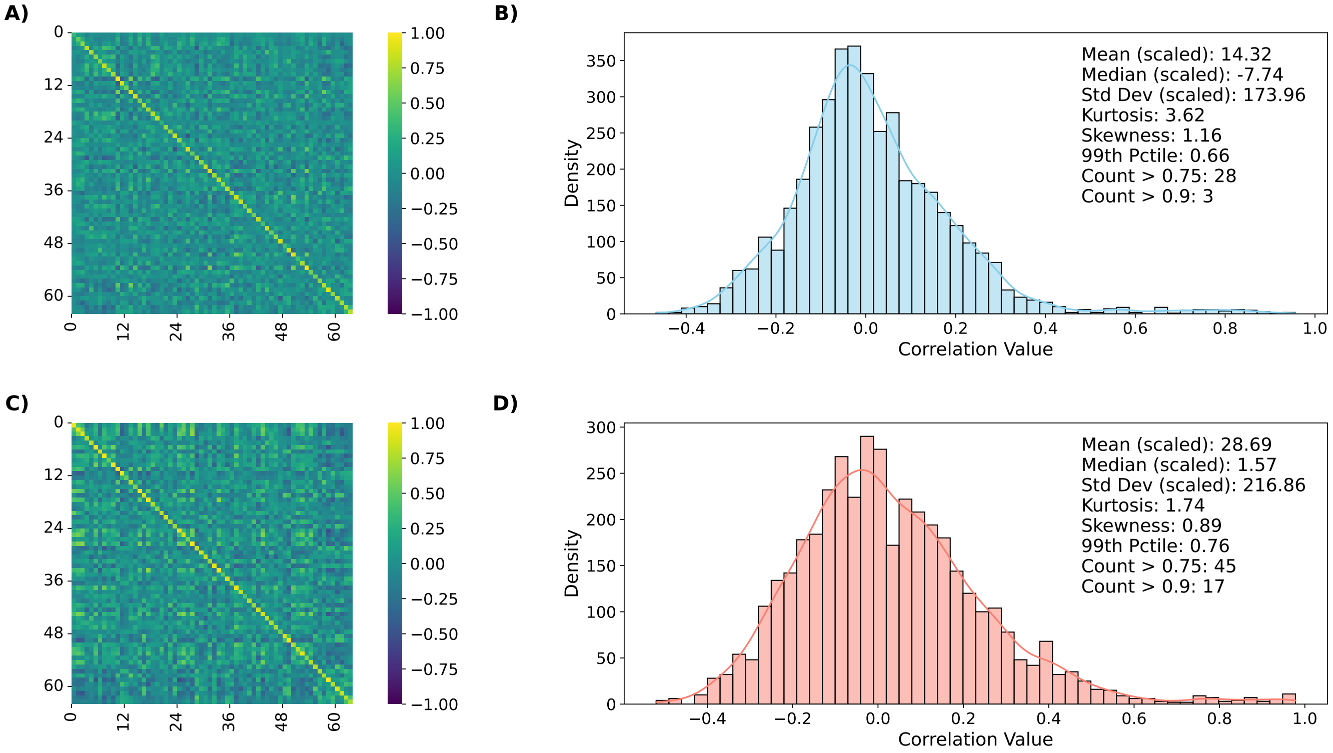


Figure S13: Average spike-train correlation matrices and their distributions for clean (**A,B**) and noisy (**C,D**) inputs for Recurrent MLP-SNN trained on EventMNIST dataset.

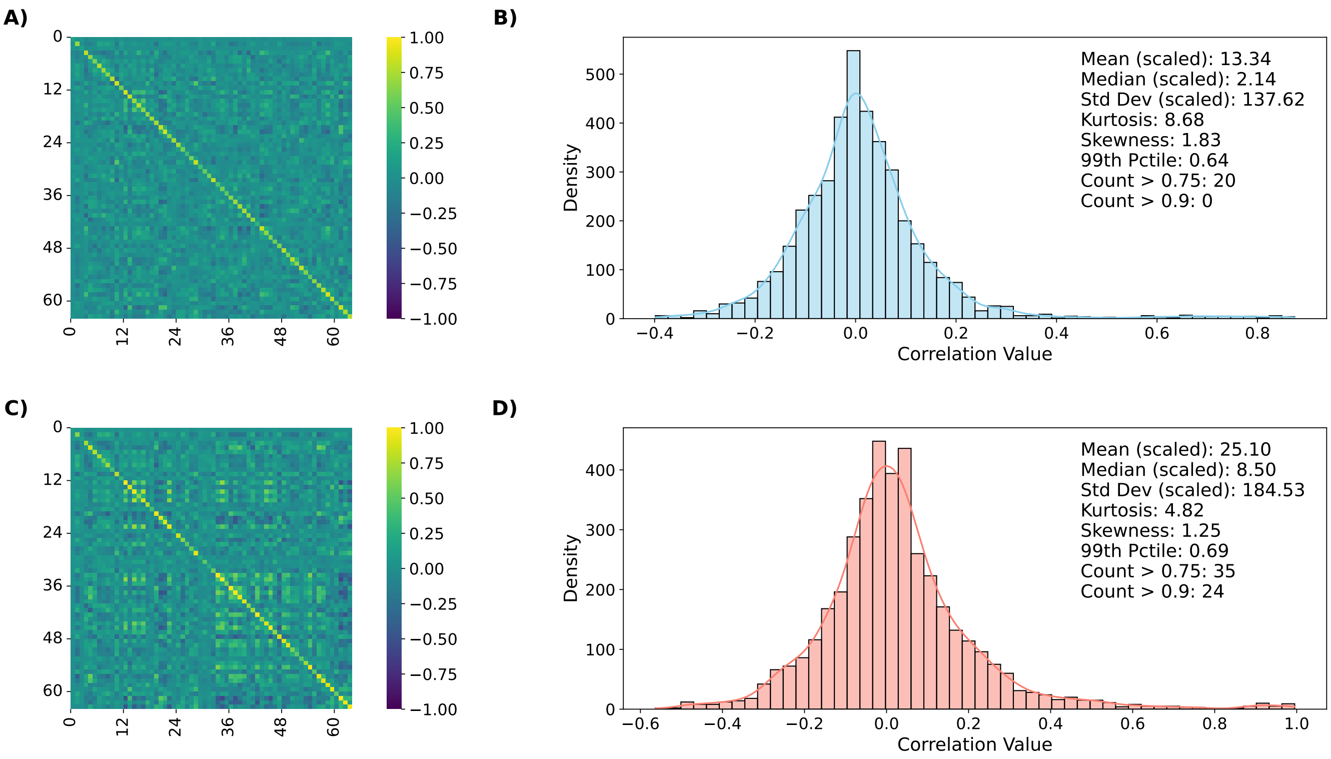


Figure S14: Average spike-train correlation matrices and their distributions for clean (**A,B**) and noisy (**C,D**) inputs for Recurrent ConvSNN trained on EventMNIST dataset.

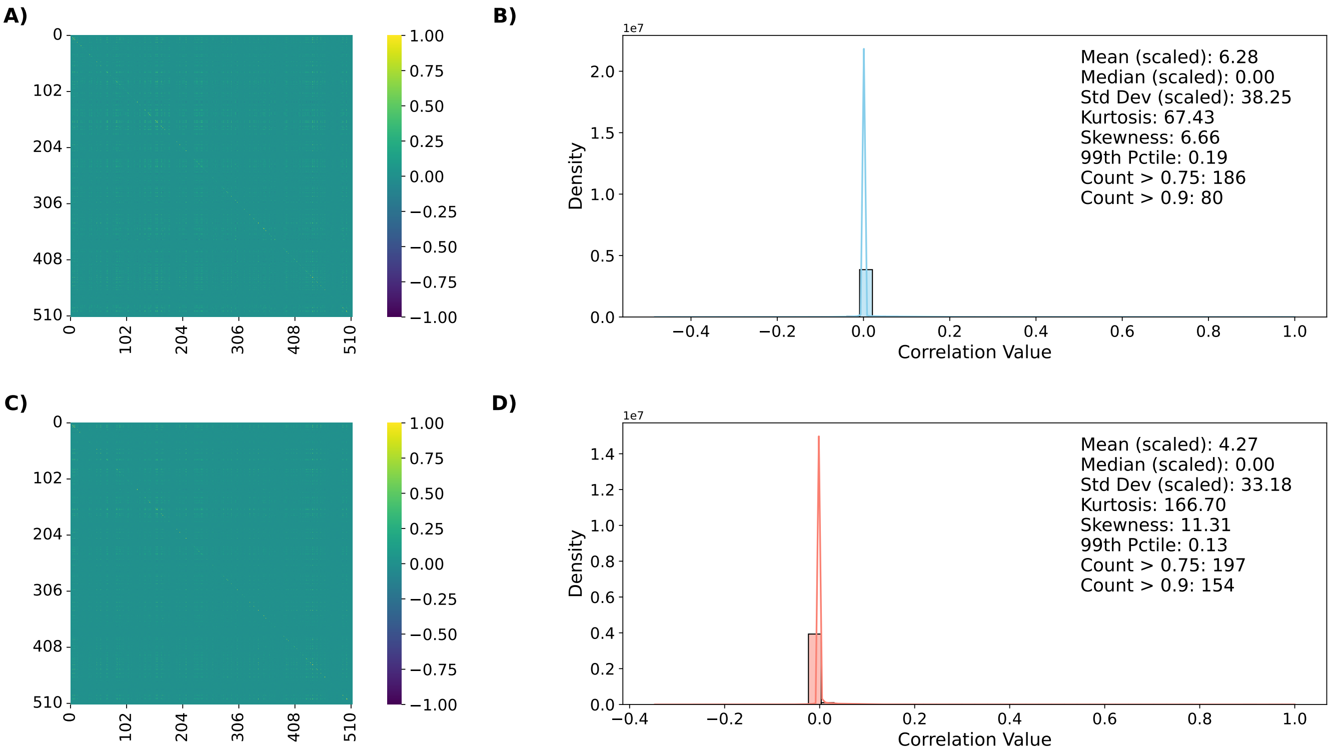


Figure S15: Average spike-train correlation matrices and their distributions for clean (**A,B**) and noisy (**C,D**) inputs for SpikingResnet18 trained on EventMNIST dataset.
