# Supplementary material for: Operational manifolds in spiking neural networks
Source: Front Neurosci. 2026 Feb 18;20:1755119. doi: 10.3389/fnins.2026.1755119 (PMC12956522; doi:10.3389/fnins.2026.1755119)
Supplement: Supplementary file 6 [file Data_Sheet_6.pdf]

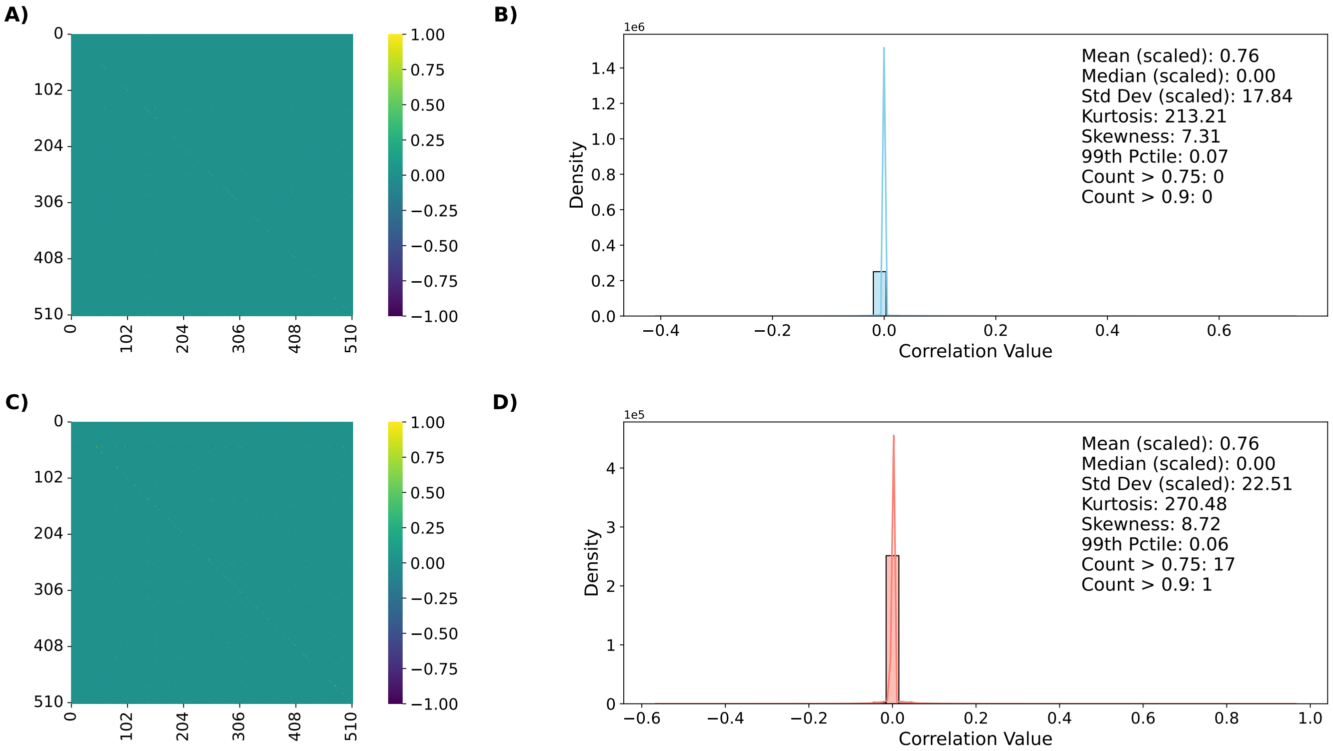

Figure S16: Average spike-train correlation matrices and their distributions for clean (**A,B**) and noisy (**C,D**) inputs for SpikingVGG11 trained on EventMNIST dataset.

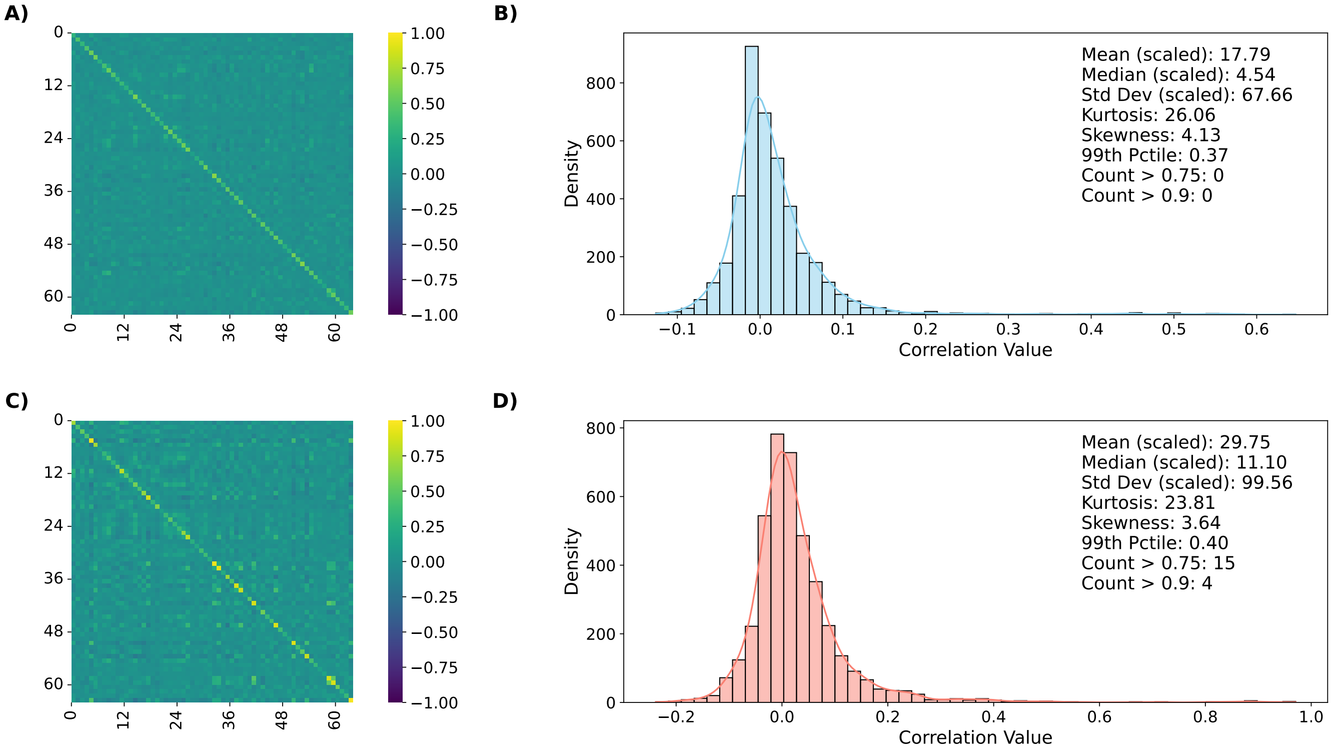

Figure S17: Average spike-train correlation matrices and their distributions for clean (**A,B**) and noisy (**C,D**) inputs for MLP-SNN trained on UCF11 dataset.

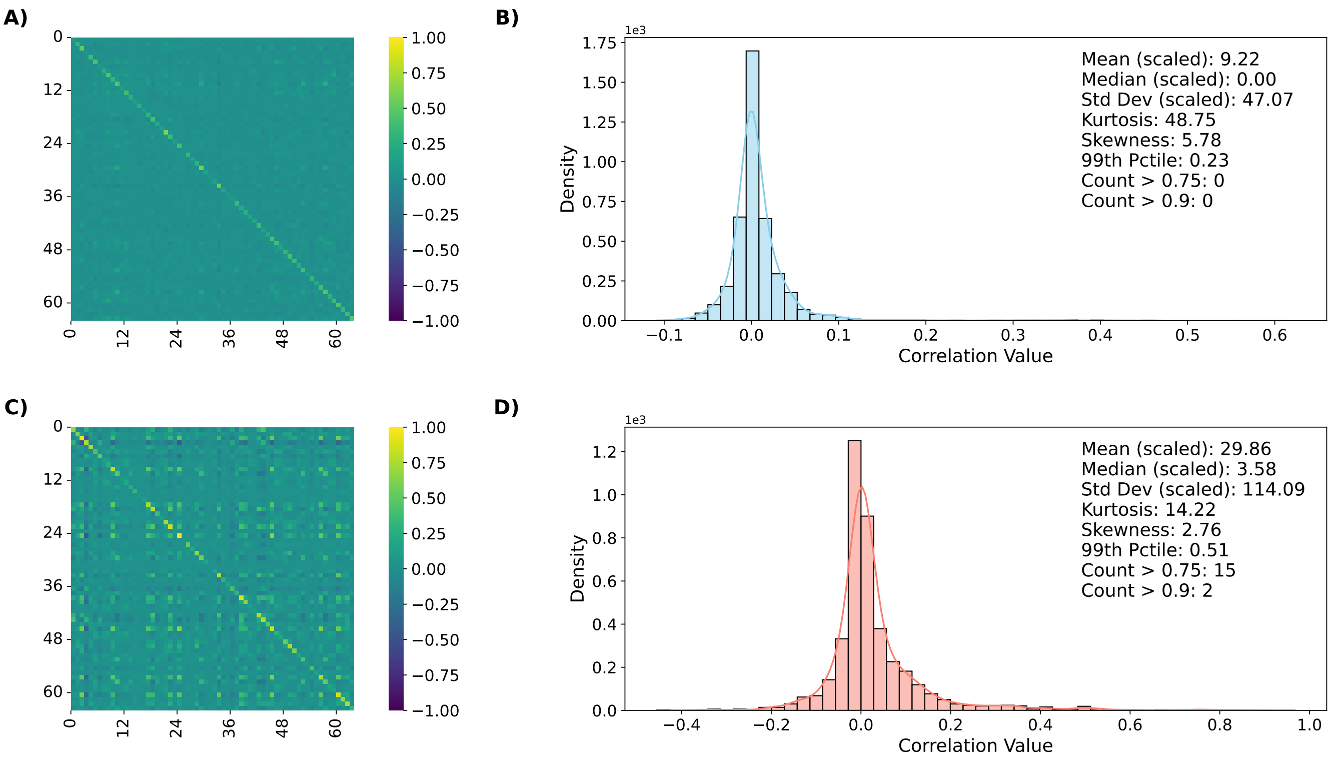

Figure S18: Average spike-train correlation matrices and their distributions for clean (**A,B**) and noisy (**C,D**) inputs for ConvSNN trained on UCF11 dataset.

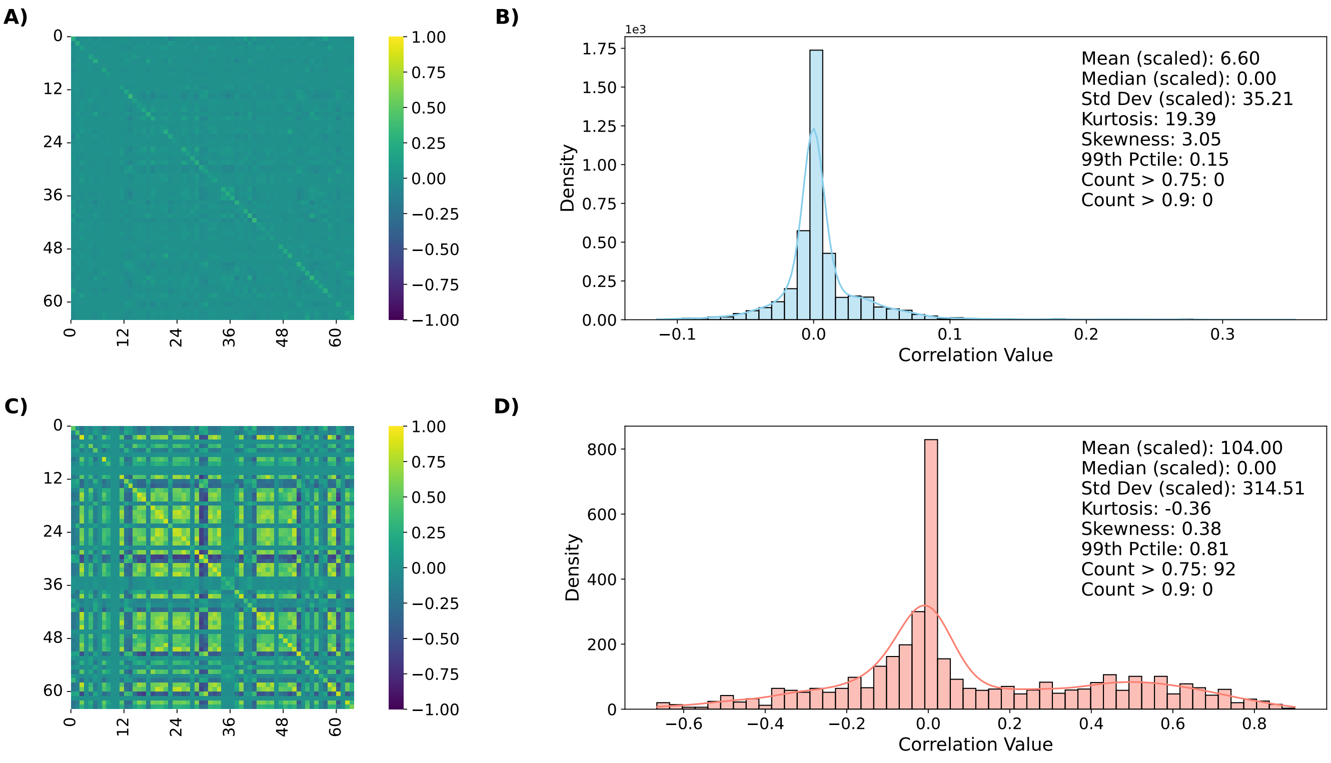

Figure S19: Average spike-train correlation matrices and their distributions for clean (**A,B**) and noisy (**C,D**) inputs for Recurrent MLP-SNN trained on UCF11 dataset.

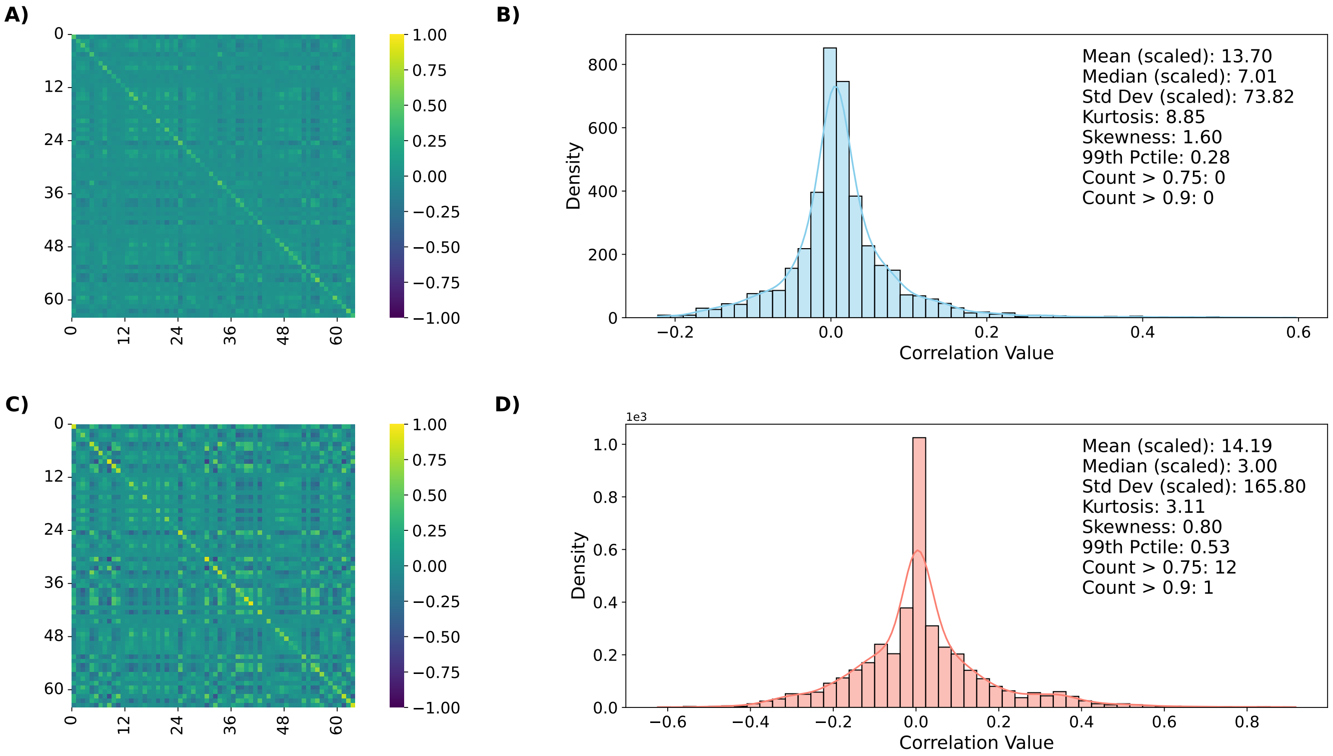

Figure S20: Average spike-train correlation matrices and their distributions for clean (**A,B**) and noisy (**C,D**) inputs for Recurrent ConvSNN trained on UCF11 dataset.

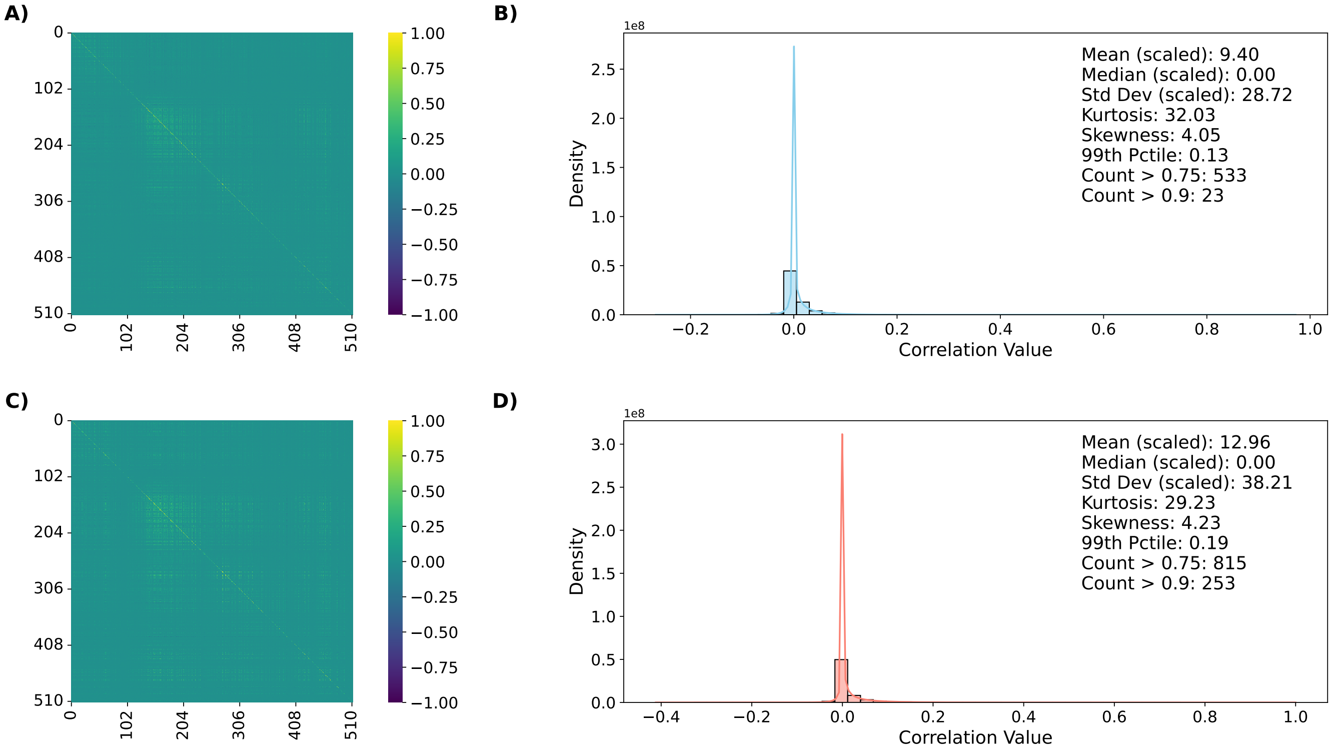

Figure S21: Average spike-train correlation matrices and their distributions for clean (**A,B**) and noisy (**C,D**) inputs for SpikingResnet18 trained on UCF11 dataset.

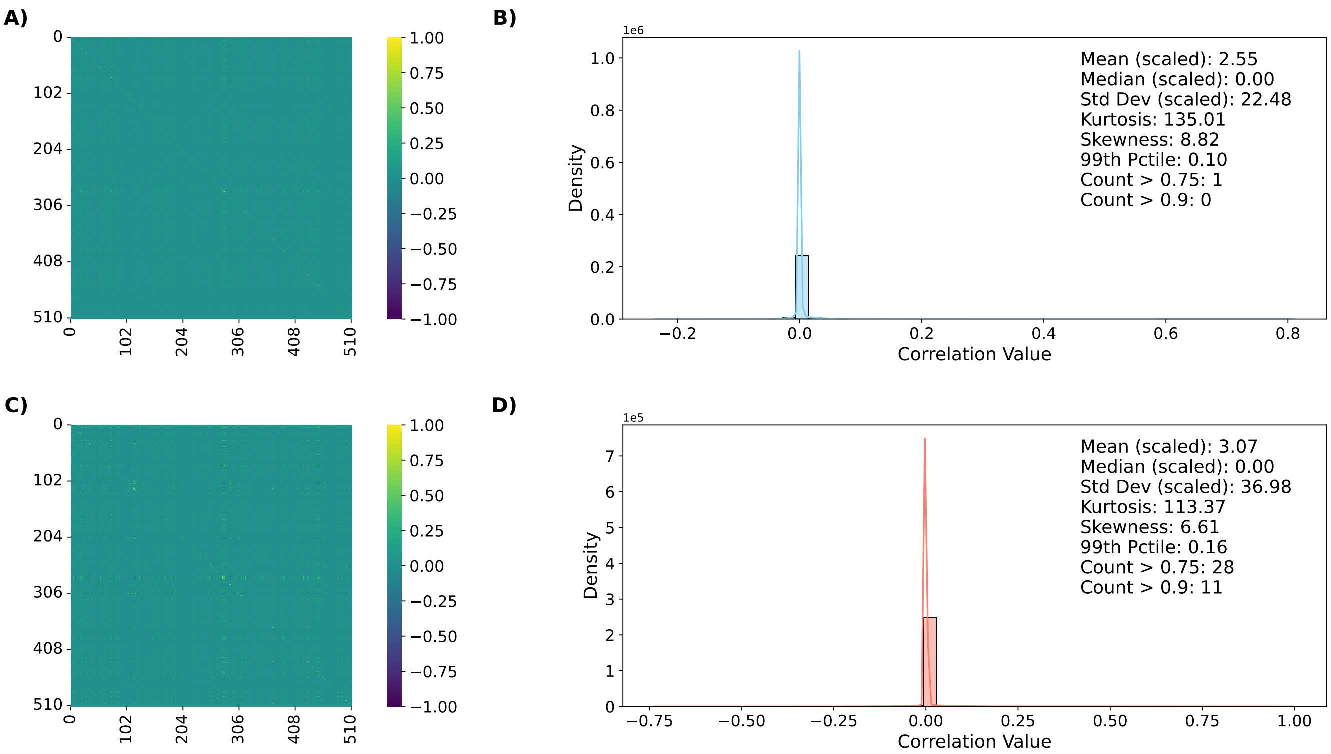

Figure S22: Average spike-train correlation matrices and their distributions for clean (**A,B**) and noisy (**C,D**) inputs for SpikingVGG11 trained on UCF11 dataset.
